# Simulation-based assessment of zwitterionic pendant group variations on the hemocompatibility of polyethersulfone membranes

**DOI:** 10.1186/s42252-024-00062-6

**Published:** 2024-09-11

**Authors:** Simin Nazari, Amira Abdelrasoul

**Affiliations:** 1 Division of Biomedical Engineering, University of Saskatchewan, 57 Campus Drive, Saskatoon, Saskatchewan, S7N 5A9 Canada; 2 Department of Chemical and Biological Engineering, University of Saskatchewan, 57 Campus Drive, Saskatoon, Saskatchewan, S7N 5A9 Canada

**Keywords:** Pendant groups, Sulfobetaine, Phosphobetaine, Carboxybetaine, Hemocompatibility, Molecular docking, Affinity energy, Protein-ligand interaction

## Abstract

In the realm of hemodialysis, Polyethersulfone (PES) membranes dominate due to their exceptional stability and mechanical properties, capturing 93% of the market. Despite their widespread usage, the hydrophobic nature of PES introduces complications in hemodialysis, potentially leading to severe adverse reactions in patients with end-stage renal disease (ESRD) through protein fouling. Addressing this issue, our study focused on enhancing hemocompatibility by modifying PES surfaces with zwitterionic materials, known for their hydrophilicity and biological membrane compatibility. We investigated the functionalization of PES membranes utilizing various zwitterions in different ratios. Utilizing molecular docking, we examined the interactions of three zwitterionic ligands—carboxybetaine methacrylate (CBMA), sulfobetaine methacrylate (SBMA), and (2-(methacryloyloxy)ethyl) phosphorylcholine (MPC)—with human serum proteins. Our analysis revealed that a 1:1 ratio of phosphobetaine and sulfobetaine exhibits the lowest affinity energy towards serum proteins, denoting an optimal hemocompatibility without the limitations associated with increased zwitterion ratios. This pivotal finding offers a new pathway for developing more efficient and safer hemodialysis membranes, promising improved care for ESRD patients.

**Supplementary information:**

The online version contains supplementary material available at 10.1186/s42252-024-00062-6.

## Research Highlights

Studying ligand-protein interactions with molecular docking as an efficient technique.

Hemocompatibility investigation of different ZWs with various pendant groups and different type of ZW-PES membranes.

Affinity energy of interactions was calculated and analyzed for ZWs, pristine, and modified PES membranes.

The hydrophilic nature of the ZW membranes was linked to their compatibility with blood.

Compared to carboxybetaine and sufobetaine, phosphobetaine showed lower affinity energy for interacting to all proteins studied.

Carbo/phospho-PES (1 :1 ratio) demonstrated the best outcomes and the lowest affinity energy among others.

A study of the effect of various pendant groups and their combinations on PES hemocompatibility has never been conducted before.

**Graphical abstract:**

**Supplementary information:**

The online version contains supplementary material available at 10.1186/s42252-024-00062-6.

## Graphical abstract

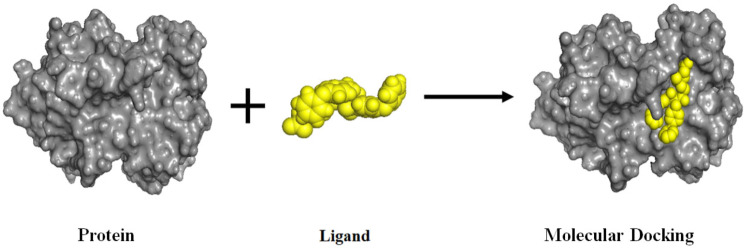

## Introduction

When the human kidney ceases to function for any reason, supportive therapies including kidney transplants, peritoneal dialysis, or hemodialysis (HD) are necessary [1–4]. In the last decade, there has been a substantial increase in the worldwide population affected by end-stage renal disease (ESRD) [5, 6] a trend that regrettably continues to grow. This has led to the expansion of the dialysis sector into a billion-dollar industry [5]. High risks for total renal failure, diabetes, tiredness, cardiovascular illnesses, and even mortality are among the major renal health conditions connected with ESRD [7–9]. With the use of membrane technology, hemodialysis has shown to be a secure separation and purification treatment for ESRD [10, 11]. These membranes function as a semipermeable barrier, allowing uremic toxins like urea, creatinine, etc. to diffuse from blood to dialysate without losing crucial blood proteins like fibrinogen, albumin, etc. [3, 12–14]. Polyethersulfone (PES) based membranes, which account for 93% of the market, are the most widely utilised HD membrane in Canadian hospitals because of their exceptional properties [15–17]. PES membranes provide excellent stability against oxidation, heat, chemicals, and mechanical stress, making them suitable for use in a range of medical devices,, artificial environmental organs, blood purification equipment, including hemodialysis, hemodiafiltration, and hemofiltration [18–21]. It is, however, avoided and prevented to use of unmodified and weakly biocompatible membranes since they increase blood coagulation cascades [22–25]. When unmodified PES-based haemodialysis membranes come into contact with blood, blood proteins have a high tendency to quickly adsorb onto the polymer surface [12, 26, 27]. The adsorption of the protein layer decreases the permeation flux and selectivity of the membrane and strongly affects HD efficiency by increased platelet attachment, depletion, and denaturation of plasma proteins, triggering the activation of biochemical cascades and rapid blood coagulation and aggregation [28–31]. This irreversible fouling may be explained by the hydrophobic-hydrophilic interactions between the membrane surface and the blood proteins [32, 33]. As the core component of the hemodialyzer, researchers in membrane technology have persistently sought straightforward additives and modification methods to enhance surface hydrophilicity. This, in turn, can lead to improvements in flux and reductions in fouling [1, 12, 34–39].

Zwitterionic polymers (ZWs), which are the last generation of hydrophilizing additives, have extensively been reported to enhance the biocompatibility and hydrophilicity of PES membrane and other biomedicine materials [19, 38, 40, 41]. Due to their electrostatic interactions, Zwitterionic materials have a strong ability to connect with water molecules and generate a hydration layer shell on their surface. The dense hydration layer effectively inhibits protein adhesion to material [40, 42–46]. However, the extent to which it fully prevents protein adsorption and thrombus formation on device surfaces relies on several factors, such as chemical composition, particularly pendant groups, as well as the density of zwitterionic groups and the thickness of the coating [47–50]. Researchers have extensively explored a variety of structures, conformations, chemical groups, ZW density, and other factors, but no comprehensive study has been conducted on comparing various pendant groups to determine which combination and ratio of these important antifouling materials will improve the efficiency of hemodialysis.

Molecular docking is a widely accepted method for forecasting the preferred alignment of one molecule to another when they form a stable complex. Scoring functions based on this preferred alignment can be employed to estimate the strength of association or binding affinity between the two molecules. Docking modelling is frequently used in the drug design, material design, and other biomedical sectors instead of demanding laborious and time-consuming experimental research. The docking software places ligands at the active sites of the proteins, and provide the protein-ligand interaction pattern by continuously optimizing the position, conformation, dihedral angle of rotatable bonds, amino acid residue side chains, and backbone of the receptor. In fact, the most suitable conformation of the interaction between the ligand and the receptor macromolecule is determined, enabling the prediction of the binding mode and affinity energy [51]. Our team has conducted extensive research on human plasma proteins as macromolecule receptor and membrane models by using molecular docking [12, 38, 52–55] providing interesting insights and information on the functional group(s) which are responsible for the interactions with human serum proteins.

In this study, the effect of different pendant groups of ZWs and their combinations on PES membrane interactions with vital human serum proteins was investigated. The investigation aimed to determine which pendant group or combination thereof provides the highest hemocompatibility on PES membranes used in renal dialysis within Canadian hospitals. Although zwitterionization of the membrane has been widely reported as an effective method to increase antifouling, biocompatibility and other functions, such as high flux and cytocompatibility, to enhance hemodialysis performance of PES membranes, this study is the first to analyze systematically how various pendant groups of ZW chains affect PES hemocompatibility. The aims of the study were delineated as follows: (i) Investigate the interactions of ligands -proteins, involving zwitterionic chains possessing various pendant groups, and three crucial human serum proteins: Fibrinogen (FB), Albumin (HSA), and Transferrin (Tr), using molecular docking techniques; (ii) Explore the impact of different ZW pendant groups and their combinations on interactions with three distinct human serum proteins; (iii) Identify the functional groups accountable for the observed interactions between carboxybetaine methacrylate (CBMA), sulfobetaine methacrylate (SBMA), and (2-(methacryloyloxy)ethyl) phosporylcoline (MPC) zwitterions, SB-PES, PB-PES, CB-PES and diblock or triblock polymers of SB/PB-PES, SB/CB-PES, PB/CB-PES with HSA, FB, and Tr human serum proteins; and (iv) Examine the impact of diverse ZW pendant groups on binding energy and affinity with human serum proteins. To our knowledge, this study represents the initial investigation into the influence of zwitterion pendant groups on the interaction between human serum proteins and PES as the most common haemodialysis membrane.

## Materials and methods

### Molecular docking simulation

**Fig. 1:**
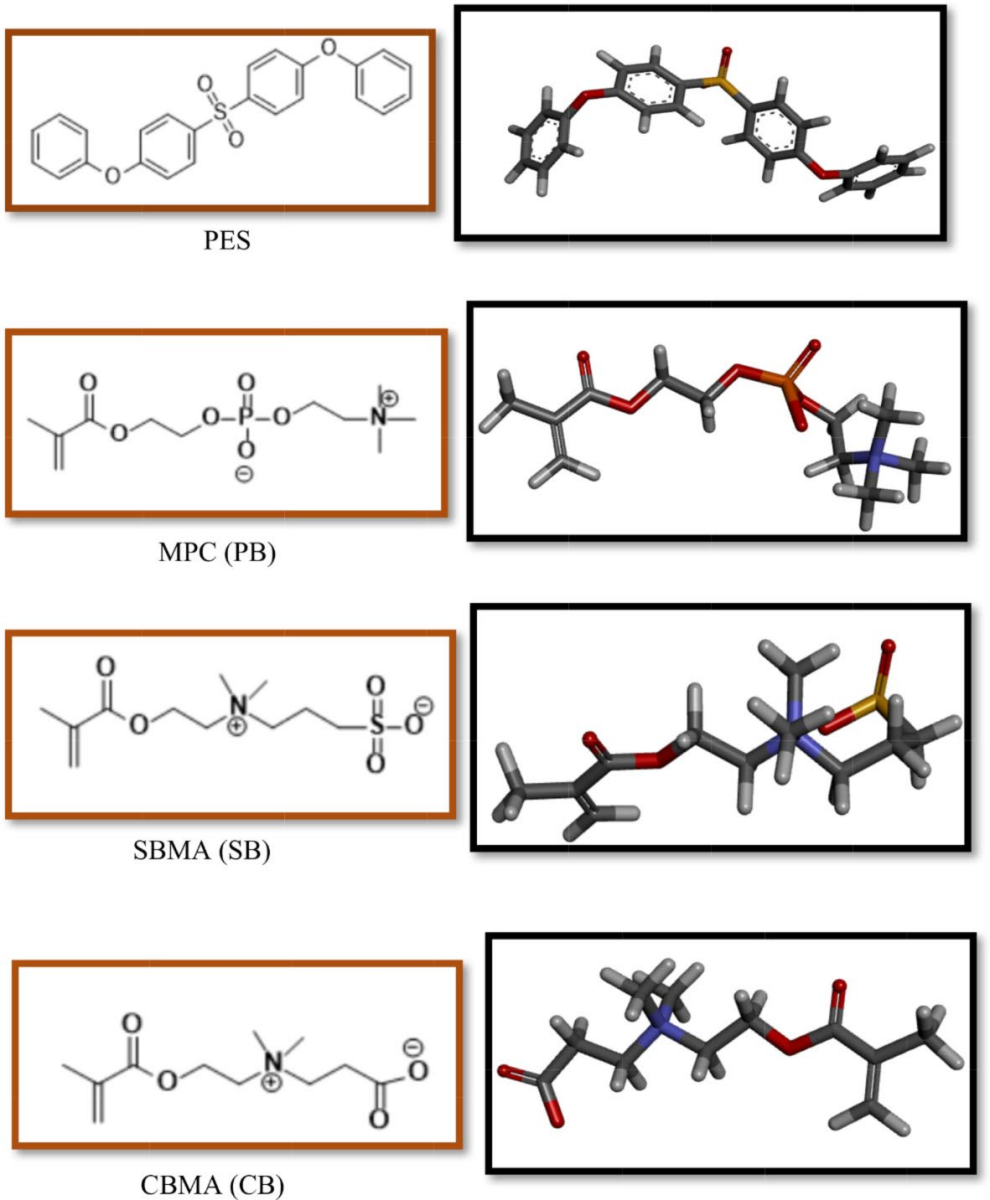
Chemical structures and stick view (with energy minimization) of polyethersulfone (PES) and zwitterion chains (PB, SB & CB)

A molecular docking technique aims to detect molecular interactions in order to generate virtual simulations based on that information. This standard computational tool is widely used in the design of biomedical devices and structure-based drug design, as it reliably predicts experimental binding modes and affinities of small molecules (ligands) within specific receptor targets (proteins). Here, carboxybetaine methacrylate (CBMA), sulfobetaine methacrylate (SBMA), and (2-(methacryloyloxy)ethyl) phosphorylcholine (MPC) zwitterionic structures served as monomer models (ligands) to investigate the impact of different pendant groups on receptor-ligand interactions and PES hemocompatibility. RCSB protein data bank (PDB) provided the 3D X-ray structures of HSA (PDB code: 1AO6), FB (PDB code: 3GHG), and TRF (PDB code: 1D3K) proteins for docking analysis. Chemdraw software was used for drawing the structures and Chem3D Ultra was used for energy minimization. Then the Chemdraw format for ligands was converted to PDB format through Pymol which is a powerful molecular viewer with exceptional 3D capabilities. For the purpose of determining favourable structural characteristics for protein-ligand interactions, docking studies were performed using AutoDock software version 4.0. The protein structure was converted to PDBQT format after removing water molecules, merging nonpolar hydrogens, and adding Kollman charges using AutoDock tools. The active sites of the proteins were shown in a docking box 40*40*40 in the centre of the proteins, and to maximise efficiency, residues containing atoms larger than 7 Å were excluded from the grid box. Lamarckian Genetic Search Algorithm (LGA) with a run set of 15 was used here to ensure data accuracy. By measuring the intermolecular energy, docked proteins and zwitterionic ligands were evaluated for their interaction forms. It should be noted that ChemDraw, PyMOL, and Discovery StudioVisualizer were used to display all minimized energy conformers. Figure 1. 1illustrates the structures, optimized and energy-minimized conformers of the used zwitterions and pristine PES membrane. While the molecular docking simulations provide valuable insights into the potential interactions between individual PES molecules and zwitterions, it is important to acknowledge that PES, in its practical form as a membrane, is a complex polymer with entangled chains rather than free molecules. Therefore, the simulations focused on simplified models to explore fundamental interactions. This approach was chosen to guide the design of more effective hemodialysis membranes, though further experimental validation is required to fully understand the implications in real-world conditions.

The molecular docking simulations in this study were performed under the assumption that ZWs, although modeled as individual entities, would be immobilized on the PES surface through these chemical bonds. This approach was taken to explore the fundamental interactions between ZW pendant groups and proteins, providing a preliminary estimate of their potential influence on hemocompatibility.

The proteins chosen for this study—Fibrinogen (FB), Albumin (Alb), and Transferrin (Tr)—were selected based on their critical roles in hemodialysis and their significant impact on hemocompatibility. Fibrinogen is essential for blood clotting, and its interaction with membrane surfaces can initiate clotting cascades, which compromises membrane performance. Albumin, being the most prevalent plasma protein, plays a vital role in maintaining oncotic pressure and influences membrane biocompatibility. Transferrin, while less abundant, offers insights into iron transport and membrane interactions. Although these proteins provide valuable insights into the effects of zwitterionic modifications, future studies could include a broader array of serum proteins to further verify and expand upon the findings related to membrane hemocompatibility.

## Results and discussion

### PES interactions with human serum proteins

Understanding the interactions between biomaterial surfaces and biological fluids, such as blood and interstitial fluid, is crucial for developing insights into blood-material interactions and discovering novel therapeutic approaches. Computational strategies are powerful instruments that have permeated all aspects of biomedical device design and drug discovery today. Blood is induced to form thrombus when exposed to foreign materials, particularly those without hemocompatible top layers [24, 38, 56]. The protein layer initially adhering to the biomaterial surface, particularly on HD membranes, largely triggers negative responses. These include the activation of coagulation via the intrinsic pathway, leukocyte activation resulting in inflammation, and platelet adhesion and activation [57]. Therefore, biomaterials designed for blood contact should not exhibit adverse interactions with blood components nor provoke their activation or degradation. Hence, it is essential to thoroughly assess potential adverse interactions between newly developed materials and blood to mitigate the activation and degradation of blood components. Albumin (HSA or ALB), fibrinogen (FB), and transferrin (TR) are three significant plasma proteins that have an affinity for adsorption. Membrane hemocompatibility may be influenced by the conformation of adsorbed proteins on their surfaces. Molecular docking is extensively employed for identifying optimal sites, orientations, interactions, and binding energies between macromolecules (specifically proteins, referred to as targets) and ligands in computational modeling [58]. This study scrutinized the influence of a variety of zwitterionic structures on PES hemocompatibility by docking HSA, FB, and TR on pristine and modified PES membranes. The affinity energy and most favourable membrane-protein interactions were analyzed by examining different pendant groups and their ratios. Figure 1 shows the structures and energy-minimized conformers of PES and zwitterionic compounds. The 3D images and electrostatic maps depicting the docking interactions between PES and the proteins HSA, FB, and TR, as well as a summary of all PES interactions and affinity energies, can be referenced in our previous publication [59]. The docking of the PES model structure into the protein active site demonstrates that while phenyl groups are covered by hydrophobic groups, polar parts like etheric and SO_2_ groups are introduced into the hydrophilic pocket of proteins through hydrophilic contacts (Fig. S1. Supplementary Material). The result illustrated that docking PES with albumin had highest binding energies of -10.3 kcal/mol in contrast to the two other proteins, and also showed PES fit more easily into the hydrophobic cavity of each protein [59].

### Effect of ZW pendant groups on interactions with human serum proteins

**Fig. 2:**
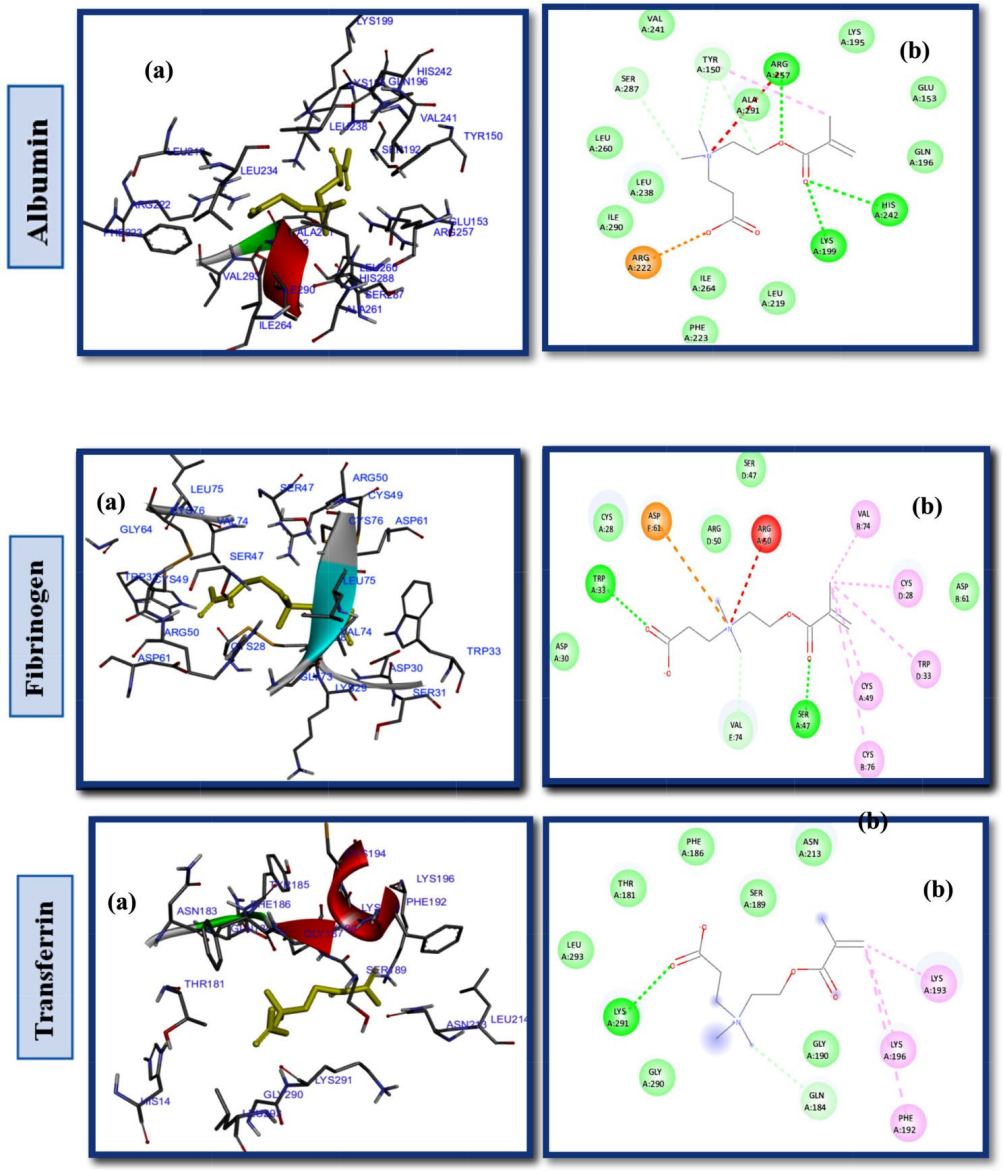
(**a**) 2D interaction diagrams, and (**b**) Electrostatic interaction profiles for the docking of CB with HSA, FB and TR

**Fig. 3:**
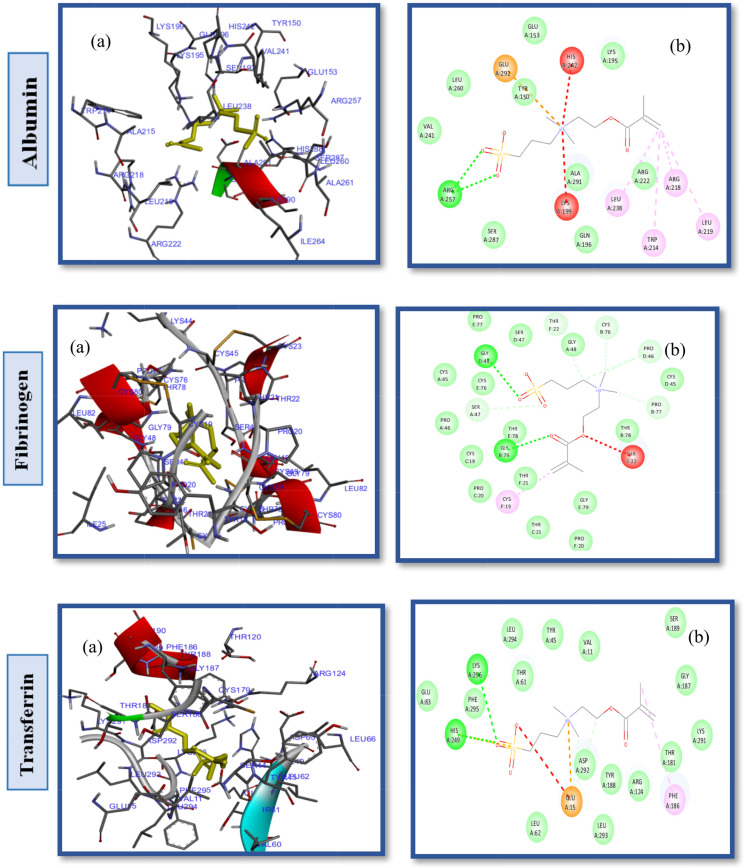
(**a**) 2D interaction diagrams, and (**b**) Electrostatic interaction profiles for the docking of SB with HSA, FB and TR

**Fig. 4:**
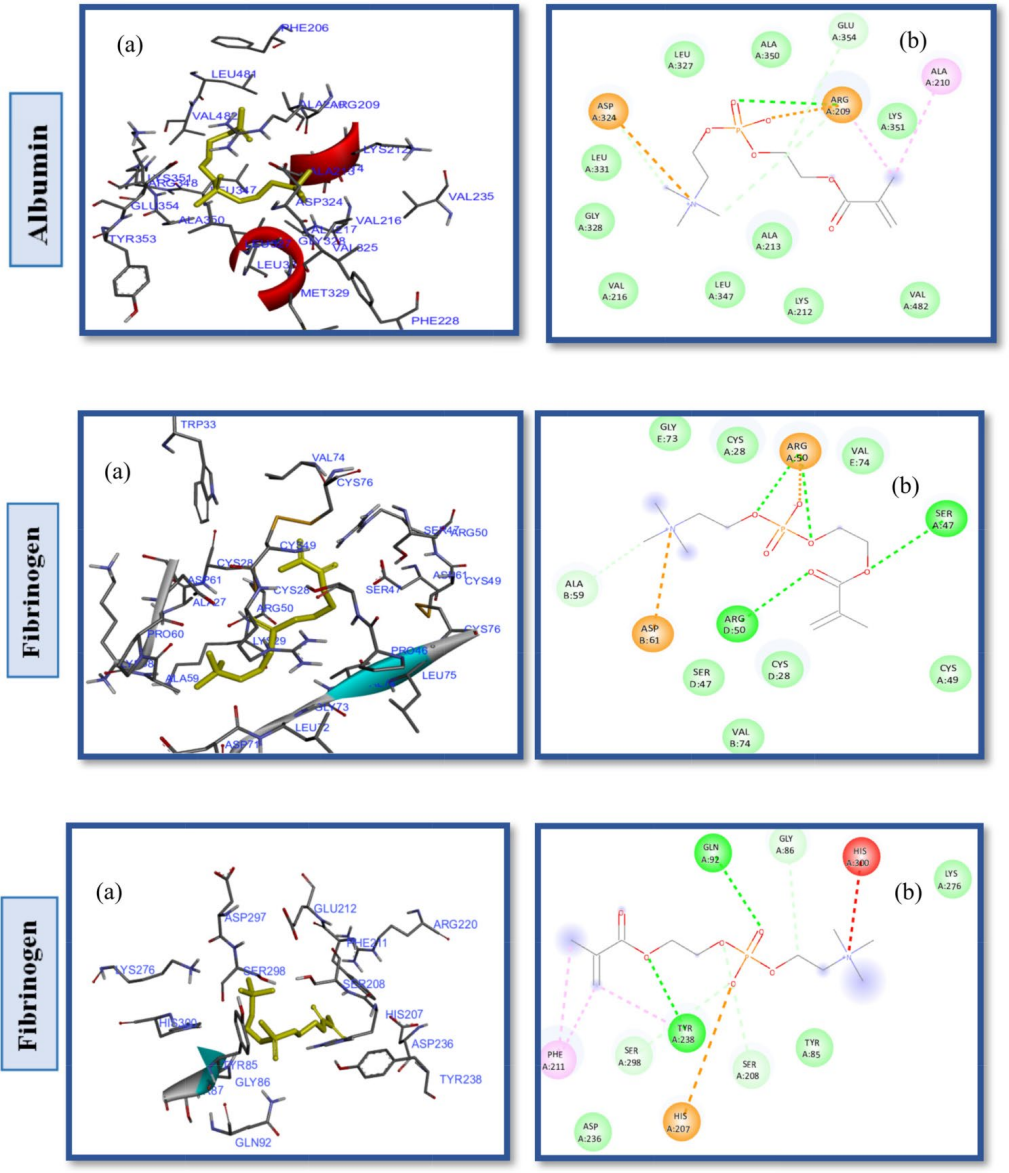
(**a**) 2D interaction diagrams, and (**b**) Electrostatic interaction profiles for the docking of PB with HSA, FB and TR

Studies of protein-ligand interactions provide a theoretical basis for developing novel therapeutic targets and biomedical devices as well as a deeper comprehension of biological regulation. A number of interactions commonly observed in ligand design include hydrophobic contacts, hydrogen bonds and π-stacking. Afterward, hydrogen bonds, salt bridges, amide stacking, and cation-cation interactions are also frequently formed [60–62]. Enhancing the hydrophilic properties of hemodialysis (HD) membrane surfaces through the application of zwitterionic materials (ZWs) augments their resistance to the adsorption of proteins, which are indicative contaminants in the context of organic fouling scenarios. In order to determine the impact of pendant groups on ZW interactions and their affinity energies, as well as the hemocompatibility of ZW-PES, molecular docking on the proposed ZWs was performed first, before docking of zwitterionized PES membrane models. Figures 2 and 3, and 4 depict the docking results of the PB, SB, and CB with the HSA, FB, and Tr. Pymol and the Discovery studio visualizer show that the interaction patterns and affinity binding energy for these zwitterionic structures differ substantially. Since the chemical structures of ZWs and their optimized conformers are similar, the influence of pendant groups can be attributed directly to this variation. Docking results revealed that PB had lower affinity energies for all of the ligand-protein interactions when compared to CB and SB. As shown by Table 1, phosphobetaine binds to the active sites of HSA, FB, and Tr. with affinity binding of -5.2, -4.8, and − 4.6 kcal mol^− 1^, respectively. In contrast, carboxybetaine binds with affinity energy of -5.5, -5.1, and − 4.7 kcal mol^− 1^, and sulfobetaine exhibited the highest affinity energy at -5.9, -5.6, and − 5.6 kcal mol^− 1^. The superior affinity energy observed for SB is attributable to the distribution of the sulfonate anions’ negative charge across a greater number of oxygen atoms compared to carboxylate anions, which effectively delocalizes the charge and contributes to a higher affinity. Consequently, water molecules exhibit a heightened attraction to SB moieties, which possess a more dispersed charge distribution, as opposed to CB moieties that are characterized by greater charge densities. Conversely, CB polymers interact with water molecules more effectively and strongly than SB compounds [63] in a restricted number of cases. There is evidence that having a few tightly bound water molecules is more effective at resisting nonspecific adsorption of proteins than having many loosely bounds [63, 64]. Additionally, the propensity for self-association is higher in SB compounds relative to CB moieties, likely due to the sulfonate groups having an increased charge density in comparison to carboxylate groups. Conversely, CB zwitterions, which exhibit lower self-association tendencies, are less prone to non-selective protein adsorption, rendering them more compatible with blood (hemocompatible) than their SB counterparts. In phosphate, since charge distributions in phosphate and carboxylate are similar, electronegativity differences between P-O and C-O bonds provide useful information about their binding affinity nergies. Electronegativity differences in P-O bonds (1.25) are larger than in C-O bonds (0.89 ), indicating oxygen atoms in phosphates have larger partial negative charges than oxygen atoms in carboxylates, which in turn leads to stronger hydrogen bonding and hydrogen shells as well as lower affinity binding energy in phosphate groups. HSA, a globular, heart-shaped protein made up of 67% -helices and 585 amino acids, had higher affinity binding energy in docking with all ZWs (PB, SB, and CB) than FB and Tr. The amino acids: Arg257, Lys199, His242, Arg222, Lys195, Glu153 and Ser287 interact in a hydrophilic manner between CB and HSA, as presented in Fig. 2. and Table 1. Furthermore, the Gln196, Leu219, ILE264, Phe223, ILE290, Leu238, Leu260, Tyr150 and Val241 were engaged in hydrophobic interaction with CB (Fig. S2. Supplementary Material). While, SB had polar interactions with HSA via Arg257, Glu292, His242, Lys199, Arg222, Ser287, Glu153 and Lys191, which occur from different polar positions (Fig. S3. Supplementary Material). Leu219, Trp214, Leu238, Gln196, Ala291, Val 241, Leu260 and Tyr150 are hydrophobic interactions of SB-HSA as shown in Fig. 3. The docking results of the PB model with the HSA protein (Fig. 4) indicates that the amino acids Arg209, Asp324, Lys351, Glu354, and Lys212 are engaged in hydrophilic interactions, while, Val482, Ala213, Leu347, Val216, Gly328, Leu331, Leu327, Ala350 and Ala210 were responsible residues in hydrophobic interactions (Fig. S4. Supplementary Material).

As can be seen in the 2D interactions patterns of docking CB and HSA, hydrogen bonds formed between the oxygen atoms of the acrylate parts and the N atoms of the ammonium groups and Arg257, Lys199, and His242. Additionally, in this model a salt bridge, that keeps the ligand-protein in the closed conformation, developed between the oxygen atom of the carboxylate groups and Arg222. While in the PB model, hydrogen bonds have been formed between the O atoms of the phosphate groups and Arg209. Even though it seems that CB has more and stronger hydrogen bonds than PB, which normally results in a stronger hydrophilicity shell and higher antifouling property, it displayed higher affinity energy than PB for binding to all examined proteins. It is unlikely that any single parameter or chemical factor explains antifouling performance by itself, but rather a variety of factors contribute to it. The difference of energy affinty for CB and PB may be a result of combined effects of electrostatic and hydrophobic interactions. In the case of PB and HSA, Arg209 forms attractive charge electostatic interactions, a powerful form of contact, as well as hydrogen bonds with O atoms of phosphate groups. Therefore, Arg209 and PB have a strong link, which may result in a strong hydrogen shell forming around PB, giving it more fouling resistance and lower affinity binding energy than CB. These outcomes are in line with other research findings that have been reported in the literature [65, 66]. Additional docking results and interactions between HSA, FB, and TR with CB, SB, and PB are listed in Table 1. It should be noted that different pendant groups of ZW structures have also led to distinct patterns of interactions with other proteins (FB and TR), leading to various hydrophobic and hydrophilic contacts and different affinity energies, as shown in Table 1.

While our study primarily employs molecular docking simulations, which are based on static structures, it is important to recognize that proteins in biological systems are dynamic and can undergo conformational changes. These dynamic interactions are not fully captured by static docking simulations. Despite this limitation, the current study provides valuable insights into the effects of zwitterionic pendant groups on protein interactions under controlled conditions, allowing for a comparative analysis of their potential impact on hemocompatibility.

To address this limitation and gain a more comprehensive understanding of protein interactions in a dynamic biological environment, we plan to incorporate molecular dynamics (MD) simulations in future studies. MD simulations will enable us to model the time-dependent behavior of proteins and offer a more detailed view of their interactions with zwitterionic materials, bridging the gap between static and dynamic states.

### Interactions of ZW-PES membrane models with human serum proteins

**Fig. 5:**
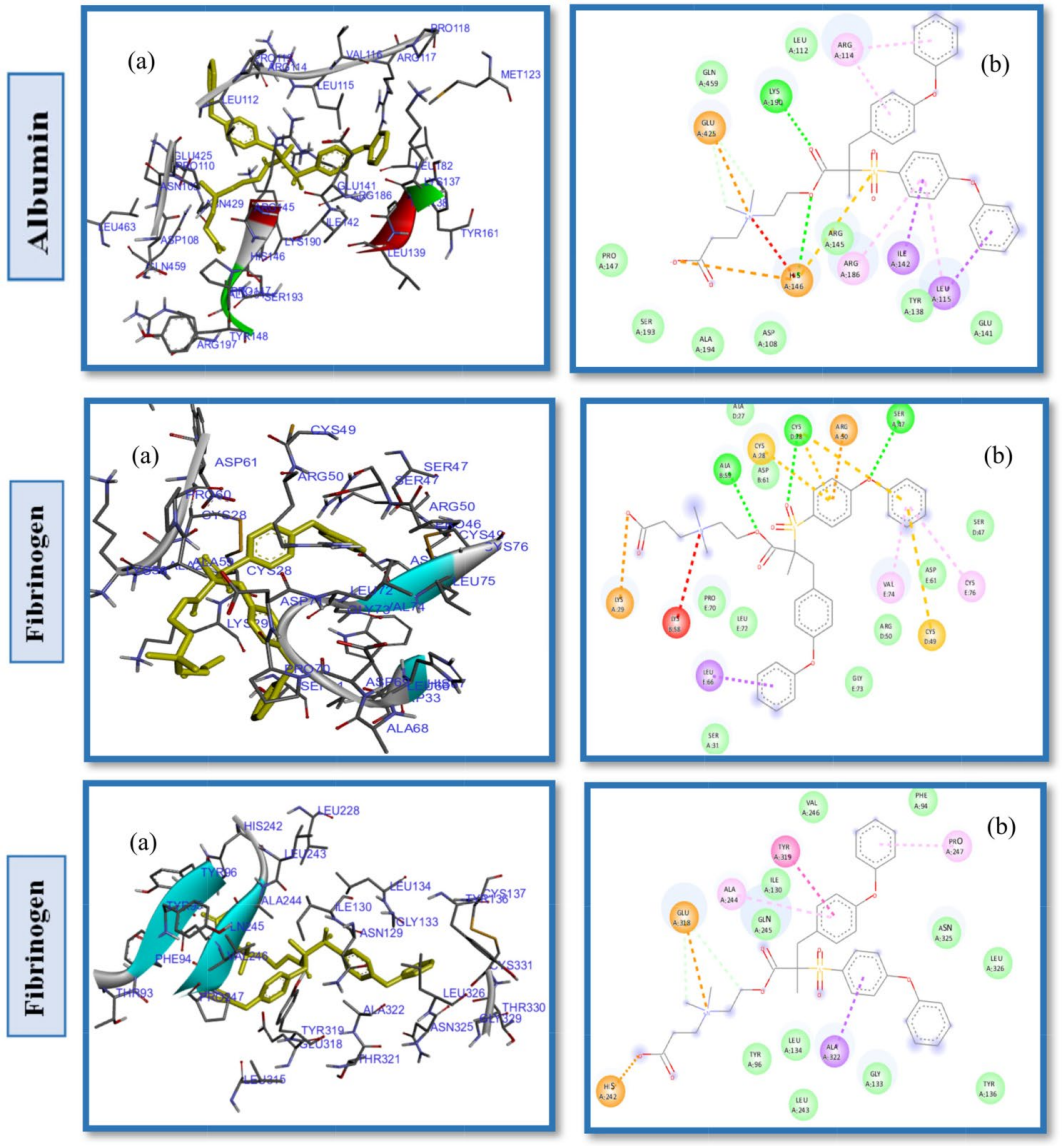
(**a**) 2D interaction diagrams, and (**b**) electrostatic maps of interactions of docking CB-PES with HSA, FB and TR

**Fig. 6:**
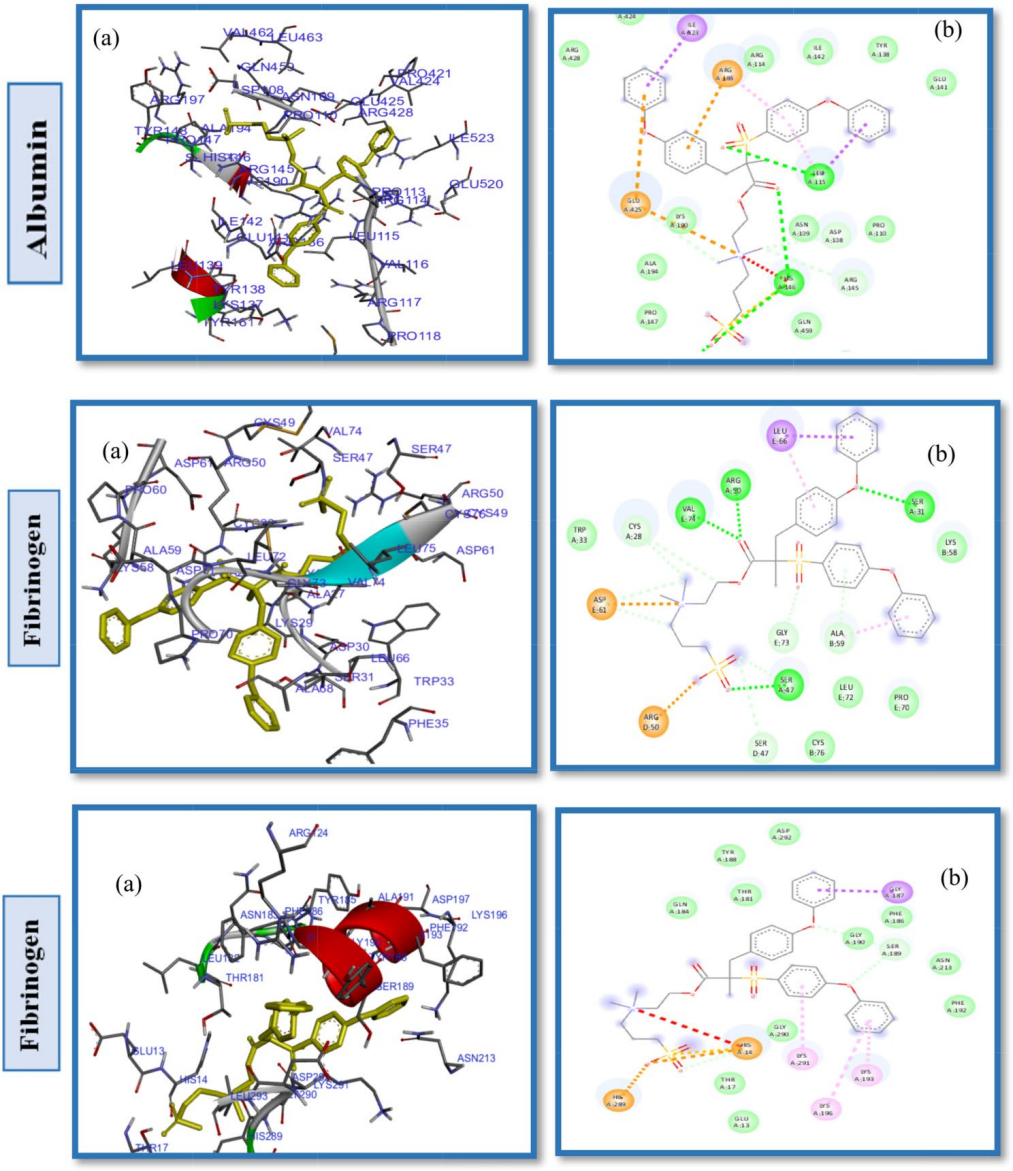
(**a**) 2D interaction diagrams, and (**b**) electrostatic maps of interactions of docking SB-PES with HSA, FB and TR

**Fig. 7:**
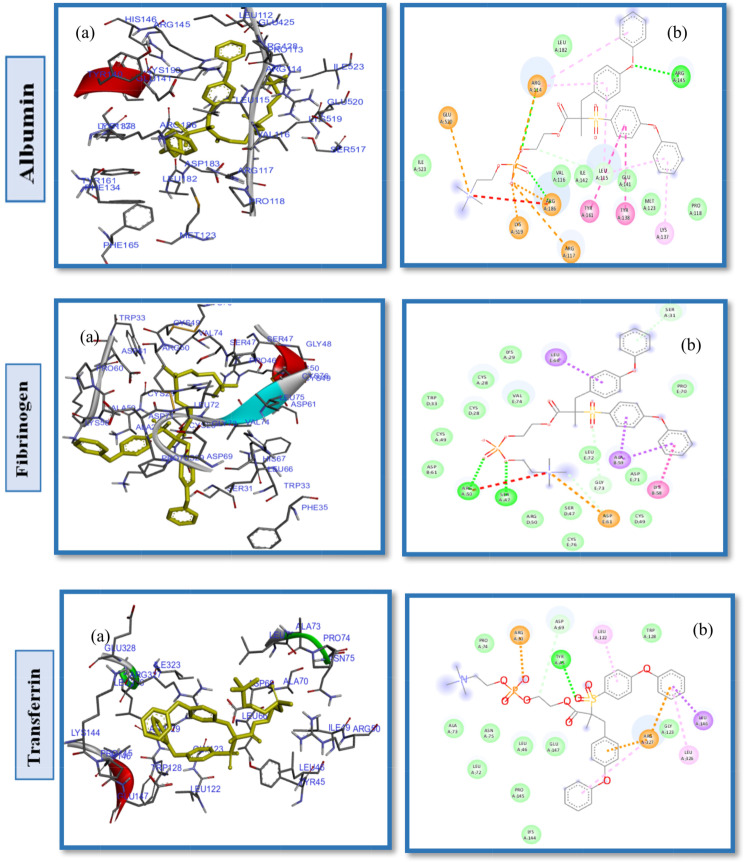
(**a**) 2D interaction diagrams, and (**b**) Electrostatic interaction profiles for the docking of PB-PES with HSA, FB and TR

The computational study was designed to evaluate the interactions between CB-PES, SB-PES, and PB-PES membrane models and HSA, FB, and TR proteins to ascertain the influence of these ZWs with differing negative groups on membrane hemocompatibility. Docking analysis indicates that the SB-PES model exhibited greater binding affinity compared to the CB-PES or PB-PES models, with varying interaction patterns observed across different proteins (Figs. 5, 6 and 7; Table 2). There are two types of significant interactions that take place when ZW-PES membranes are docked into protein active sites: (1) polar or hydrophilic interactions caused by ZW and PES etheric and SO_2_ groups; and (2) non-polar or hydrophobic interactions brought on by protein chains and the phenyl groups of PES membrane.

The interaction analysis between the CB-PES model and the HSA protein (Fig. 5) reveals that the amino acids Lys190, His146, Arg145, Asp108, Glu141, Glu426, His146, Arg145, Arg114, and Arg186 engage in hydrophilic interactions. Conversely, hydrophobic interactions are attributed to Leu115, Ile142, Tyr138, Ala194, Ser193, Pro147, Gln459, and Leu112 (Fig. S5. Supplementary Material). The introduction of carboxybetaine significantly enhances PES fouling resistance by lowering the interaction affinity energy with the HSA protein to -8.7 kcal/mol, as observed in Table 2, entry 1, in contrast to the − 10.3 kcal/mol noted with PES ligands and HSA proteins [59]. Similarly, the SB-PES model’s docking into the HSA active site indicated that both hydrophobic and hydrophilic interactions are pivotal in the protein-ligand interaction dynamics. Specifically, the amino acids Tyr138, Ile523, Val424, Ala194, Pro147, Val462, Glu459, Asn109, and Pro110 are involved in hydrophobic interactions with SB-PES, whereas His146, Ser193, Leu115 form.

**Table 1:** Binding energy outcomes and receptor interactions of HSA, FB, and TR with CB, SB, and PB zwitterions

Ligand	Protein	Binding energy (kcal/mol)	Receptor contacts Hydrophilic Hydrophobic	
CB	HSA	-5.5	Arg257^a^, Lys199^a^, His242^a^, Arg222^b^, Lys195^c^, Glu153^c^, Ser287^c^	Gln196, Leu219, Ile264, Phe223, Ile290, Leu238, Leu260, Tyr150, Val241
CB	FB	-5.1	Ser47^a^, Trp33^a^, Asp61^c^, Asp30^c^, Arg50^d^, Asp61^e^	Val74, Cyc28, Trp33, Cyc49, Cys76, Cyc28, Ser47
CB	TR	-4.7	Lys291^a^, Lys193^f^, Lys196^f^	Phe192, Gly190, Gln184, Gly290, Leu293, Thr181, Phe186, Ser189, Asn213
SB	HSA	-5.9	Arg257^a^, Arg222^c^, Ser287^c^, Glu153^c^, Lys191^c^, His242^d^, Lys199^d^, Glu292^e^	Leu219, Trp214, Leu238, Gln196, Ala291, Val241, leu260, Tyr150
SB	FB	-5.6	Gly48^a^, Gly79^a^, Ser47^c^, Cys45^c^, Thr22^d^	Gly79, Cyc19, Thr21, Pro20, Thr78, Pro46, Pro77, Thr22, Gly48, Cyc76
SB	TR	-5.6	His249^a^, lys296^a^, Glu83^c^, Lys291^c^, Arg124^c^, Asp292^c^, Clu15^e^	Gly187, Thr181, Phe186, Tyr188, Leu293, Leu62, Phe295, Leu294, Thr61, Tyr45, Val11
PB	HSA	-5.2	Arg209^a^, Lys351^c^, Glu354^c^, Lys212^c^, Asp324^e^	Val482, Ala213, Leu347, Val216, Gly328, Leu331, Leu327, Ala350, Ala210
PB	FB	-4.8	Arg A:50^a^, Arg D:50^a^, Ser A:47^a^, Arg a:50^e^, Asp61^e^	Cys49, Cys28, Val74, Ser D:47, Ala59, Gly73, Cys28
PB	TR	-4.6	Gln92^a^, Tyr238^a^, Asp236^c^, Lys276^c^, His300^d^, His207^e^	Gly86, Tyr85, Ser208, Ser298, Phe211

a: Conventional Hydrogen Bond b: Salt Bridge

c: weak Van der Waals d: Unfavorable Positive-Positive.

e: Attractive Charge f: Pi-Alkyl.

hydrogen bonds (Fig. S6. Supplementary Material), and Asp108, Arg145, Arg197, Lys190, Arg114, Glu141, His146, Arg186, and Glu425 participate in van der Waals, repulsive charge, and other polar contacts (illustrated in Fig. 6). Although the SB modification significantly benefits PES hemocompatibility, it does not achieve the efficacy of SB alone due to the hydrophobic interactions within PES, as deduced from the comparative binding energy values. Moreover, docking experiments with various proteins showed that SB-PES ligands exhibit a higher affinity energy than both CB-PES and PB-PES, suggesting a reduced fouling resistance for this ligand.

Further docking studies with PB-PES and HSA were conducted to evaluate the influence of the phosphate anion and the role of different pendant groups within ZW chains on PES hemocompatibility, as depicted in Fig. 7. These studies identified Pro118, Met123, Tyr138, Tyr161, Ile142, Val116, Ile523, Leu182 as key residues in hydrophobic pockets and revealed interactions through hydrogen bonds with Arg145, and Arg186, among other hydrophilic and electrostatic interactions (Fig. S7. Supplementary Material). The binding energy for PB-PES interactions with HSA was recorded at -8.6 kcal/mol (Table 2, entry 7), showcasing the phosphate anion’s positive effect on improving PES hemocompatibility.

The antifouling mechanism of zwitterionic polymers is fundamentally based on the water barrier principle, wherein zwitterionic ion pairs electrostatically attract water molecules to form a densely packed and stable water layer over the polymer brushes. This phenomenon is essential for understanding polymer-protein interactions and depends on the zwitterionic brushes’ packing density and surface chemistry attributes, such as the balance between hydrophobicity and hydrophilicity, charge distributions, and zwitterionic group interactions. The presence of charged amino acids in proteins, including HSA, FB, and TR, plays a crucial role in these interactions by engaging with the ligands’ active sites in varied manners, thereby affecting the electrostatic interactions significantly. This leads to differences in energy affinity among zwitterionic models, which is influenced by factors like dipole density, charge distribution, carbon spacer length, conformation, pendant group type, and local dielectric properties, ultimately impacting the ligands’ compatibility and fouling characteristics. These insights corroborate the hypothesis presented in the study [66–69] .

**Table 2:** Binding energy outcomes and receptor interactions of HSA, FB, and TR with CB-PES, SB-PES, and PB-modified PES

Ligand	Protein	Binding energy (kcal/mol)	Receptor contacts Hydrophilic Hydrophobic	
CB-PES	HSA	-8.7	Lys190^a^, His146^a^, Arg145^b^, Asp108^b^, Glu141^b^, Glu426^d^, His146^d^, Arg145^d^, Arg114^e^, Arg186^e^	Leu115, Ile142, Tyr138, Ala194, Ser193, Pro147, Gln459, Leu112
CB-PES	FB	-7.2	Ser47^a^, Cyc28^a^, Ala59^a^, Asp E:61^b^, Arg D:50^b^, Asp B:61^b^, Lys58^c^, Arg A:50^d^, Lys29^d^	Cys76, Cys49, Val74, Gly73, Ser31, Leu66, Leu72, Pro70, Ala27
CB-PES	TR	-6.5	Glu318^d^, His247^d^	Pro247, Phe94, Val246, Tyr319, Ile130, Ala244, Gln245, Tyr96, Leu243, Ala322, Gly133, Tyr136, Asn325
SB-PES	HSA	-9	His146^a^, Ser193^a^, Leu115^a^, Asp108^b^, Arg145^b^, Arg197^b,^ Lys190^b^, Arg114^b^, Glu141^b^, His146^c^, Arg186^d^, Glu425^d^	Tyr138, Ile523, Val424, Ala194, Pro147, Val462, Gln459, Asn109, Pro110
SB-PES	FB	-7.5	Ser31^a^, Arg50^a^, Val74^a^, Ser A:47^a^, Ser D;47^b^, Gly73^b^, Lys58^b^, Arg50^d^, Asp61^d^	Pro70, Leu72, Cys76, Ala59, Leu66, Cys28, Trp33
SB-PES	TR	-7.7	Glu13^b^, Gly190^b^, Ser189^b^, Asp292^b^, His28^d^, His14^d^, Lys291^e^, Lys196^e^, Lys193^e^	Tyr188, Thr181, Gln184, Gly187, Phe186, Asn213, Phe192, Gly290, Thr17
PB-PES	HSA	-8.6	Arg145^a^, Arg186^a^, Glu141^b^, Glu520^d^, Arg114^d^, Lys519^d^, Arg117^d^, Lys137^e^	Pro118, Met123, Tyr138, Tyr161, Ile142, Val116, Ile523, Leu182
PB-PES	FB	-7.1	Arg50^a^, Ser A:47^a^, Lys58^b^, Asp71^b^, Asp B:61^b^, Lys29^b^, Asp E:61^d^	Ser31, Leu66, Pro70, Val74, Cys28, Ala59, Leu72, Gly73, Cys49, Cys76, Ser D:47, Trp33
PB-PES	TR	-6.3	Tyr45^a^, Asp69^b^, Glu147^b^, Arg327^d^, Arg50^d^	Trp128, Leu122, Pro74, Leu326, Gly123, Pro145, Leu46, Leu72, Ala73, Asn75

a: Conventional Hydrogen Bond

b: Weak Van der Waals c: Unfavourable Positive-Positive.

d: Attractive Charge e: Pi-Alkyl.

In the next step, the number of ZWs on the PES was increased by a factor of two and three to examine the effect of packing density on membrane hemocompatibility. Docking studies were conducted between the all examined proteins (HSA, FB, and TR) and the PES that had been modified with 2 and 3 mol of each ZWs (CB*2-PES, SB*2-PES, PB*2-PES, CB*3-PES, SB*3-PES And PB*3-PES). When ZWs are doubled to modify PES, affinity energy decreases significantly, resulting in good fouling resistance. The results reveal a positive correlation between ZW density and antifouling properties of modified PES models, ordered in a decreasing order of SB*2-PES > CB*2-PES > PB*2 PES, which support the trend seen when using 1 mol of ZWs. Even though there was a decrease in affinity energy when the number of ZWs increased to 3, the difference in affinity between 2 and 3 mol of ZWs is smaller than that between 1 and 2 and in some cases we observe the growth of affinity energy. It is anticipated that the differences in affinity energy between using 2 and 3 mol ZWs in membrane modification will be smaller than this under experimental settings due to the increasing roughness. In perspective of experiments, surface roughness is always a feature that affects how well proteins interact with surfaces. Foulants can accumulate in valleys in membranes with rough surfaces, which prevents hydrodynamic force from removing them. It should be noted that while the models were designed with perfect flatness from a modeling perspective to negate any effects of surface roughness on surface hydration and protein resistance [67], ZW-PES ligands containing 2 moles of zwitterion demonstrated the most favorable outcomes. Moreover, increasing the ZWs from 2 to 3 did not significantly enhance fouling resistance. The pattern was almost identical to the previous ones, with SB*3-PES having the highest binding energy and PB*3-PES having the lowest affinity energy. All the affinity energies and protein-ligand interactions between modified PES with 2 & 3 mol ZWs and proteins were summarized in Table 3.

**Table 3:** Binding energy outcomes and receptor interactions between HSA, FB, and TR and ligands (ZW-modified PES with 2 and 3 moles of CB, SB, and BP zwitterions)

Ligand	Protein	Binding energy (kcal/mol)	Receptor contacts Hydrophilic Hydrophobic	
CB-CB-PES	HSA	**-8.4**	Lys190^a^, His146^a^, Lys519^b^, Arg186^b^, Arg197^c^, Asp108^c^, Glu425^c^, Arg145^c^, Arg117^d^, Arg114^e^, Glu425^e^	Val116, Ile142, Leu115, Tyr138, Tyr161, Met123, Ile523, Leu463, Ser193, Gln459, Ala194
CB-CB-PES	FB	-6.5	Val74^a^, Arg A:50^a^, Lys58^a^, Arg D:50^b^, Asp61^b^, Lys29^b^, Asp69^c^, Ser D:47^c^	Val 74, Cys B:76, Ser A:47, Cys E:76, Cys D:28, Ala27, Ala59, Cys A:28, Leu66, Leu72, Pro70, Gly73, Ser31
CB-CB-PES	TR	-6.3	Tyr45^a^, Arg327^a^, Asp163^a^, Asp69^b^, Glu147^c^, Lys144^c^, Arg50^d^, Glu147^e^	Gly164, Cys161, Ala162, Cys179, Ser44, Leu72, Gly123, Leu46, Leu122, Trp128
SB-SB-PES	HSA	-8.4	Ser193^a^, Arg114^a^, His146^a^, Leu115^a^, Lys190^b^, Glu425^b^, Arg197^c^, Asp108^c^, Arg145^c^, Arg117^c^, Glu141^c^, Arg428^c^, Lys432^e^, Arg186^f^	Asn429, Val424, Ile523, Tyr138, Met123, Leu463, Gln45, Ala194, Ile142, Leu112, Asn111, Pro110, Asn109, Pro147, Tyr148
SB-SB-PES	FB	-6.5	Lys29^a^, Ala27^a^, Ser31^a^, Asp69^c^, Arg50^c^, Asp71^d^, Lys58^d^, Cys28^f^, Asp61^g^	Ser47, Gly73, Leu66, Cys49, Trp33, Pro60, Ala59, Pro70, Ala68, His67
SB-SB-PES	TR	-6.8	Arg50^a^, Glu328^c^, Arg327^c^, Arg324^c^, Asp69^e^, Glu147^g^, Asp163^g^	Ala70, Ile49, Asn75, Leu72, Leu122, Tyr45, Leu46, Gly123, Trp128, Ile123, Pro145, Phe167, Ala162, Leu146, Cys161
PB-PB-PES	HSA	-7.9	Asn109^a^, Arg145^a^, Ser193^a^, Lys190^a^, Arg186^a^, Arg186^b^, Lys190^b^, His146^b^, Arg117^c^, Asp108^c^, Glu425^c^, Glu520^c^, Arg428^d^, Lys519^d^, Arg186^d^, Arg114^e^, Arg197^f^	Ile523, Val424, Pro421, Leu112, Pro110, Leu115, Tyr138, Ile142, Ala194, Gln459, Val462
PB-PB-PES	FB	-6.3	Ala59^a^, Lys58^a^, Val74^a^, Arg A:50^a^, Asp61^b^, Asp61^c^, Asp69^c^, Lys58^d^, Arg A:50^e^, Arg D:50^e^, Lys29^e^	Cys28, Trp33, Ser47, Ser31, Leu66, Leu72, Gly73, Pro70, Cys28, Ser47, Cys49, Ala27
PB-PB-PES	TR	-6.1	Tyr68^a^, Arg327^a^, Arg50^a^, Arg50^b^, Arg327^b^, Lys144^c^, Arg324^c^, Glu147^c^, Arg50^d^	Leu146, Trp128, Gly123, Ile323, Leu72, Cys161, Ala73, Leu122, Tyr45, Ley46, Pro74, Asn75
CB-CB-CB-PES	HSA	-8.3	Arg145^a^, Lys190^a^, Asn109^a^, Arg114^b^, Arg186^b^, Glu141^c^, Glu520^c^, Glu425^c^, Asn108^c^, Arg186^d^, Arg428^d^, Arg117^e^, Arg197^f^, His146^h^	Pro425, Leu122, Ile523, Tyr138, Asn111, Pro110, Leu463, Val462, Gln459, Ser193, Ala194, Ile142, Leu115
CB-CB-CB-PES	FB	-6.5	Ser31^a^, Ala59^a^, Trp33^a^, Arg A:50^a^, Arg D:50^c^, Asp A:30^c^, Asp D:30^d^, Asp61^d^, Lys29^d^, Lys58^d^, Asp61^e^, Ala27^e^, Lys29^e^, Lys58^e^, Cys76^f^, Cys49^f^, Cys D:28^h^, Cys A:28^h^	Pro70, Leu66, Ala68, Phe35, Cys76, Trp33, Cys49, Ser47, Gly73
CB-CB-CB-PES	TR	-6.3	Ser44^a^, Ala162^a^, Trp128^a^, Glu147^b^, Arg327^b^, Asp166^c^, Asp163^c^, Arg50^c^, Asp69^e^	Ile323, Leu326, Asn129, Gly123, Leu122, Leu146, Pro145, Phe167, Tyr45, Leu46, Leu72, Cys161, Cys179, Gly164, Pro74, Ala73, Leu72
SB-SB-SB-PES	HSA	-8.7	Gln459^a^, His146^a^, Arg186^a^, Lya190^a^, Leu115^a^, Arg114^a^, Arg186^b^, Lys190^b^, Glu141^c^, Arg145^c^, Arg197^c^, Asp108^c^, Arg117^d^, Arg186^d^, Glu520^e^, Arg428^e^, Lys432^e^, Lys519^e^, Arg114^e^, Glu425^e^	Pro421, Asn109, Pro110, Leu463, Val462, Ala194, Ser193, Ile523, Val424, Asn429, Ile142, Met123, Tyr138, Tyr161 Val116
SB-SB-SB-PES	FB	-6.3	Ser47^a^, Ser31^a^, Lys58^b^, Asp69^c^, Asp61^c^, Lys29^c^, Arg A:50^d^, Asp71^e^, Arg D:50^e^	Cys28, Gly73, Val74, Ser47, Ala27, Leu72, Pro70, Ala68, Cys76, Pro60, Phe35, Trp33, Leu66, Pro34
SB-SB-SB-PES	TR	-6.6	Gly164^a^, Tyr45^a^, Gly123^a^, Trp128^a^, Lys144^a^, Arg327^a^, Arg50^a^, Asp69^c^, Asp163^c^, Arg327^d^, Arg50^d^, Lys144^e^, Arg50^e^, Gly147^e^	Ala162, Ser44, Cys161, Phe167, Leu122, Leu46, Cys179, Leu146, Leu326, Leu72, Asn75, Ala73
PB-PB-PB-PES	HSA	-8.1	Arg117^a^, Arg186^a^, Arg114^a^, His146^a^, Ser193^a^, Glu520^b^, Arg186^b^, Arg117^b^, Asp183^c^, Arg197^c^, Lys190^c^, Arg145^c^, Glu425^c^, Arg117^d^, Lys519^e^, Asp108^e^	Pro147, Leu463, Ser517, Tyr148, Gln459, Val462, Pro421, Ala194, Pro110, Asn109, Leu115, Ile142, Val116, Ile142, Leu182, Leu112, Tyr138, Phe185, Met123, Tyr161
PB-PB-PB-PES	FB	-6.8	Ser31^a^, Lys29^a^, Val74^a^, Arg D:50^a^, Ser47^a^, Asp E:61^b^, Lys E:58^c^, Asp69^c^, Asp B:61^c^, Lys B:58^d^, Arg A:50^d^, Arg D:50^e^, Arg A:50^e^, Lys B:58^e^	Pro70, His67, Leu66, Leu72, Gly73, Ala27, Trp33, Lys29, Lys58, Cys A:28, Cys D:28, Ala27, Ala59, Cys D:49, Cys A:49, Ala68, Phe35, Cys A:49, Cys D:49, Cys 76, Trp33, Ser47
PB-PB-PB-PES	TR	-6.2	Lys291^a^, Lys276^a^, Ser286^a^, Asp277^a^, His300^a^, Gln92^a^, Asp277^b^, His300^b^, Asp297^c^, Lys239^d^, His207^e^, Asp90^e^, Lys88^e^, Glu212^g^	Ser287, Phe211, Ser208, Phe285, Tyr238, Gln283, Gly88, Ser87

a: Conventional Hydrogen Bond

b: Salt Bridge c: Weak Van der Waals.

d: Unfavourable Positive-Positive e: Attractive Charge.

f: Pi-Alkyl g: Pi-Anion, Pi-Cation h: Pi-Sulfur.

The three-dimensional conformations, electrostatic interaction profiles, and three-dimensional interaction diagrams for the docking processes of CB2-PES, SB2-PES, and PB2-PES are delineated in Supplementary Materials Fig. S8, Fig. S9, and Fig. S10, respectively. Similarly, the representations for CB3-PES, SB3-PES, and PB3-PES are elucidated in Supplementary Materials Fig. S11, Fig. S12, and Fig. S13.

This study focused on zwitterionic small molecules and their interactions with PES, using molecular docking to provide initial insights into fundamental interaction mechanisms. However, we acknowledge that in practical applications, PES membranes consist of polymer chains, which introduces additional complexity not captured in our current simulations. Simulating an entire PES membrane, with its high molecular weight and intricate polymer network, is challenging and was beyond the scope of this study. Future research will need to explore the effects of zwitterionic materials when incorporated into polymer matrices. Investigating the antifouling effectiveness of zwitterionic polymers with different chain lengths and their interactions with proteins will offer a more comprehensive understanding of membrane performance and fouling reduction in practical applications. This will provide valuable information for optimizing zwitterionic modifications to enhance membrane functionality and biocompatibility.

### Diblock ZW-PES membrane models interactions with human serum proteins

**Fig. 8:**
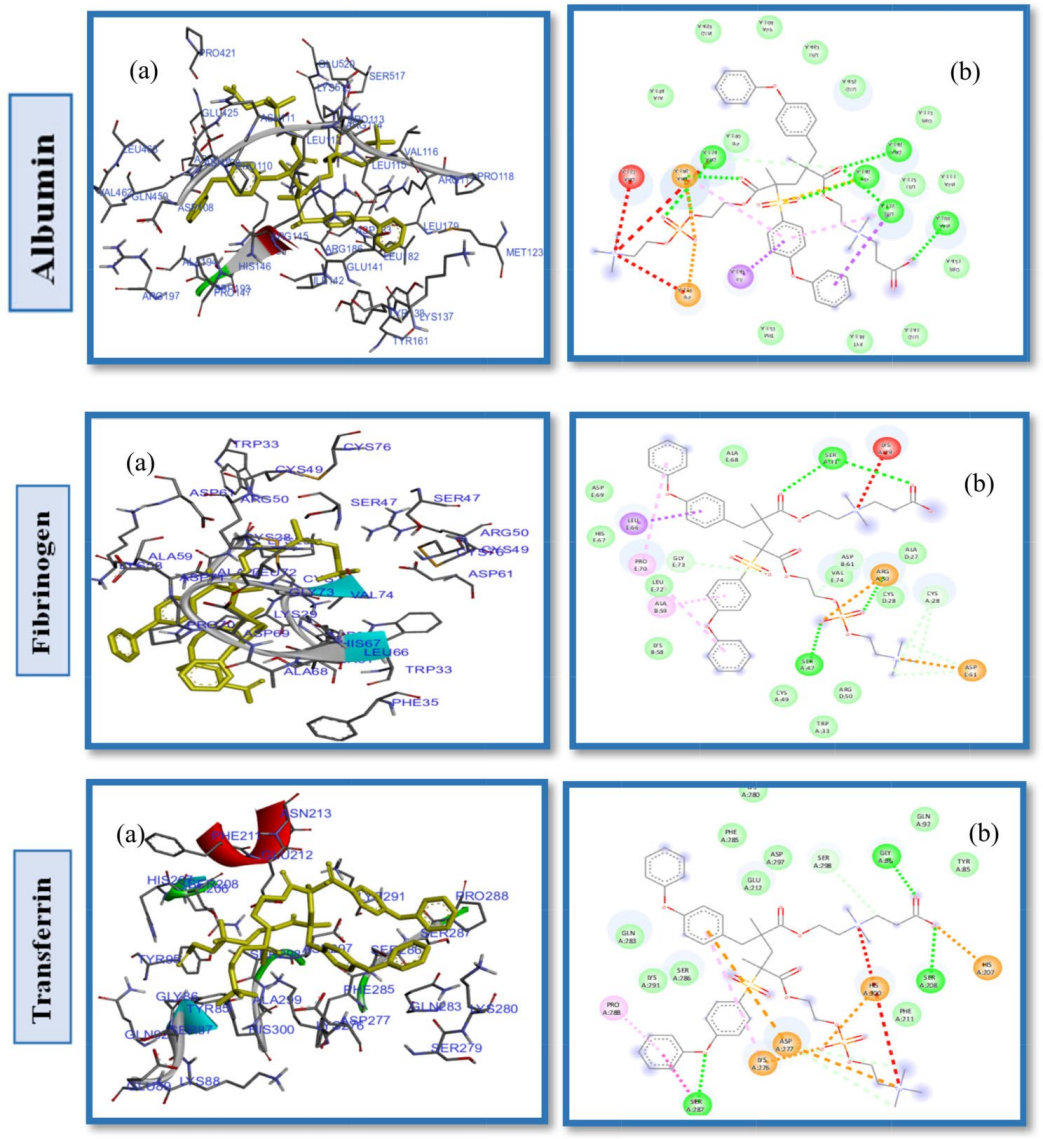
(**a**) 2D interaction diagrams, and (**b**) Electrostatic interaction profiles for the docking of CB-PB-PES (1 to 1 ratio) with HSA, FB and TR

In to provide in dept insight into the fundamental knowledge of the influence of zwitterionic pendant groups, their intermolecular interactions, and the related fouling/antifouling behaviours, our work was designed to use integrated ZW-PES models. We initially studied a 1:1 ratio for each ZW combination (CB-PB-PES, SB-PB-PES, and CB-SB-PES), and subsequently increased it to 1:2 and 2:1 of each. Table 4 shows that CB-PB-PES binds to the active sites of HSA, FB, and TR with affinity binding of -8.4, -6.3, and − 6.3 kcal mol^− 1^, respectively. This is the least favourable combination when compared to the other two groups. Like as other ligands, CB-PB-PES also bind to polar sites of HSA with higher affinity binding interaction than FB and TR. The amino acids: Leu115, His146, Asn109 and Arg145 were engaged in conventional hydrogen bonding between CB-PB-PES ligand and HSA, as presented in Fig. 8; Table 4. Furthermore, the Arg186, Lys519, Arg117, Glu425, Asp108, Lys190 and Glu141 interact in a hydrophilic manner with this ligand On the other hand, CB-PB-PES had non-polar interactions with HSA via Tyr138, Met123, Pro421, Ile425, Leu112, Asn111, Pro113, Leu463, Gln459 and ala194 residues as could be seen in Fig. 8. CB-PB-PES binds to the active sites of FB and TR with a binding affinity of -6.3 kcal mol^− 1^ and unique interaction patterns that are presented in Table 4; Fig. 8 (Additional interaction analyses are presented in Supplementary Material Fig. S14). Despite the fact that we observe various interaction patterns (Table 4), this ligand does not show any improvement in the binding energies when compared to CB*2-PES and PB*2-PES. The results showed that PB*2-PES performed superiorly to CB-PB-PES with all tested proteins. Repulsive forces acting on HSA, FB and TR was shown in a decreasing order of CB*2-PES > CB-PB-PES > PB*2-PES, consistent with the affinity binding energies.

**Fig. 9:**
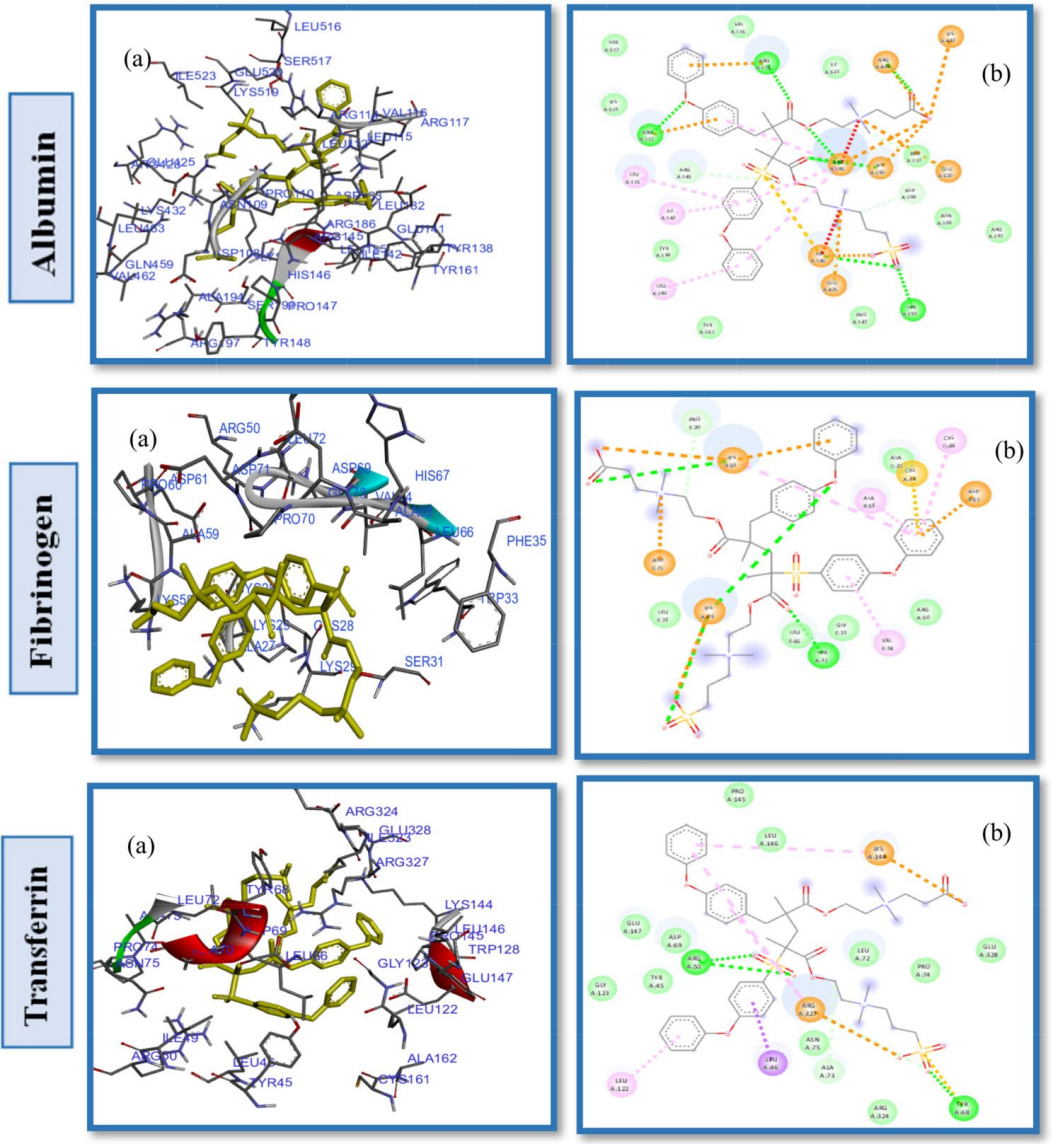
(**a**) 2D interaction diagrams, and (**b**) Electrostatic interaction profiles for the docking of CB-SB-PES (1 to1 ratio) with HSA, FB and TR

**Fig. 10:**
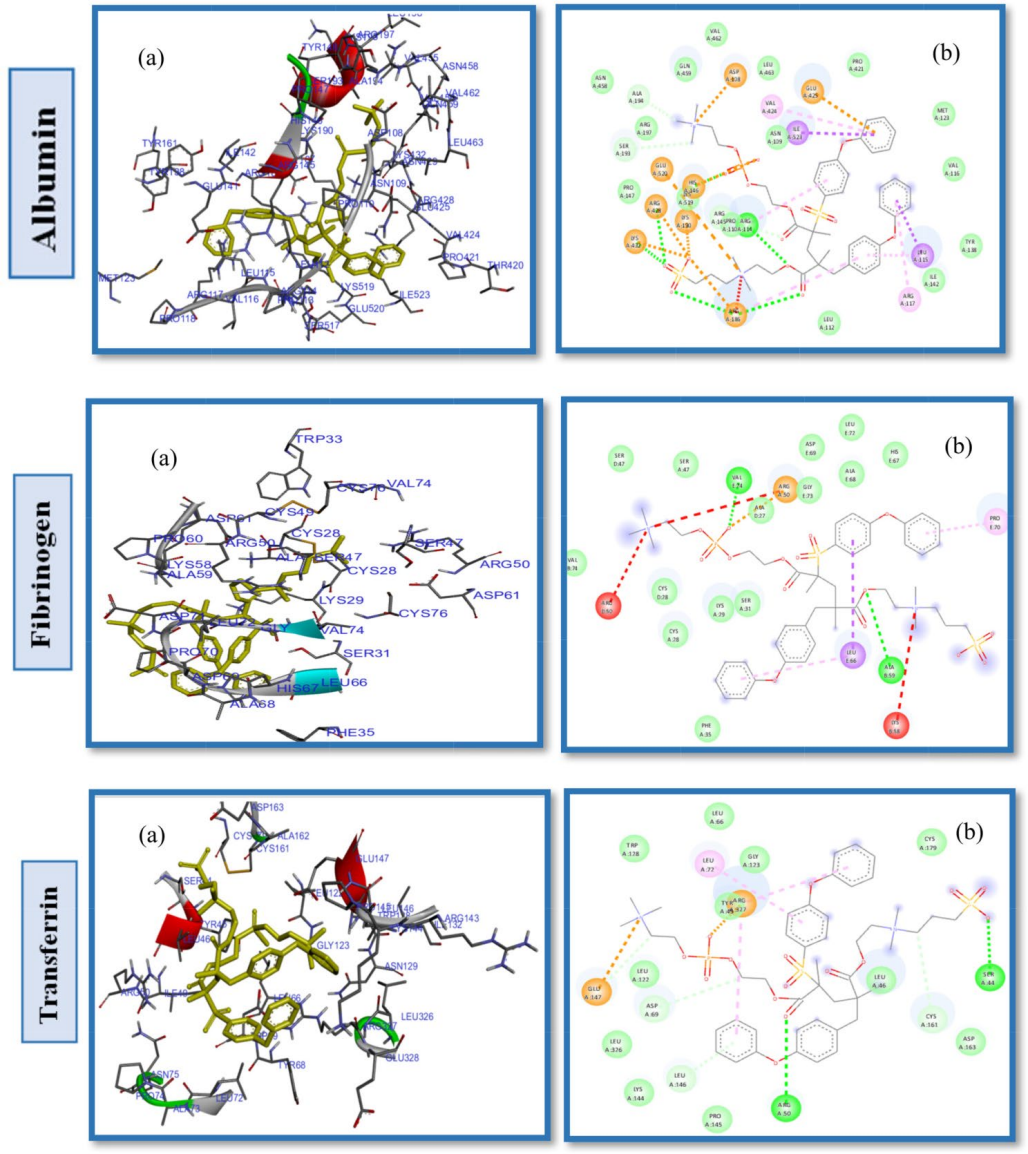
(**a**) 2D interaction diagrams, and (**b**) Binding energy outcomes and receptor interactions of SB-PB-PES (1:1 ratio) with HSA, FB and TR

Illustrated in Fig. 9, we conducted docking simulations of CB-SB-PES with Human Serum Albumin (HSA) to evaluate the influence of differing pendant groups within ZW chains on the hemocompatibility of PES. These simulations revealed that the amino acids forming the hydrophobic pocket are Pro147, Tyr161, Leu182, Tyr138, Asn109, Ile142, Leu115, Pro110, Ser517, Val116, and ILe523. Further analysis, as depicted in Fig. 9; Table 4, showed the zwitterionic ligand’s binding to HSA was facilitated through hydrophilic interactions with amino acids such as Arg114, Arg117, Ser193, Arg428, Arg186, Lys190, His146, Glu427, Glu520, Lys432, Arg145, Asp108, Arg197, and Lys519 (Fig. S15. Supplementary Material). The interaction between the CB-SB-PES model and HSA demonstrated a binding energy of -8.3 kcal/mol, similar to CB2-PES and SB2-PES, which showed − 8.4 kcal/mol. Interestingly, when compared to CB2-PES and SB2-PES, the binding energies for CB-SB-PES interactions with FB & TR proteins were significantly lower, at -5.5 kcal/mol and − 6 kcal/mol respectively, suggesting that surface modifications with various pendant groups could enhance PES membranes’ hemocompatibility. The order of affinity energy and antifouling capabilities among HSA, FB, and TR proteins towards these ligands was observed as SB2-PES > CB2-PES > CB-SB-PES, with Table 5; Fig. 10 summarizing these docking interactions and affinity energies.

Furthermore, the docking of SB-PB-PES into the HSA active site also identified both hydrophobic and hydrophilic interactions between the protein and ligand, including hydrogen bonds and attractive charges involving Arg114, Lys432, Asp108, His146, Lys190, and Glu520 (Fig. S16. Supplementary Material). Hydrophobic contacts were anticipated with amino acids such as Asn458, Ala194, and others, as shown in Fig. 10. The binding energies for SB-PB-PES were notably lower than those for SB-SB-PES across all proteins tested, indicating a superior hemocompatibility of this diblock polymer compared to modifications using SB or PB alone. This suggests that the combination of sulfobetaine and phosphobetaine in SB-PB-PES significantly enhances its fouling resistance, likely due to a complex interplay of factors including molecular structure, functional groups, and zwitterionic properties, rather than a singular structural or chemical element. Minor changes in the zwitterionic structure can markedly affect interactions between polymers and water, underscoring the nuanced impact of zwitterionic surface modifications on antifouling performance. These interactions may have an impact on polymer conformation and flexibility, which might then affect the binding abilities and antifouling characteristics of polymers. In this case, using 2 different ZWs in PES surface modification, creates relatively complex interaction patterns. Certain amino acids participate in two or more different interactions. Numerous interactions, as well as interactions that overlap with particular amino acids, may result in stronger or weaker polymer binding interactions, which have a substantial impact on the effectiveness of membranes. All the images of protein-ligand interactions and 2D diagrams (Figs. 8, 9 and 10) clearly show that the use of various ZWs in PES modification leads to a variety of electrostatic interactions affecting hydrogen shell formation around zwitterions, which give polymers unpredictable behaviour.

In the final section again the amount of ZWs on the PES was raised to investigate how packing density affected membrane hemocompatibility. Despite our previous findings that increasing ZWs from 2 to 3 mol did not have much effect, we investigated it again with different ZWs. So, six categories of ZW modified PES in 1:2 and 2:1 combination ratios were designed using Chemdraw to prepare them for docking. Docking studies between all examined proteins (HSA, FB, and TR) and CB-PB-PES, SB-PB-PES, and CB-SB-PES (1 : 2 ratio combination of ZWs) as ligands reveal that increasing ZWs value does not improve PES hemocompatibility. Affinity energy between HSA, FB and TR proteins and these ligands are ranked as CB-PB-PES > CB-SB-PES > SB-PB-PES which is similar to 1:1 ratio combination. Docking simulations were also conducted between the aforementioned proteins and the ZW combinations of CB-PB-PES, SB-PB-PES, and CB-SB-PES (2:1 ratio). The results showed no improvement in the affinity energies compared to the 1:1 combination ratio, indicating that increasing packing density is not always effective. However, the resistance characteristics of these ligands are completely different from those of the previous ones and rank in decreasing order SB-PB-PES > CB-PB-PES > CB-SB-PES, supporting the unpredictable behaviour of modified polymers with different ZWs. All of the ligand affinity energies are listed in Table 5.

**Table 4:** Binding energy outcomes and receptor interactions of HSA, FB, and TR interactions with CB-PB-PES, SB-PB-PES, and CB-SB-PES

Ligand	Protein	Binding energy (kcal/mol)	Receptor contacts Hydrophilic Hydrophobic	
CB-PB-PES	HSA	-8.4	Leu115^a^, His146^a^, Asn109^a^, Arg145^a^, Arg186^c^, Lys519^c^, Glu425^d^, Asp108^d^ Lys190^d^, Glu141^d^, Arg117^e^	Tyr138, Met123, Pro421, Ile425, Leu112, Asn111, Pro113, Leu463, Gln459, Ala194
CB-PB-PES	FB	-6.3	Ser47^a^, Arg50^a^, Ser31^a^, Arg50^c^, Asp61^c^, Asp69^c^, His47^c^, Lys58^c^, Lys29^d^, Asp61^e^, Arg50^e^	Trp33, Cys49, Cys28, Ala59, Leu72, Gly73, Pro70, Ala27, Leu66, Ala68
CB-PB-PES	TR	-6.3	Gly86^a^, Ser208^a^, Ser287^a^, Asp227^b^, Lys276^b^, Lys291^c^, Glu212^c^, Asp297^c^, Lys208^c^, His300^d^, His207^e^, His300^e^	Phe285, Ser298, Gln92, Tyr85, Phe211, Ser286, Gln283, Pro288
CB-SB-PES	HSA	-8.3	Arg114^a^, Arg117^a^, Ser193^a^, Arg428^a^, Arg186^a^, Lys190^a^, His146^a^, Arg186^b^, Lys190^b^, His146^b^, Arg145^c^, Asp108^c^, Arg197^c^, Lys519^c^, Arg186^d^, His146^d^, Glu427^e^, Glu520^e^, Arg428^e^, Lys432^e^, Arg114^g^, Arg117^g^	Pro147, Tyr161, Leu182, Tyr138, Asn109, Ile142, Leu115, Pro110, Ser517, Val116, Ile523
CB-SB-PES	FB	-5.5	Lys58^a^, Lys29^a^, Ser31^a^, Lys29^b^, Lys58^b^, Arg50^c^, Cys28^c^, Asp71^e^, Asp61^g^	Val74, Gly73, Leu66, Leu72, Ala59, Ala27, Pro70
CB-SB-PES	TR	-6	Arg50^a^, Tyr68^a^, Arg327^b^, Lys144^b^, Glu147^c^, Asp69^c^, Glu328^c^, Arg324^c^	Ala73, Asn75, Leu46, Leu122, Tyr45, Gly123, Pro74, Leu72, Leu146, Pro146
SB-PB-PES	HSA	-8	Arg114^a^, Arg186^a^, Arg428^a^, Lys432^a^, Lys519^a^, Arg428^b^, Arg186^b^, Lys432^b^, Arg117^c^, Arg145^c^, Arg197^c^, Arg186^d^, Asp108^e^, Lys190^e^, Glu520^e^, His146^e^, Glu425^g^	Asn458, Ala194, Gln459, Val462, Ser193, Leu463, Val424, Asn109, Ile523, Pro421, Met123, Val116, Pro147, Pro110, Tyr138, Leu115
SB-PB-PES	FB	-5.4	Ala59^a^, Val E:74^a^, Asp69^c^, Lys29^c^, Lys58^d^, Arg D:50^d^, Arg A:50^d^, Arg A:50^e^	Phe35, Pro70, Ser31, Cys D:28, Cys A:28, Val B:74, Ser47, Ala27, Gly73, Ala68, Leu72, His76
SB-PB-PES	TR	-5.5	Arg50a, Ser44a, Glu147e, Arg327e, Asp169c, Lys144c, Asp163c	Leu46, Cys161, Pro145, Leu146, Leu326, Leu122, Tyr45, Leu72, Trp128, Leu72, Leu66, Cys179

a: Conventional Hydrogen Bond

b: Salt Bridge c: Weak Van der Waals.

d: Unfavourable Positive-Positive e: Attractive Charge.

f: Pi-Alkyl g: Pi-Anion, Pi-Cation.

**Table 5:** Binding energy outcomes and receptor interactions of HSA, FB, and TR with ZW-PES modified at different zwitterionic ratios

Ligand	Protein	Binding energy (kcal/mol)	
		1:2 ratio	2:1 ratio
CB-PB-PES	HSA	-8.4	-8.6
CB-PB-PES	FB	-6.3	-6.5
CB-PB-PES	TR	-6.5	-6.5
CB-SB-PES	HSA	-8.3	-8.3
CB-SB-PES	FB	-5.8	-6.2
CB-SB-PES	TR	-6.1	-6.4
SB-PB-PES	HSA	-8	-9.1
SB-PB-PES	FB	-5.9	-7.3
SB-PB-PES	TR	-6	-6.4

## Conclusion

Hemodialysis (HD) membranes and other biomedical equipment are susceptible to biofouling, leading to substantial health concerns and financial losses. Zwitterions, with both positive and negative charge groups, interact strongly and electrostatically with water molecules, creating a solvation shell on their surfaces. This shell serves as a physical and energy barrier, reducing fouling by preventing foulants from adhering to the surface. Pendant groups significantly influence the performance of zwitterions due to their role in electrostatic interactions and hydrogen shell formation. Therefore, even minor alterations in these areas and charge distributions can result in notable modifications in membrane hemocompatibility, thereby increasing hemodialysis therapy efficiency. Given that medical experiments are costly and time-consuming, simulations could be a valuable method to predict outcomes and reduce research costs. Competent users would need less than 2–3 months to complete a new concept design using the proposed simulations, which is significantly cheaper than building a model for commercial testing to achieve the same level of detail regarding fouling resistance and hemocompatibility.

In this study, molecular docking was utilized to examine the interactions between similar zwitterionic structures with different pendant groups (CBMA, SBMA, and MPC), both alone and in conjunction with PES, as ligands and the binding sites of the proteins (HSA, FB, TR) to determine the optimal ligand for use in HD therapy. Docking simulation was extended across various combinations of zwitterions (CB-PB-PES, CB-SB-PES, and SB-PB-PES in 1:1, 1:2, and 2:1 ratios) as ligands and the mentioned proteins to assess the impact of diblock polymers and grafting density on membrane fouling performance. The results showed that phosphobetaine exhibited lower affinity energies than other zwitterions for all examined proteins, whether used alone or in conjunction with PES. A decreasing order of energy affinity and fouling resistance was observed among the proteins and zwitterions as SBMA > CBMA > MPC, consistent with the trend seen in docking ZW-PES with proteins, ranked as SB-PES > CB-PES > PB-PES, aligning with their surface hydration. A similar pattern was observed when the number of ZWs on the PES was doubled, with PES modification with PB producing the best results. This significant decrease in binding affinity highlights the critical role of ZW grafting density in antifouling.

To investigate the effect of packing density, the number of ZWs was increased again by a factor of 3. The resulting affinity energy did not follow a clear pattern as seen in earlier situations, especially in docking with FB. The presence of multiple charge components in a ligand and overlapping electrostatic interactions can influence hydrophilic bonds and lead to unpredictable patterns. Furthermore, the affinity energies showed that increasing zwitterions from 2 to 3 does not improve fouling resistance, indicating that occasionally increasing packing density can lead to increased roughness, which is not a positive factor and might result in heightened hemoincompatibility.

A docking study was also conducted on diblock-PES polymers. When a 1:1 combination ratio of different ZWs was used in PES modification, the results were often superior to using modified PES with identical ZW blocks. The findings indicate that both pendant groups (the nature of the anionic groups) and grafting density significantly impact antifouling performance, leading to an antifouling ranking of SB-PB-PES > CB-SB-PES > CB-PB-PES. It should be noted that a variety of factors influence how well ZWs resist fouling. Any minor change to the pendant group, a charge component in ZW structures responsible for electrostatic interactions, can have a major impact on affinity energy and fouling properties. The existence of different negative parts in a ligand leads to complex interaction patterns that make the ligand’s behavior unpredictable. Certain amino acids can engage in multiple interactions, potentially strengthening or weakening ligand hydrophilic bonds and significantly affecting affinity binding energy. Figures 8 and 9, and 10 illustrate that the use of various ZWs results in complex patterns with varying electrostatic interactions, affecting binding energy and fouling resistance. It should be mentioned that in diblock polymers, increasing ZW combination ratios to 1:2 or 2:1 did not yield better results than a 1:1 ratio, repeatedly demonstrating that grafting density is effective up to a certain level, beyond which it can lead to increased roughness, acting as a negative factor in fouling resistance.

Overall, compared to the time-consuming and energy-intensive trial-and-error experiments, the resulting affinity energy through computational docking provides valuable information regarding membrane hemocompatibility and fouling resistance. Based on these findings and our extensive research on PES membranes used in hemodialysis, it is anticipated that combining SBMA and MPC will significantly enhance the effectiveness of HD and PES membranes. This will be considered in our upcoming experimental and clinical studies.

## Electronic supplementary material

Below is the link to the electronic supplementary material.

Supplementary Material 1

## Data Availability

The raw/processed data supported the key findings of this study are available from the corresponding author [A. Abdelrasoul] on reasonable request.
