# Supplementary material for: Simulation-based assessment of zwitterionic pendant group variations on the hemocompatibility of polyethersulfone membranes
Source: Funct Compos Mater. 2024 Sep 11;5(1):12. doi: 10.1186/s42252-024-00062-6 (PMC11412084; doi:10.1186/s42252-024-00062-6)
Supplement: Supplementary file 1 — Supplementary Material 1 [file 42252_2024_62_MOESM1_ESM.docx]

**Assessing the Impact of Zwitterionic Pendant Group Variations on the Hemocompatibility of Polyethersulfone Membranes**

***Simin Nazari^1^ and Amira Abdelrasoul^1,2*^***

*^1^Division of Biomedical Engineering, University of Saskatchewan, 57 Campus Drive, Saskatoon, Saskatchewan, S7N 5A9, Canada.*

*^2^Department of Chemical and Biological Engineering, University of Saskatchewan, 57 Campus Drive, Saskatoon, Saskatchewan, S7N 5A9, Canada.*

****Corresponding Author: amira.abdelrasoul@usask.ca, Tel: (306) 966 2946, Fax: (306) 966 4777***

**Supplementary Materials**

**
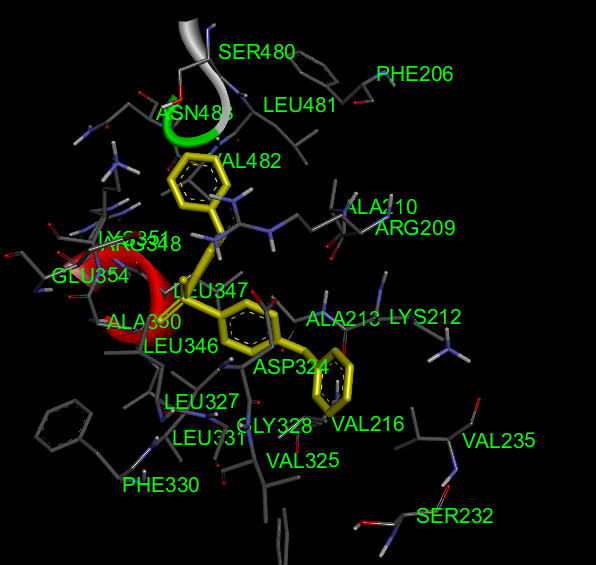
PES-HSA**

**PES-FB**

**
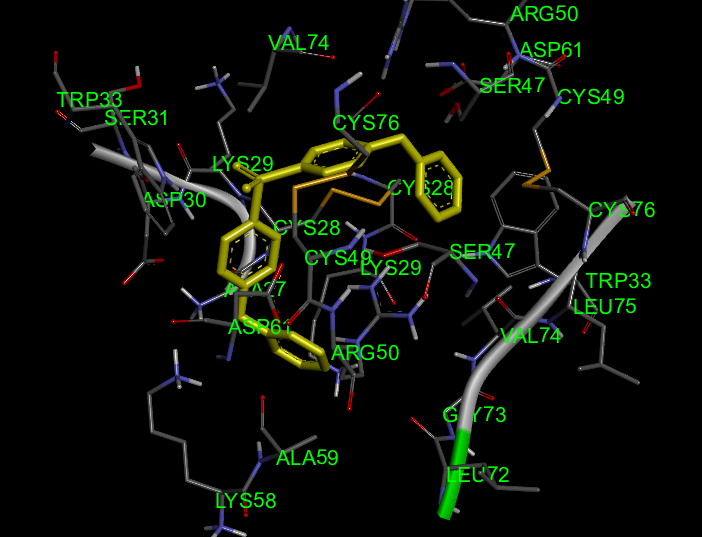
**

**
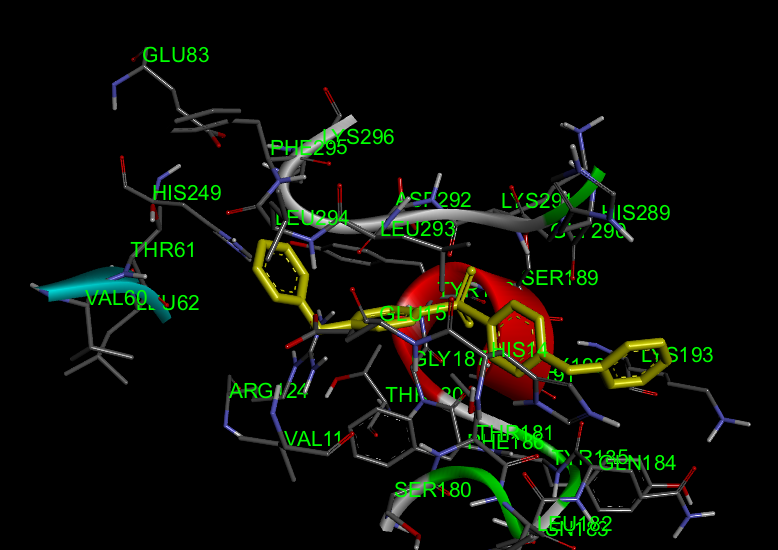
PES-TR**

**S1.** 3D interaction diagrams for the docking of PES with HSA, FB and TR

**
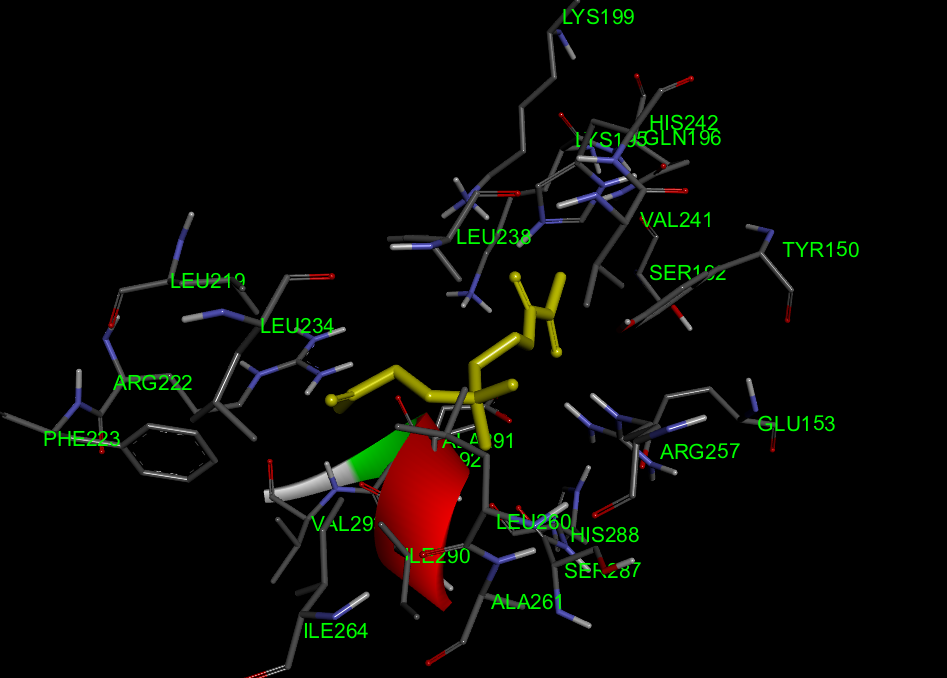
CB-HSA**

**
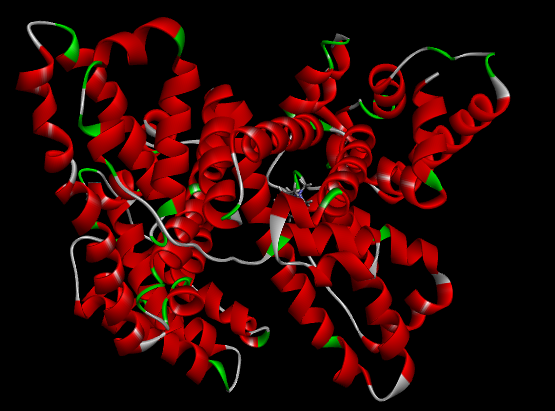
**

**
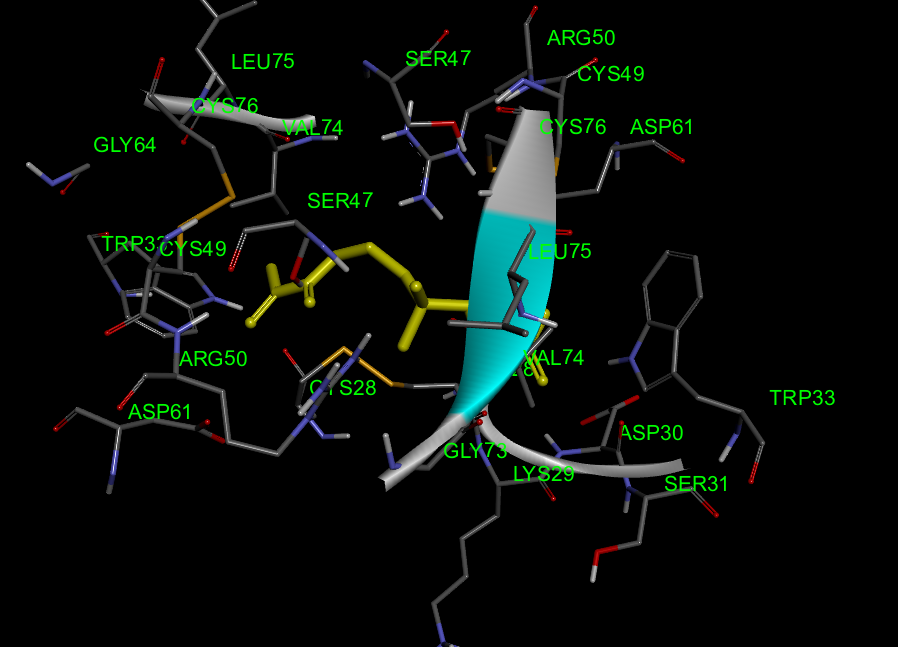
CB-FB**

**
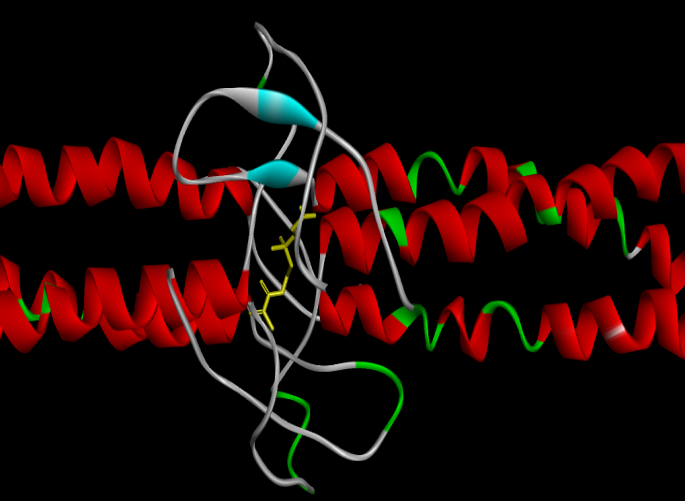
**

**
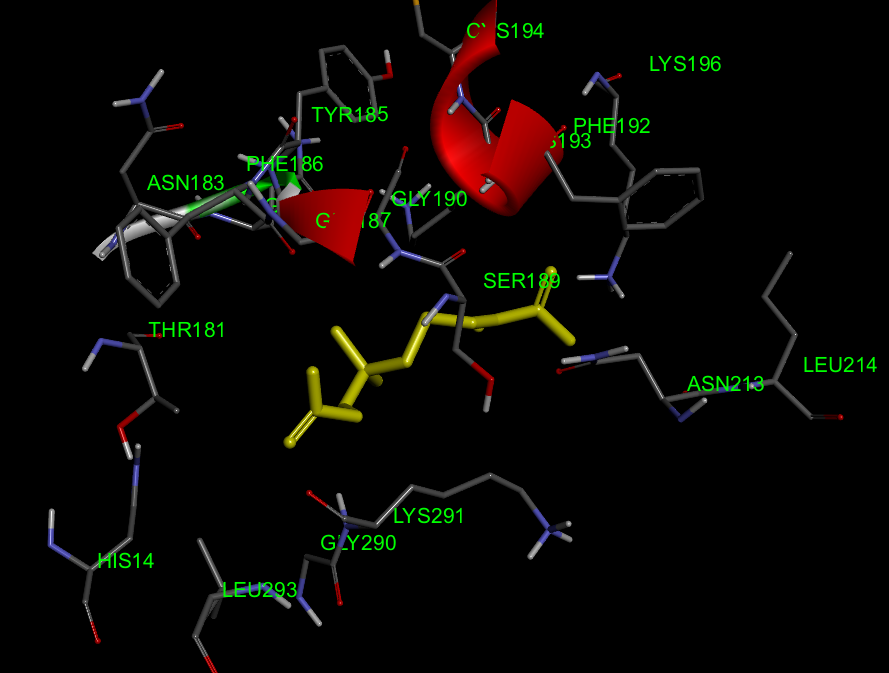
CB-TR**

**
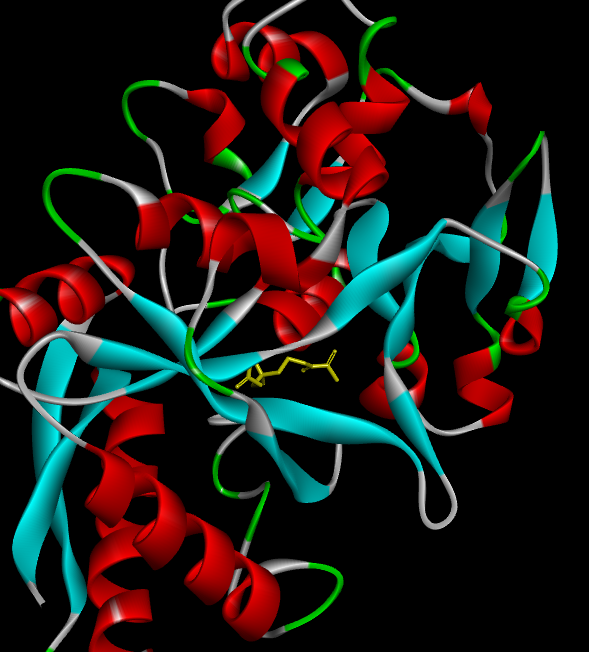
**

**S2.** 3D conformation and interaction diagrams for the docking of CB with HSA, FB and TR

**
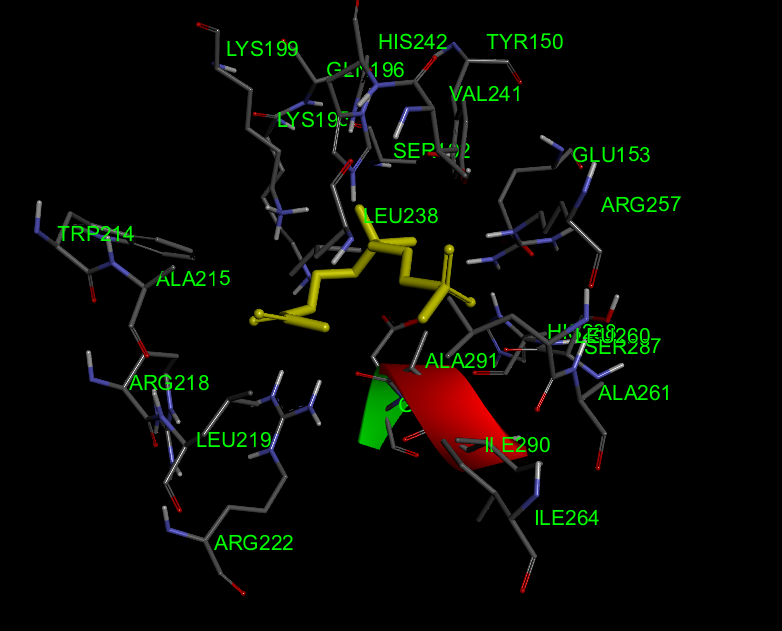
SB-HSA**

**
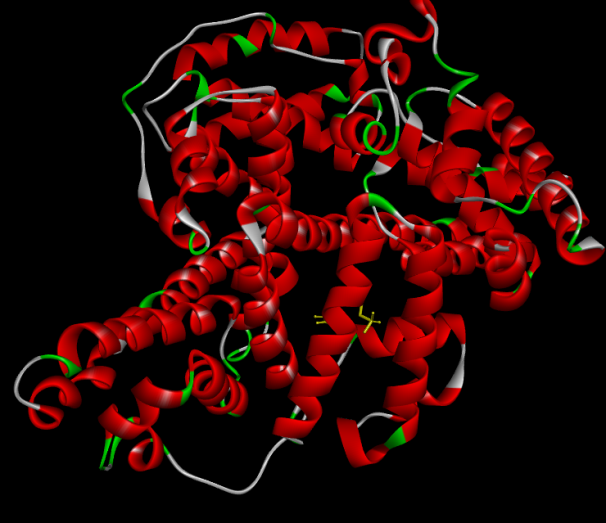
**

**
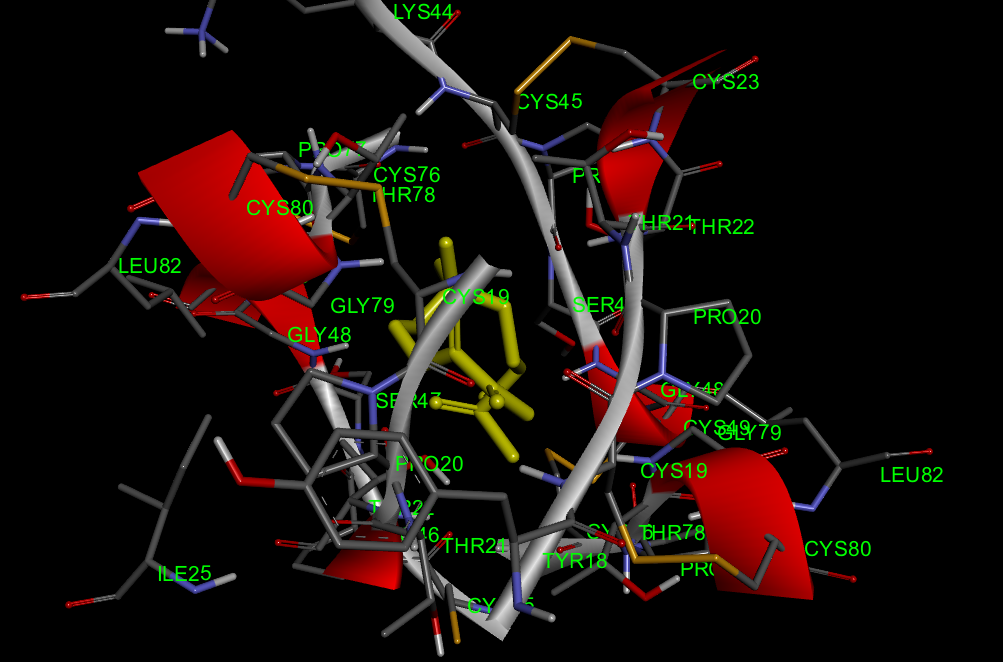

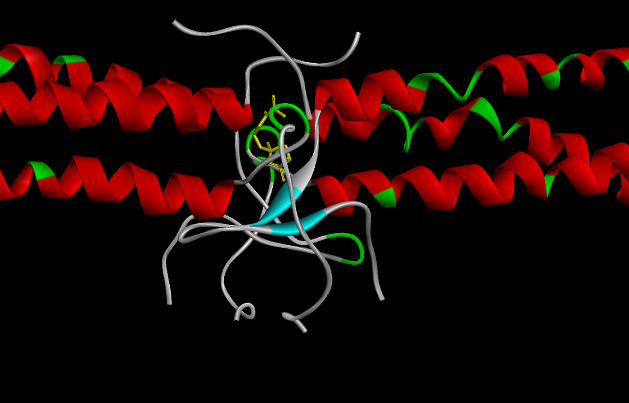
SB-FB**

**
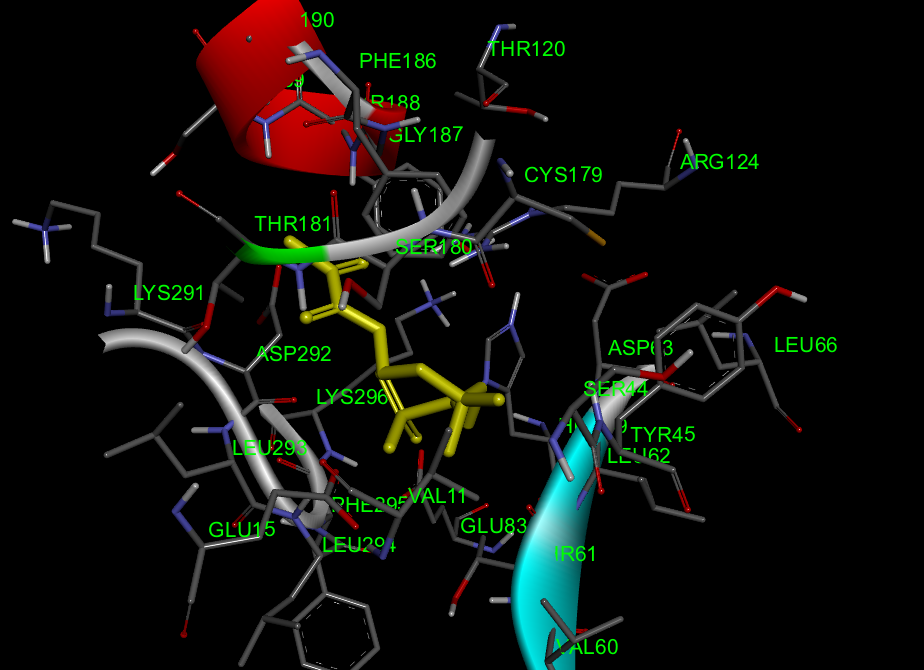

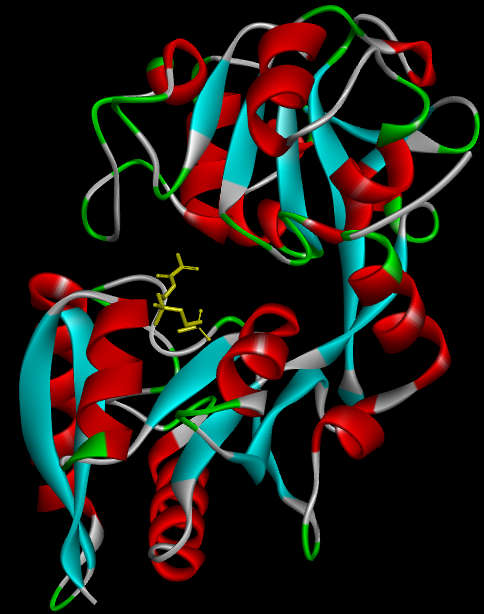
SB-TR**

**S3.** 3D conformation and interaction diagrams for the docking of SB with HSA, FB and TR

**PB-HSA**

**
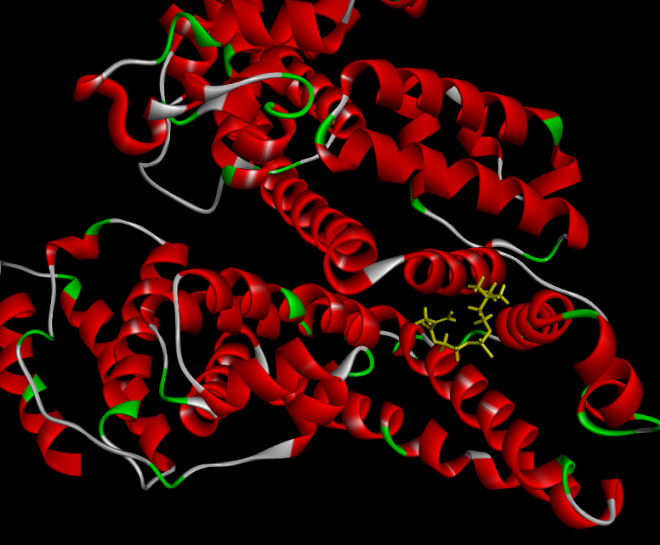

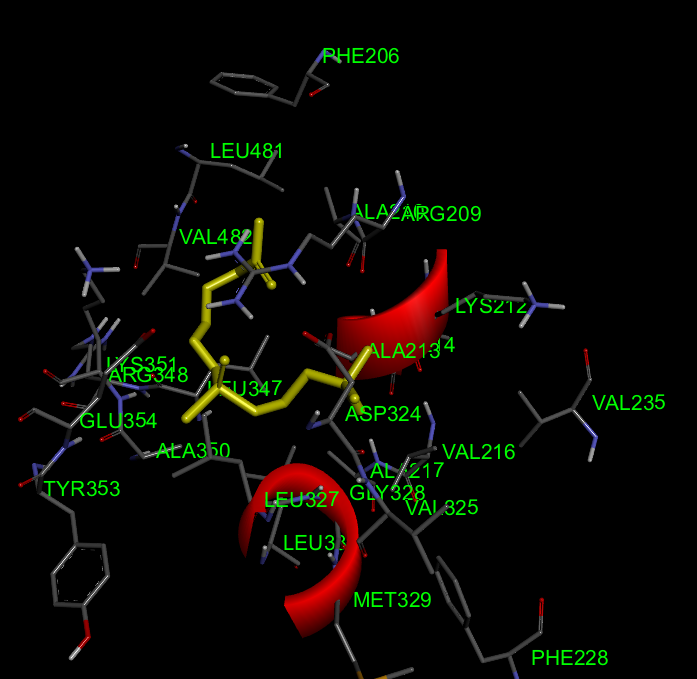
**

**
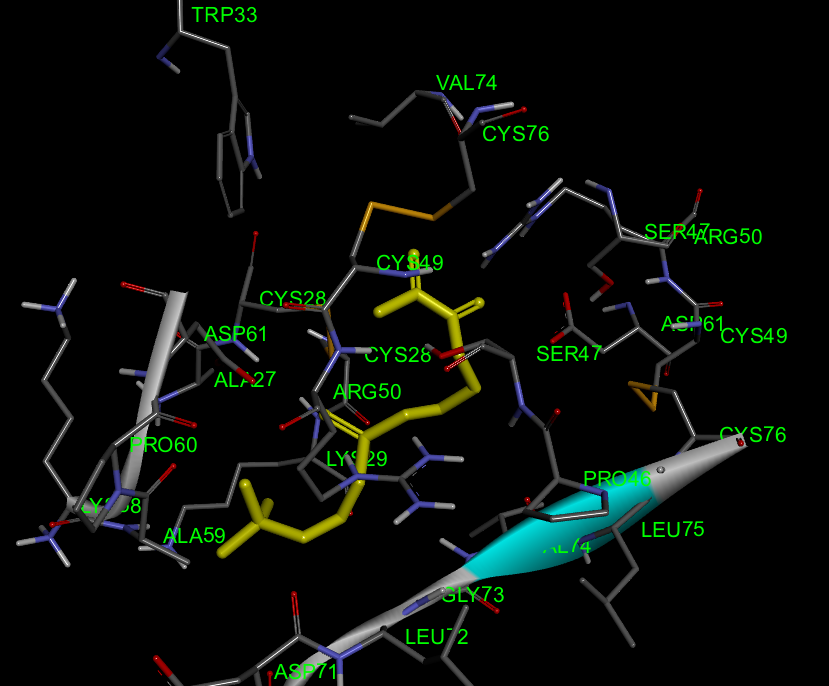
PB-FB**

**
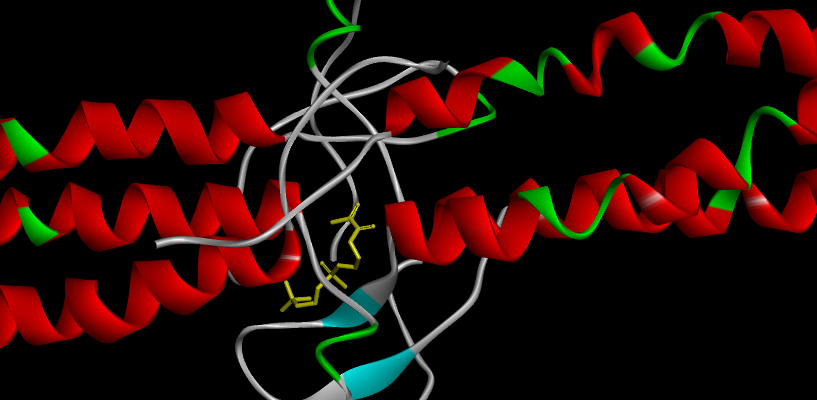
**

**
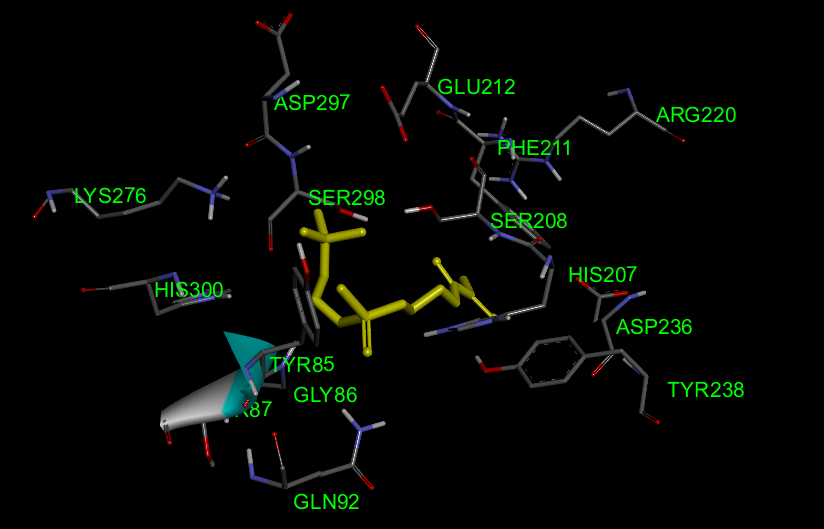

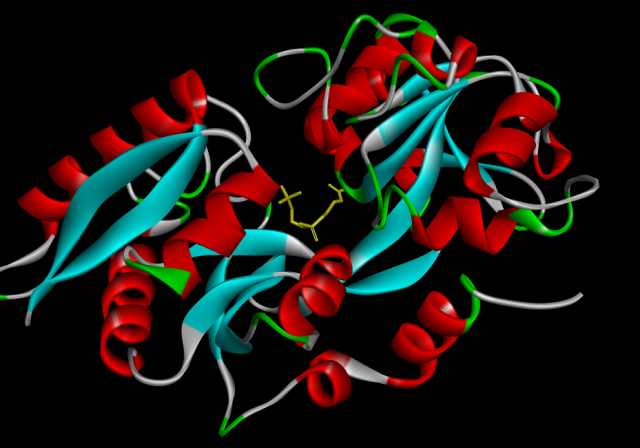
PB-TR**

**S4.** 3D conformation and interaction diagrams for the docking of PB with HSA, FB and TR

**
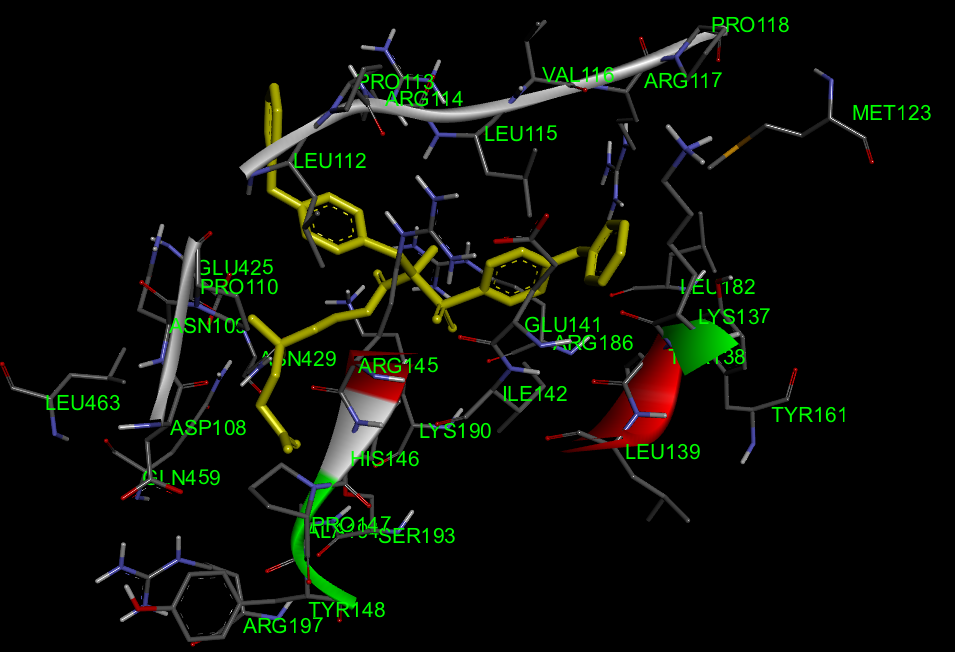
CB-PES- Alb**

**
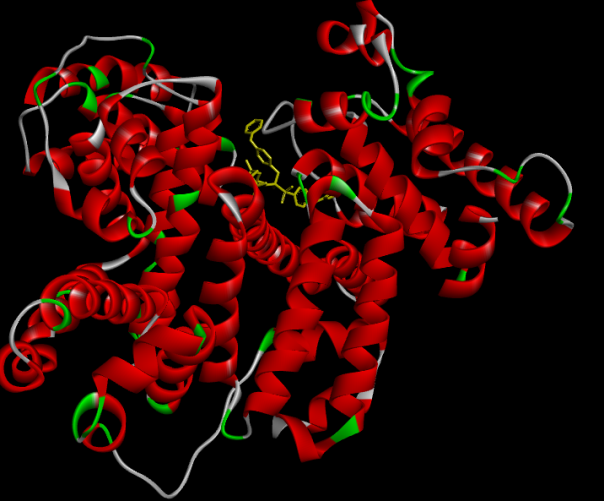
**

**CB-PES-FB**

**
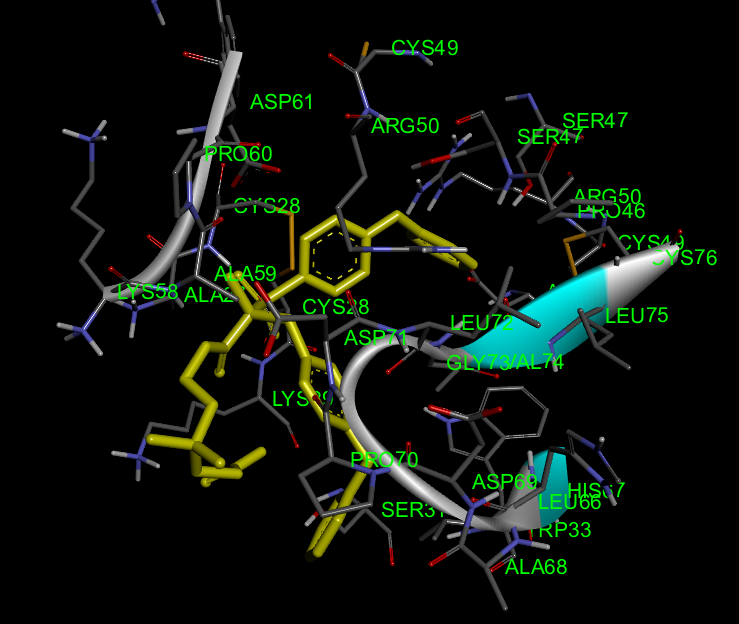

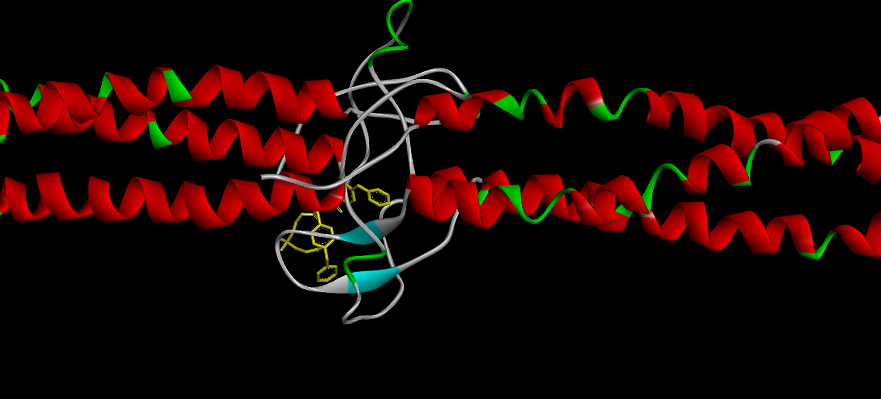
**

**
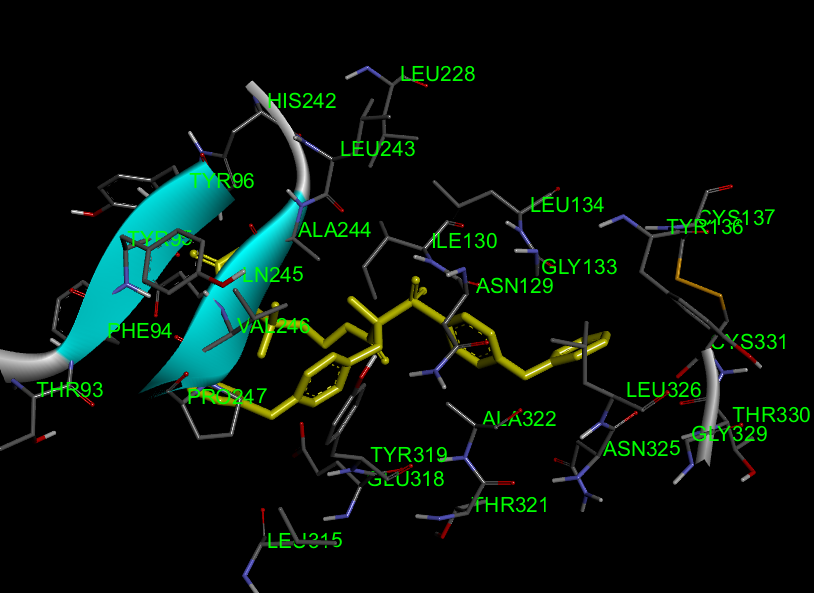
**

**CB-PES- TR**

**
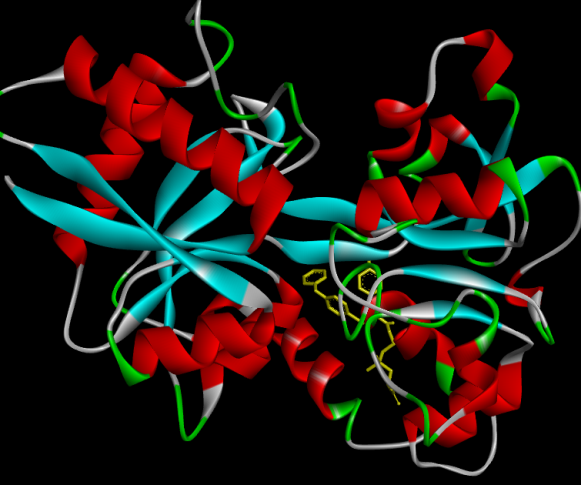
**

**S5.** 3D conformation and interaction diagrams for the docking of CB-PES with HSA, FB and TR

**
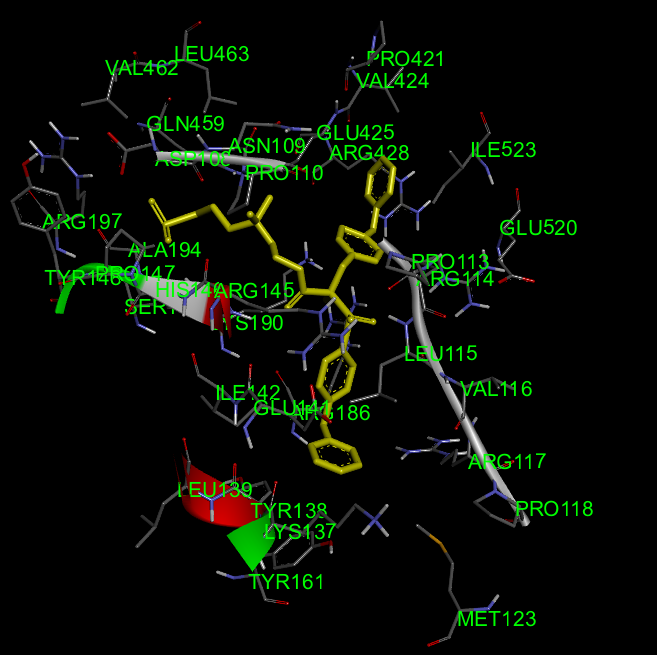
SB-PES-HSA**

**
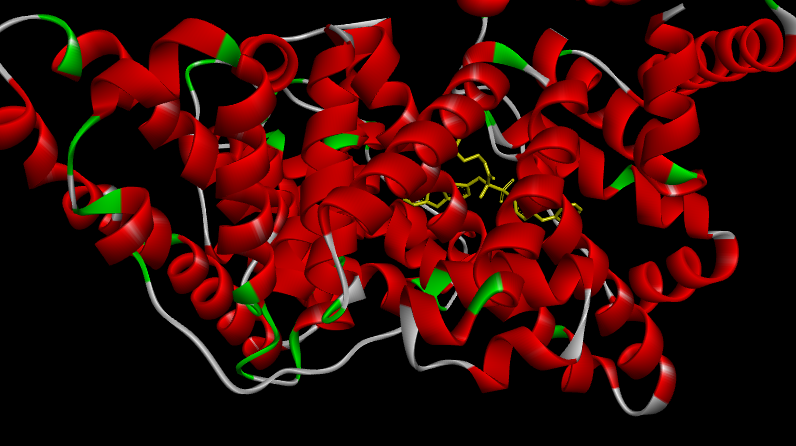
**

**
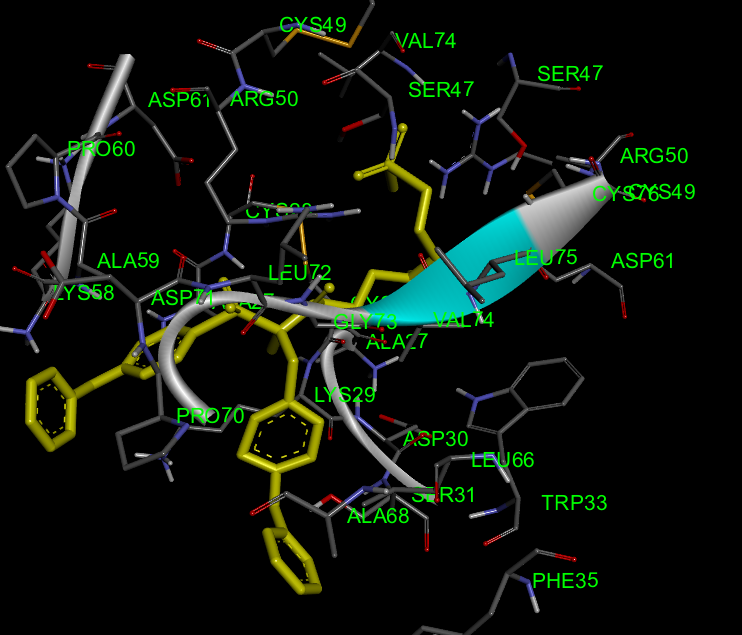

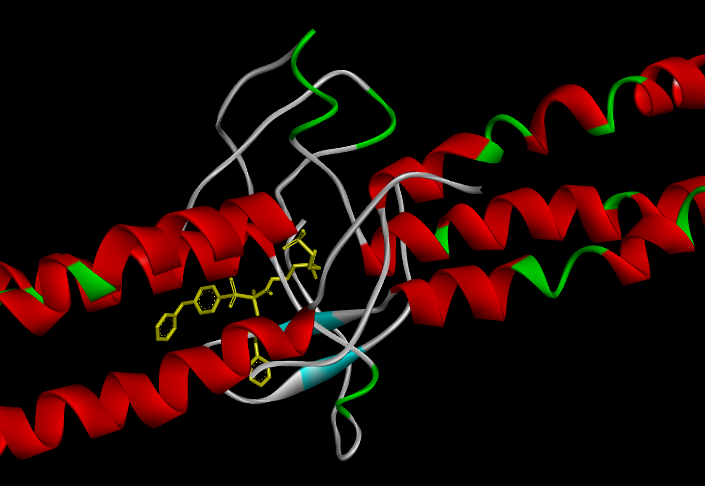
SB-PES-FB**

**
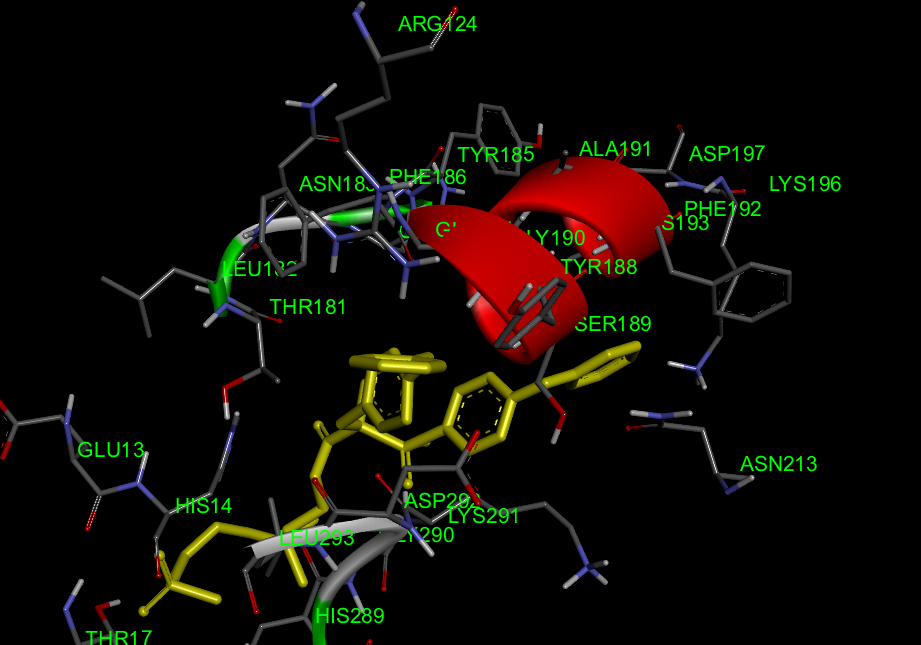
SB-PES-TR**

**
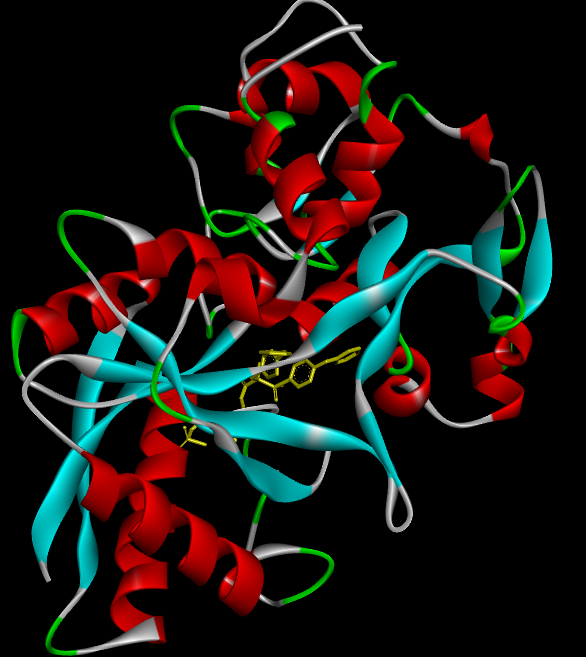
**

**S6.** 3D conformation and interaction diagrams for the docking of SB-PES with HSA, FB and TR

**
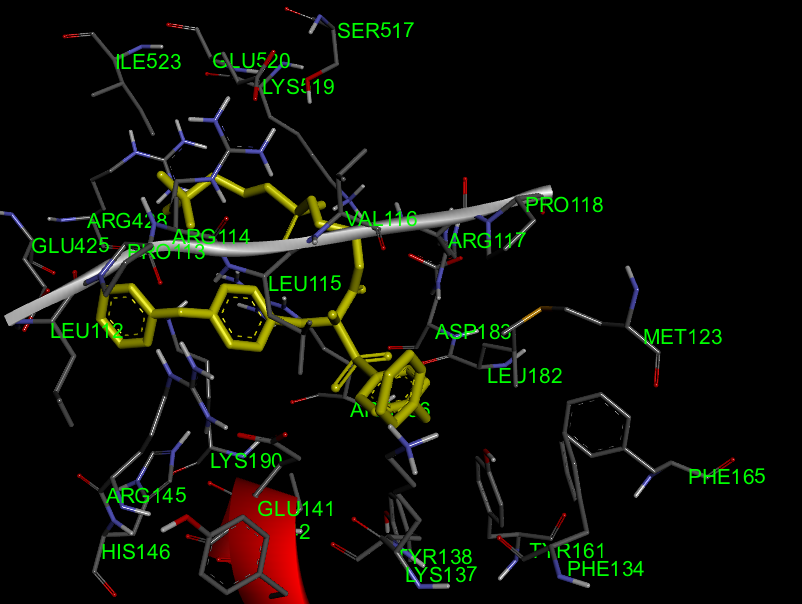
PB-PES-HSA**

**
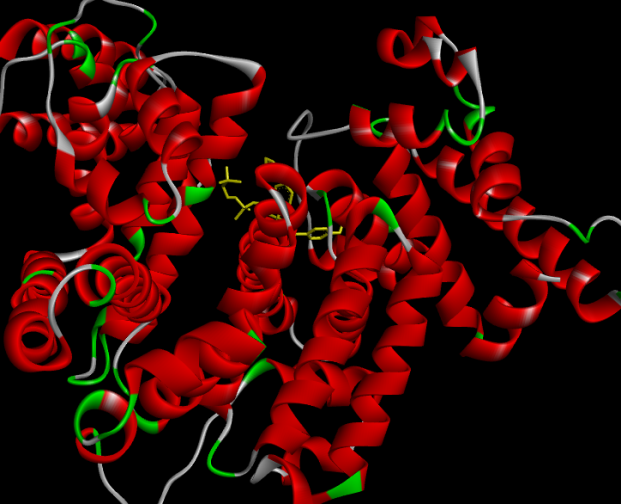
**

**
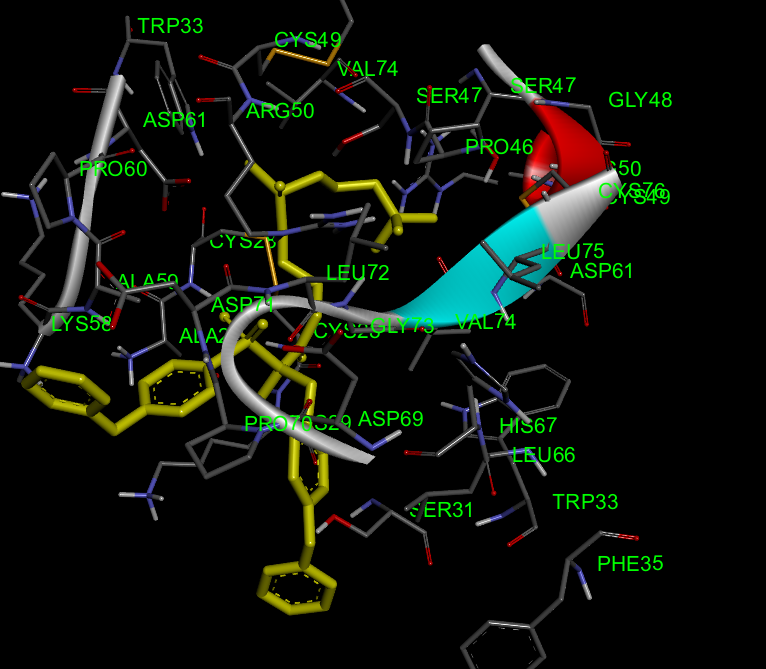
PB-PES-FB**

**
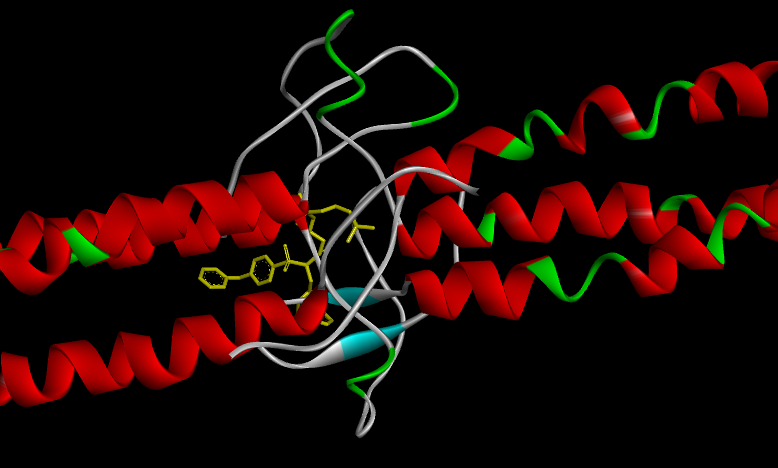
**

**
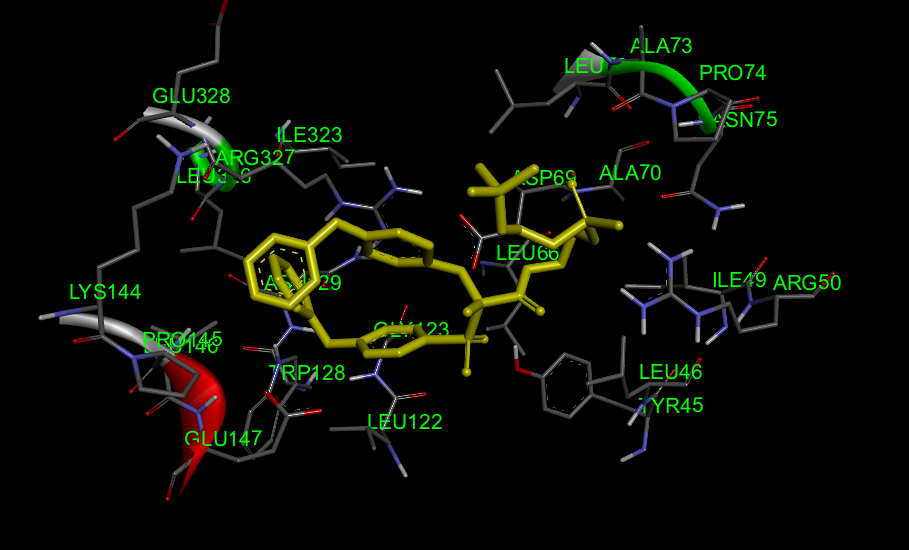
PB-PES-TR**

**
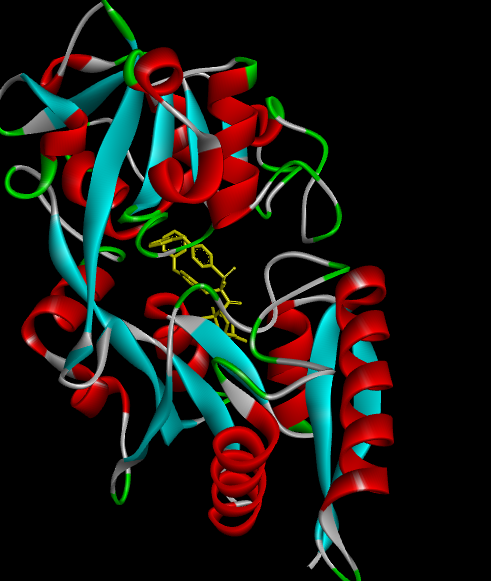
**

**S7.** 3D conformation and interaction diagrams for the docking of PB-PES with HSA, FB and TR

**
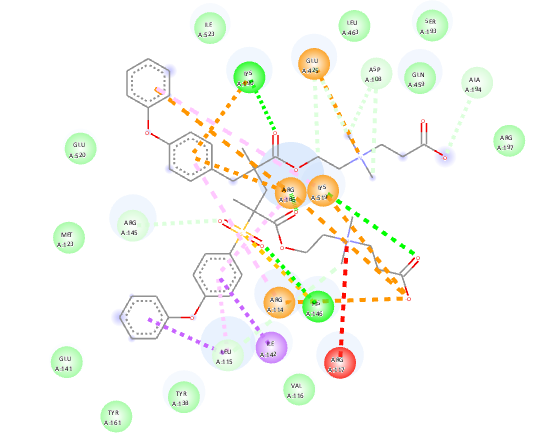
CB*2-PES-HSA**

**
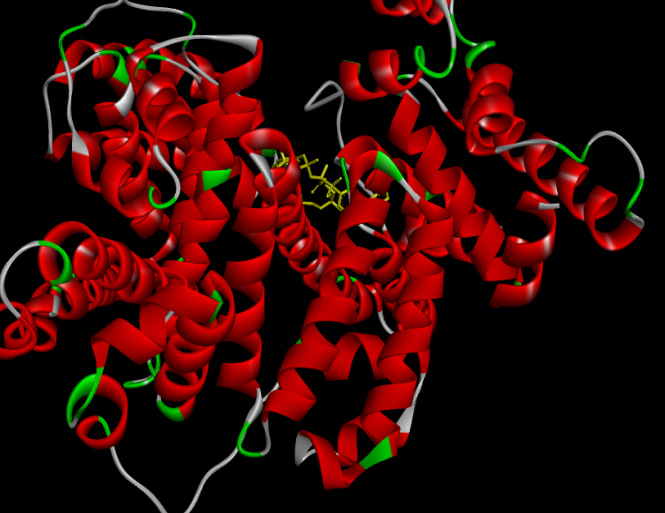
**

**
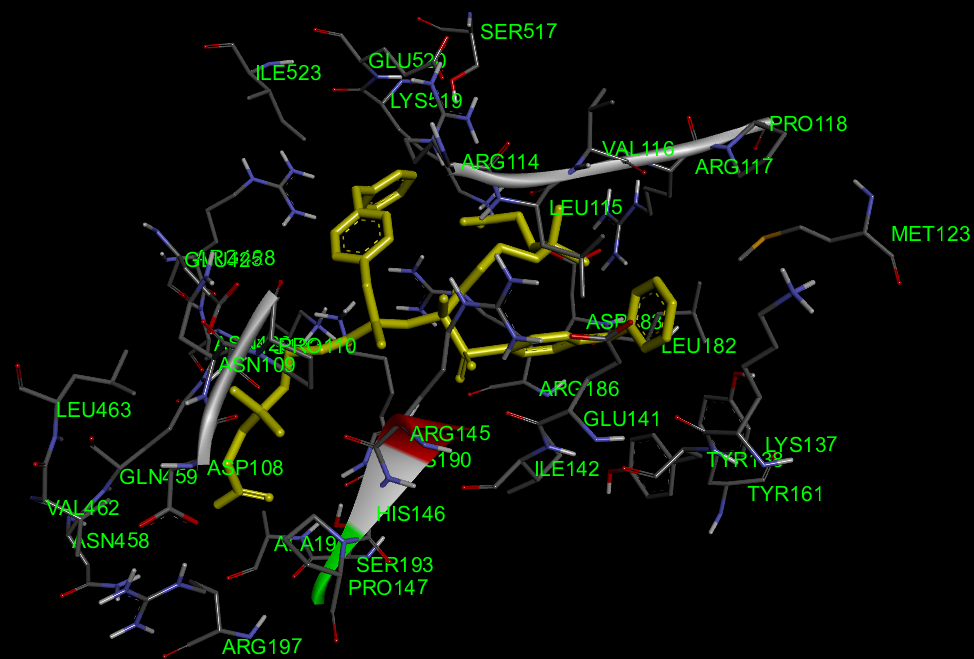
**

**
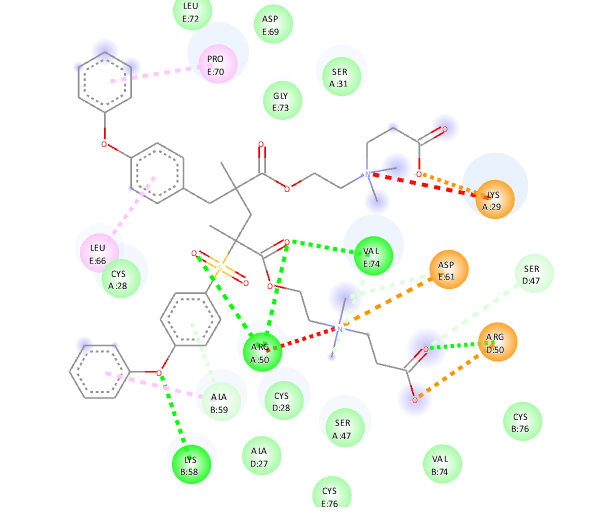

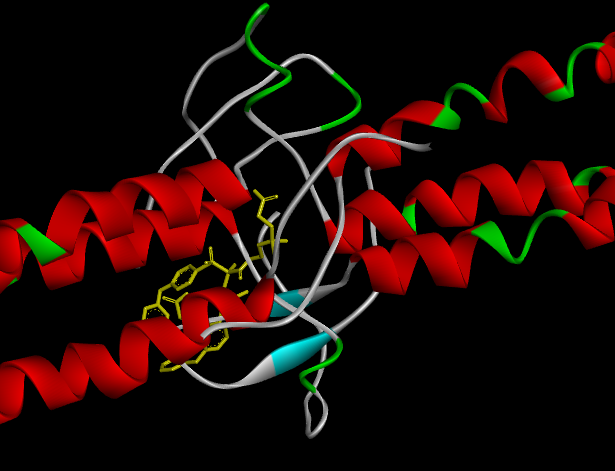
CB*2-PES-FB**

**
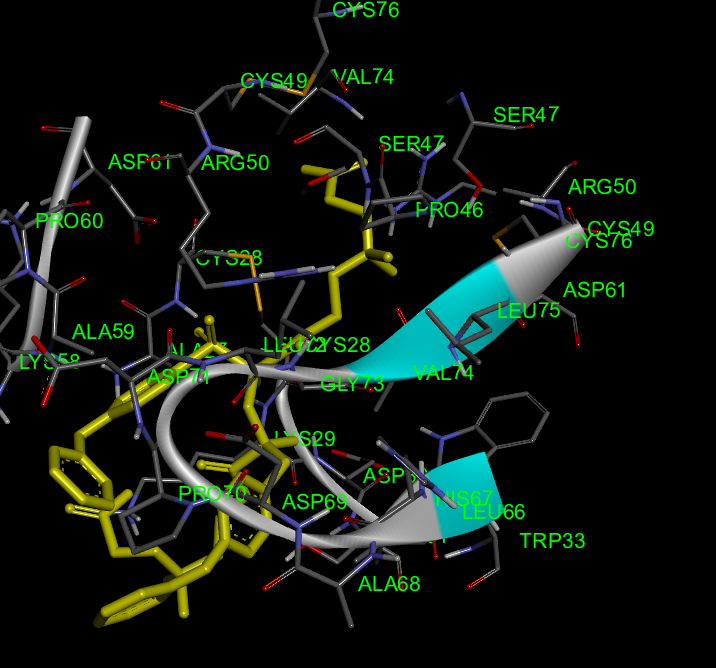
**

**
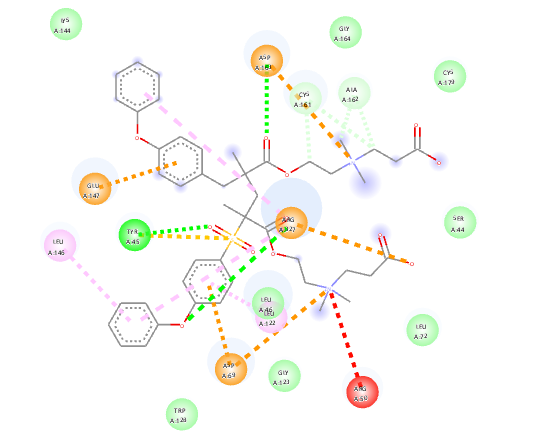
CB*2-PES-TR**

**
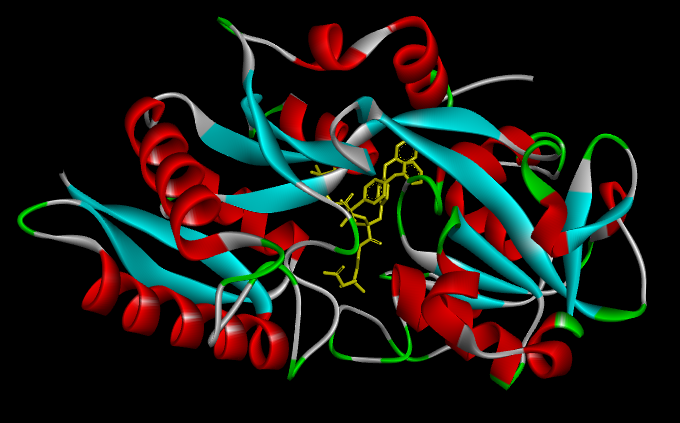
**

**
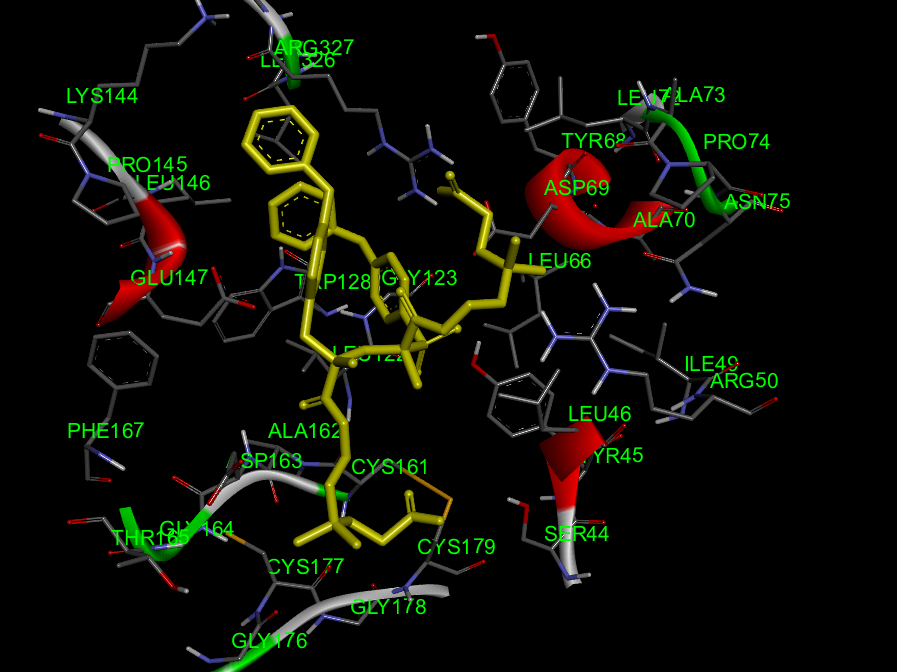
**

**S8.** 3D conformation, Electrostatic interaction profiles and 3D interaction diagrams for the docking of CB*2-PES with HSA, FB and TR

**
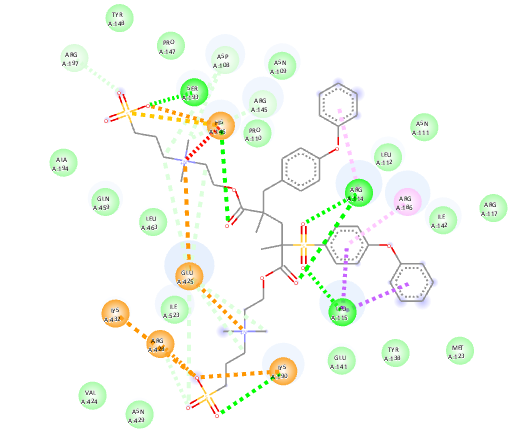
SB*2-PES-HSA**

**
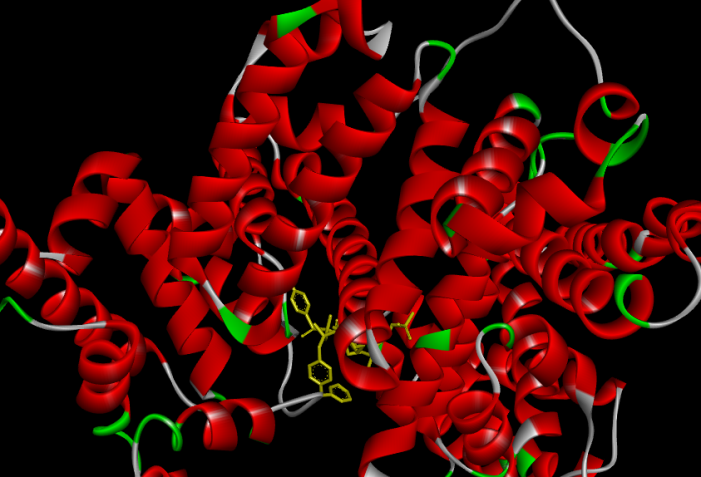
**

**
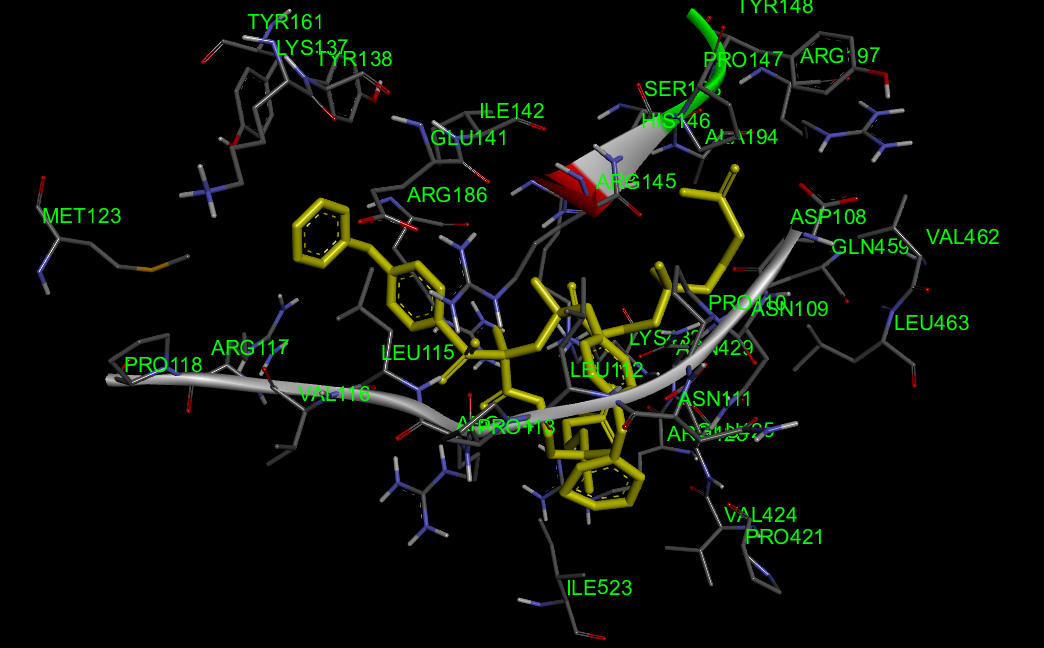
**

**
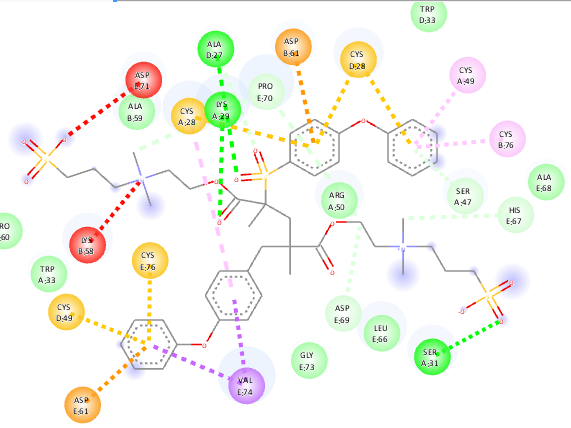
**

**SB*2-PES-FB**

**
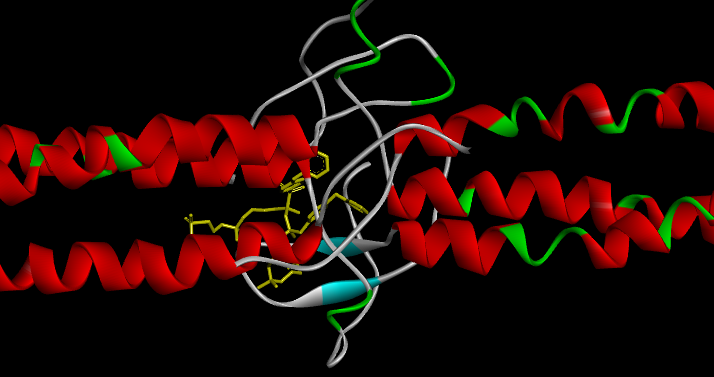
**

**
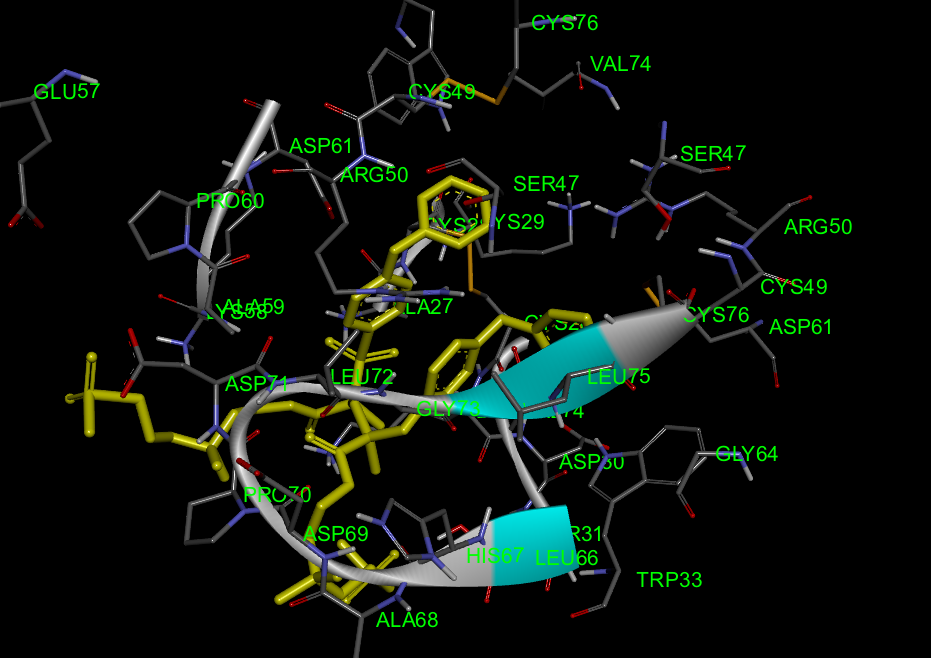
**

**
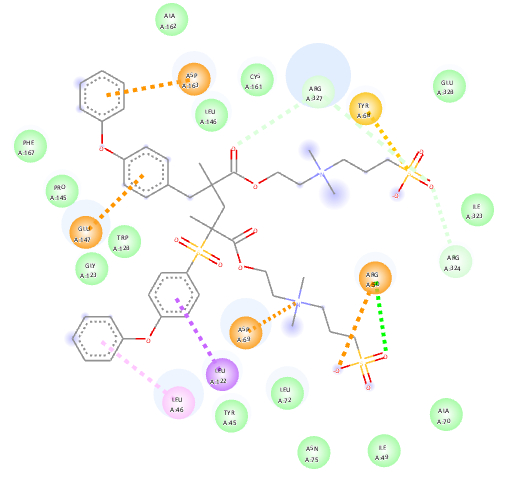
SB*2-PES-TR**

**
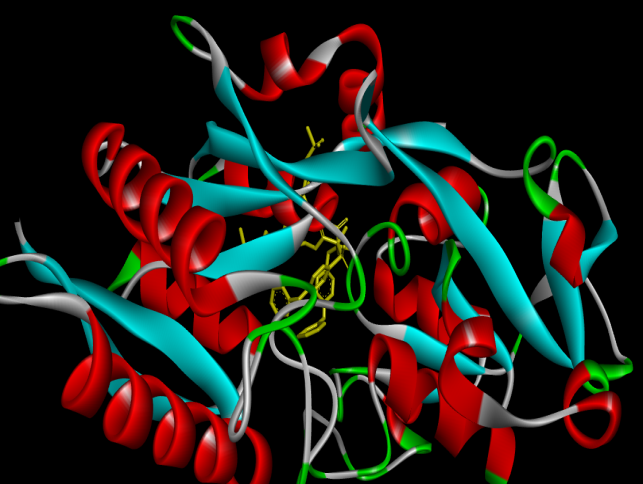
**

**
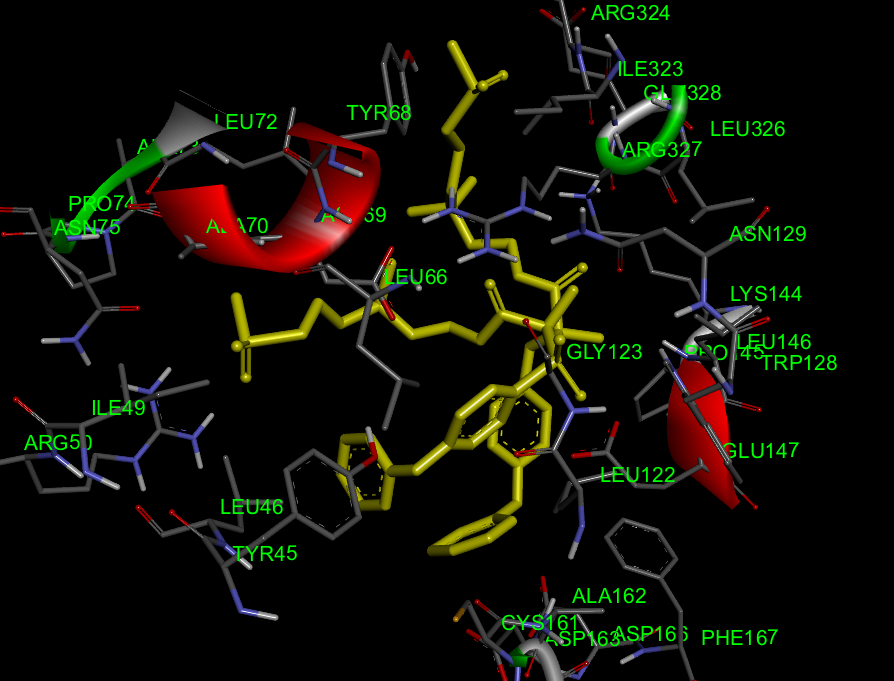
**

**S9.** 3D conformation, Electrostatic interaction profiles and 3D interaction diagrams for the docking of SB*2-PES with HSA, FB and TR

**PB*2-PES-HSA**

**
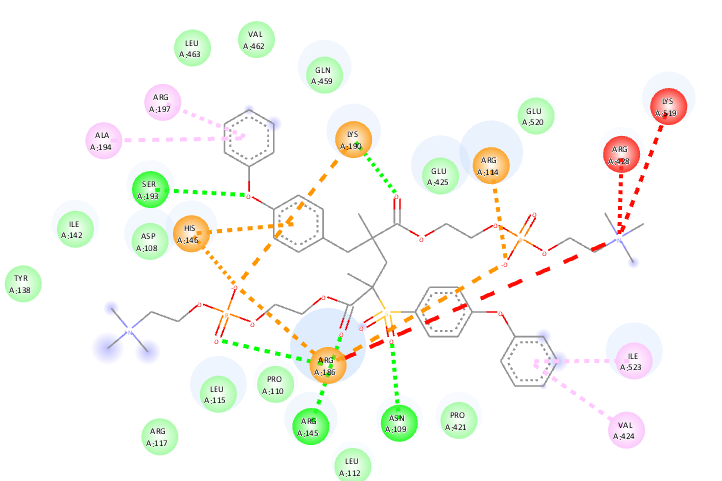

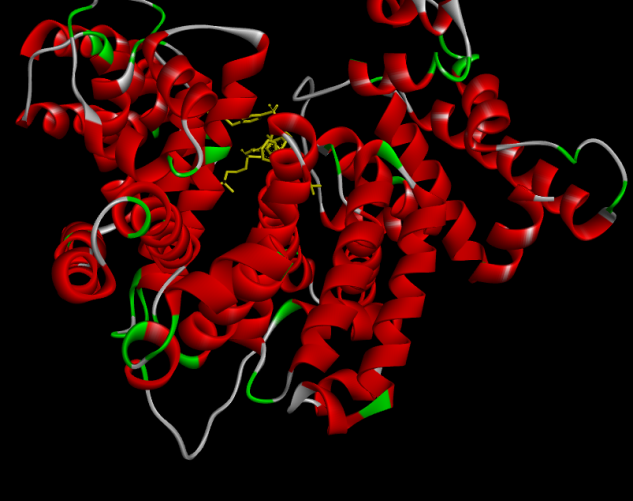
**

**
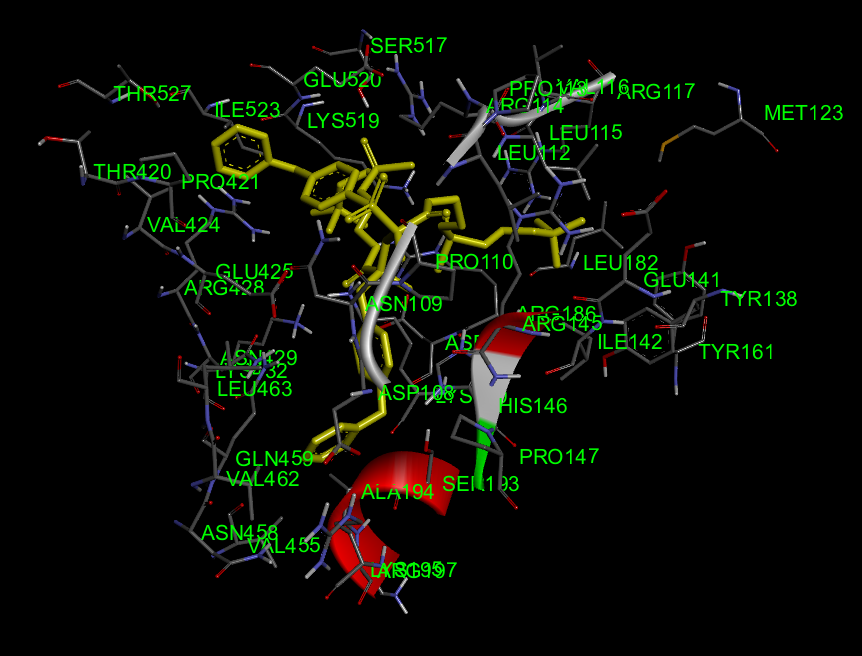
**

**PB*2-PES-FB**

**
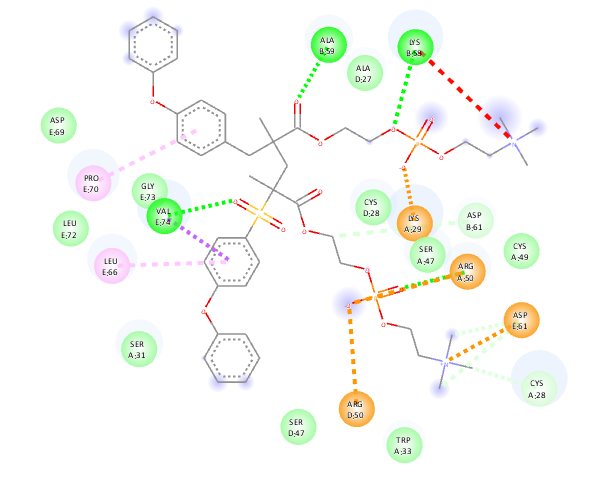

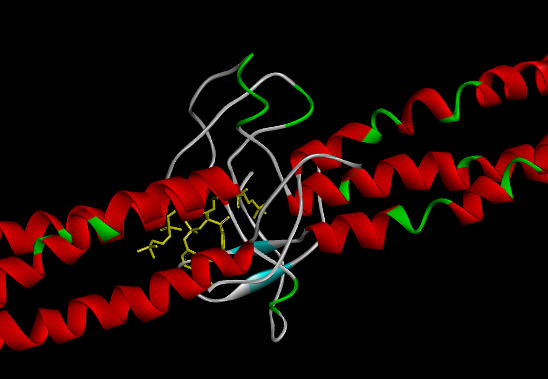
**

**
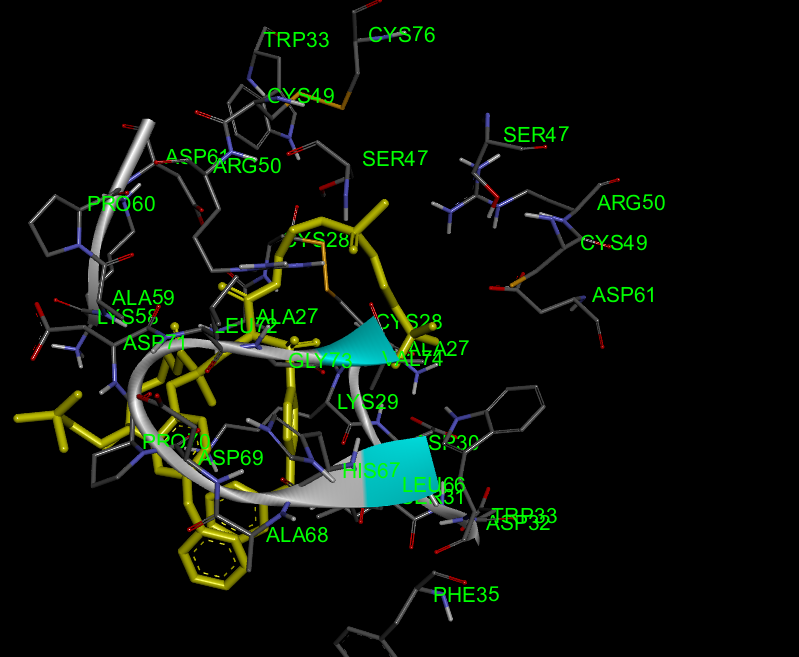
**

**PB*2-PES-TR**

**
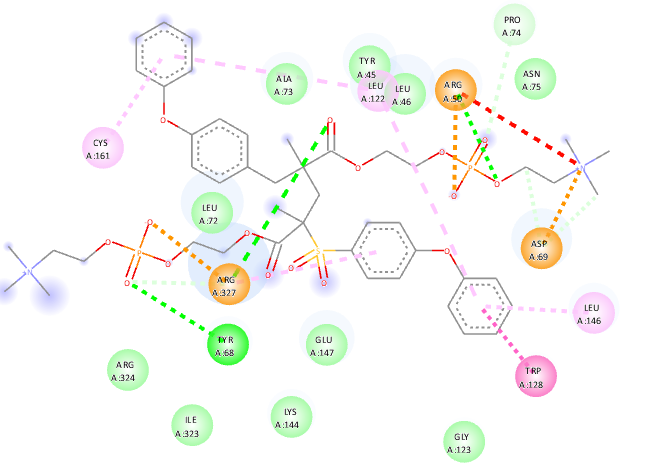

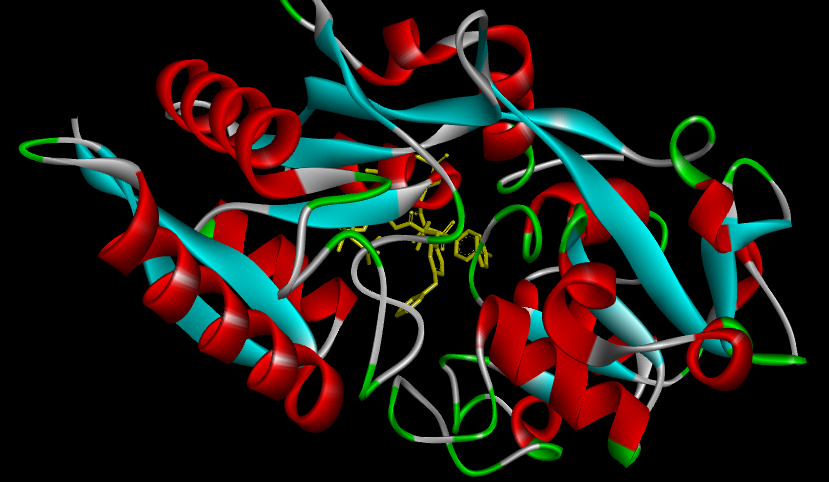
**

**
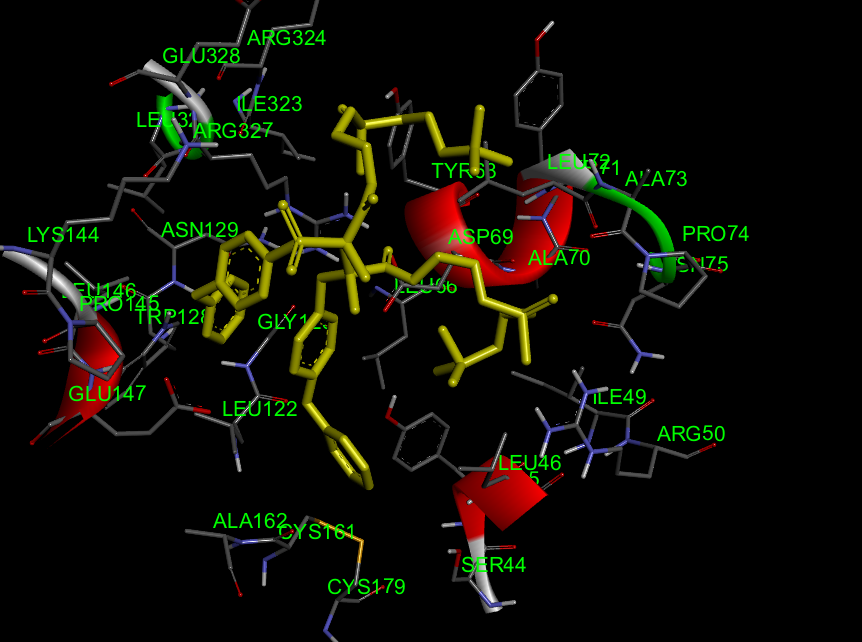
**

**S10.** 3D conformation, Electrostatic interaction profiles and 3D interaction diagrams for the docking of PB*2-PES with HSA, FB and TR

**
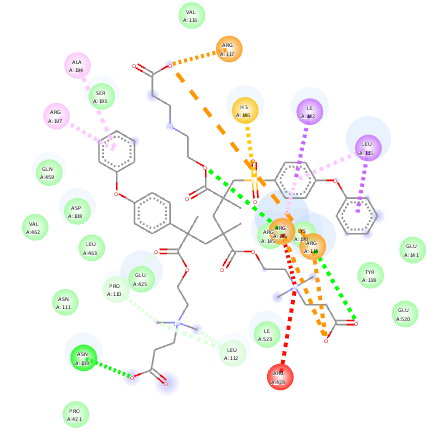
CB*3-PES-HSA**

**
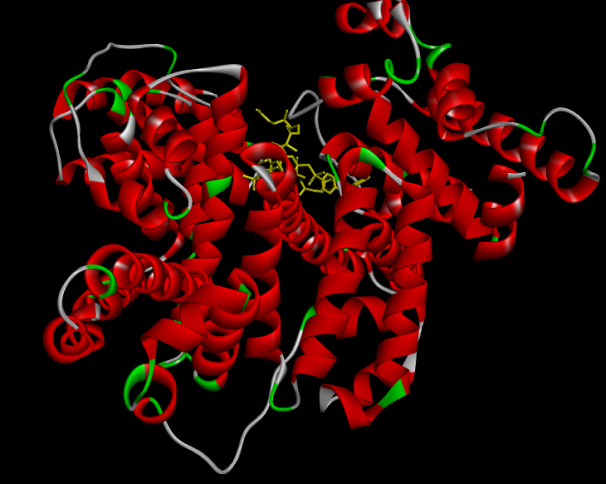
**

**
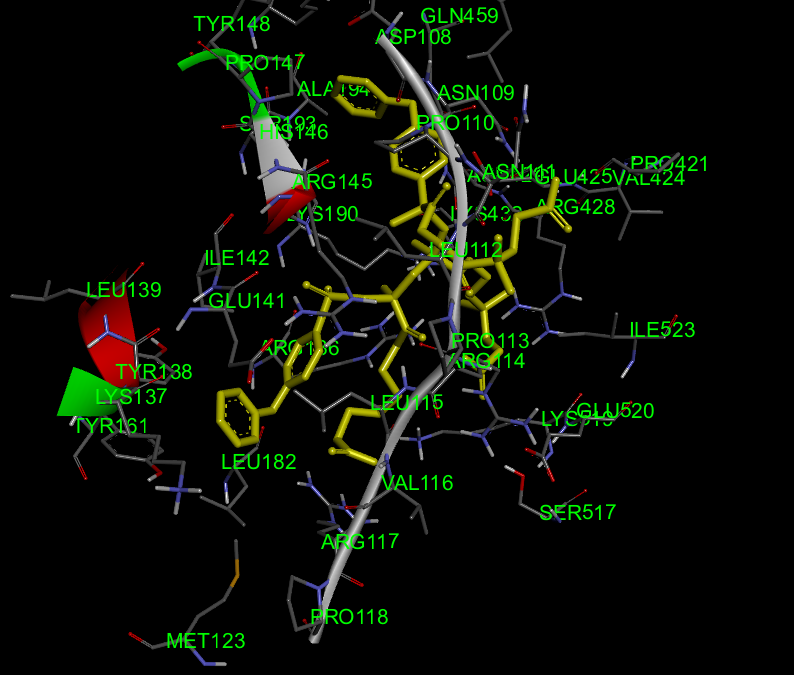
**

**
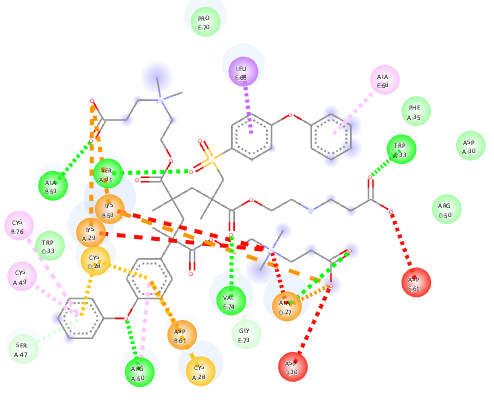
CB*3-PES-FB**

**
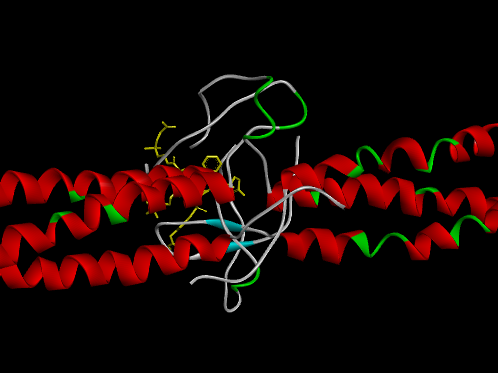
**

**
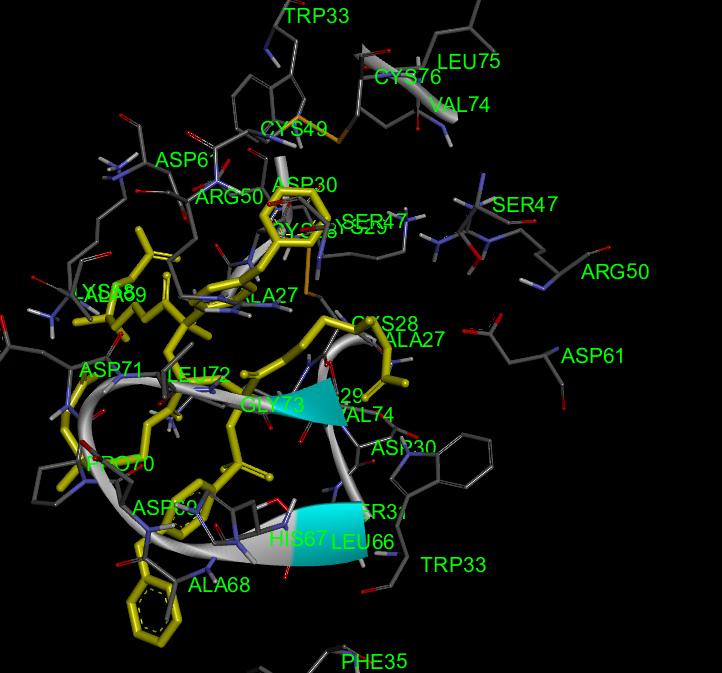
**

**
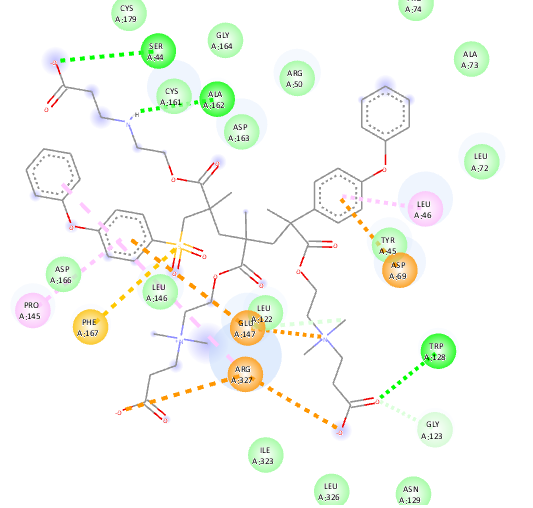
CB*3-PES-TR**

**
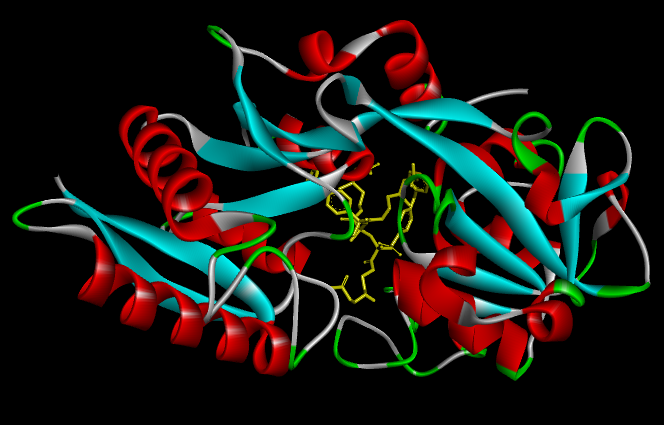
**

**
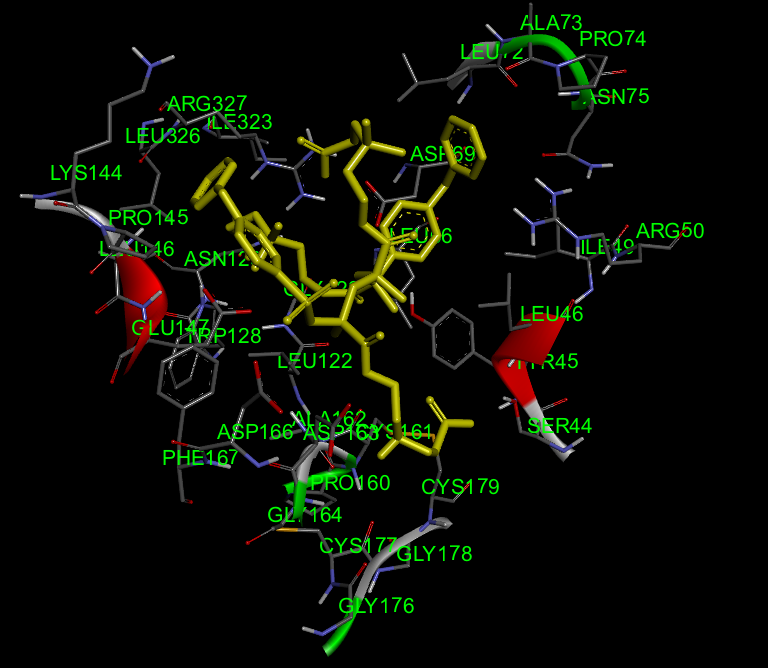
**

**S11.** 3D conformation, Electrostatic interaction profiles and 3D interaction diagrams for the docking of CB*3-PES with HSA, FB and TR

**
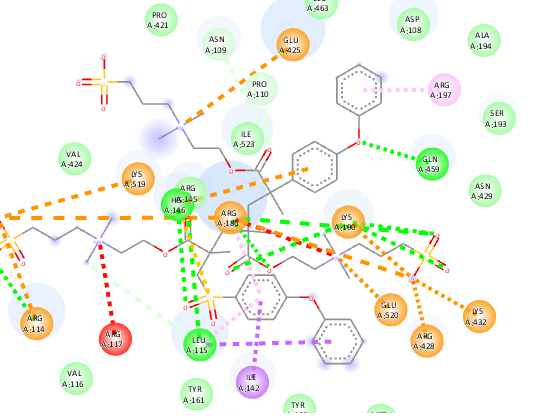
SB*3-PES-HSA**

**
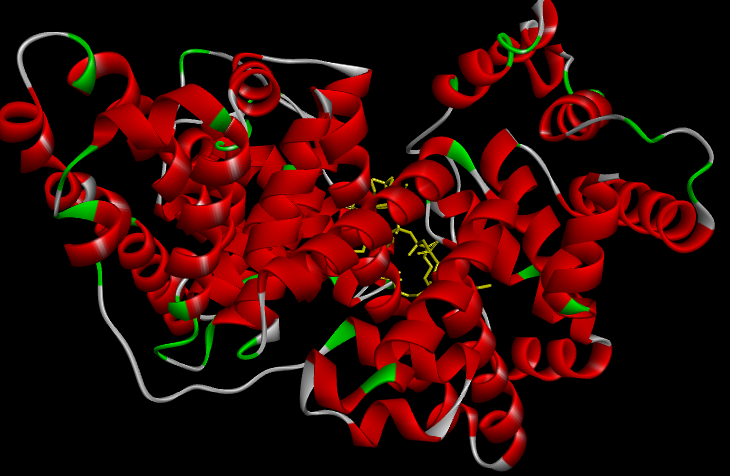
**

**
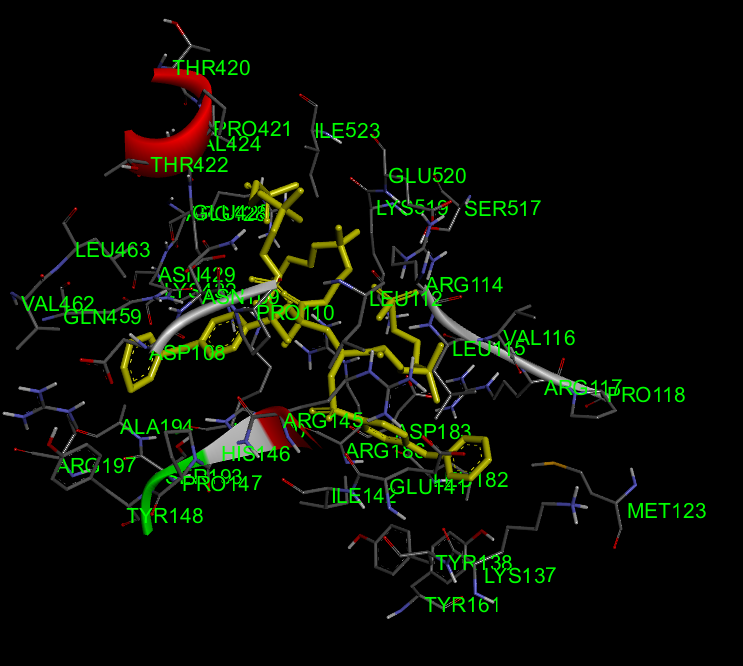
**

**SB*3-PES-FB**

**
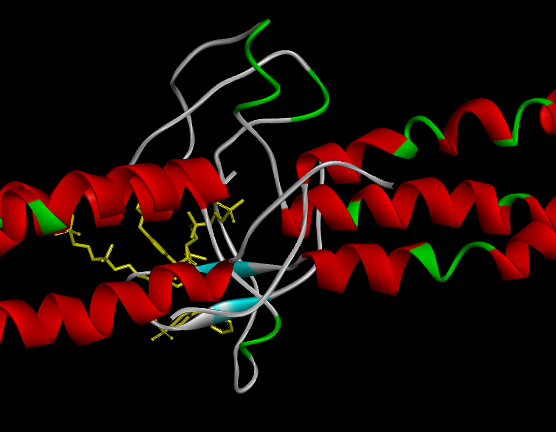

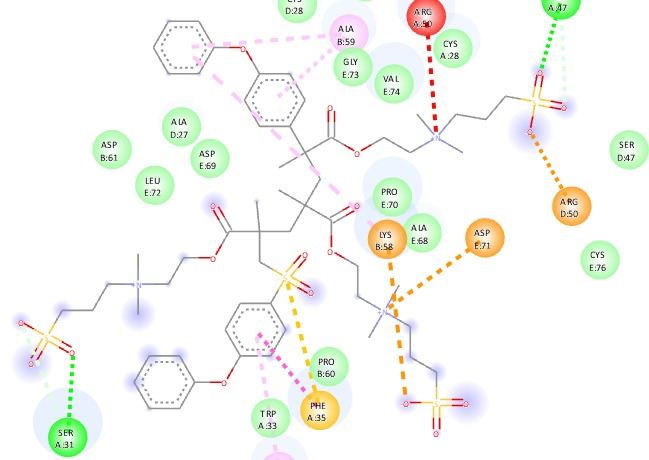
**

**
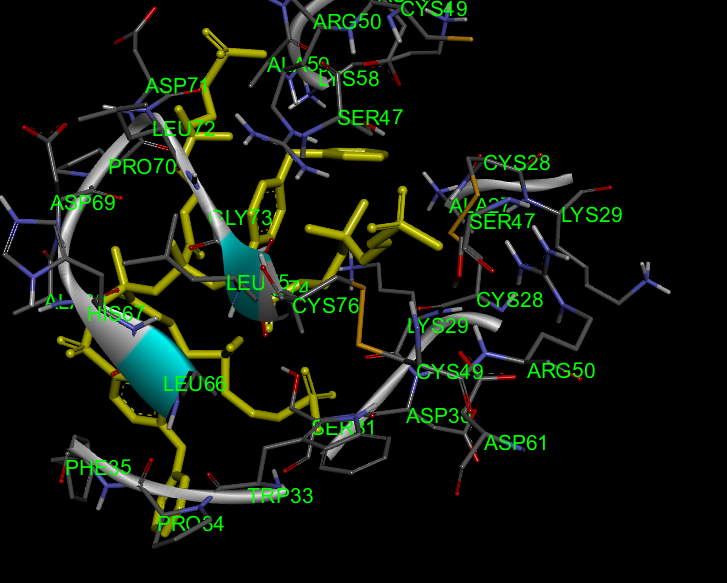
**

**
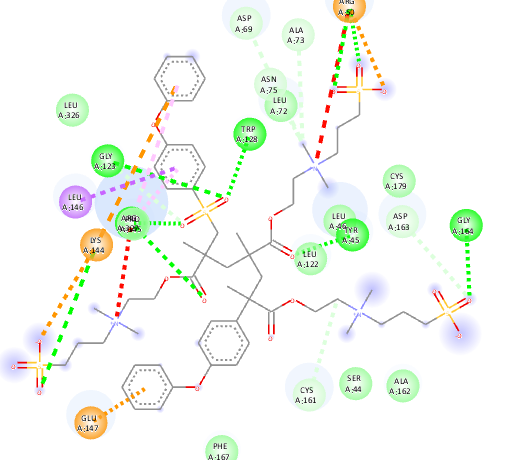
SB*3-PES-TR**

**
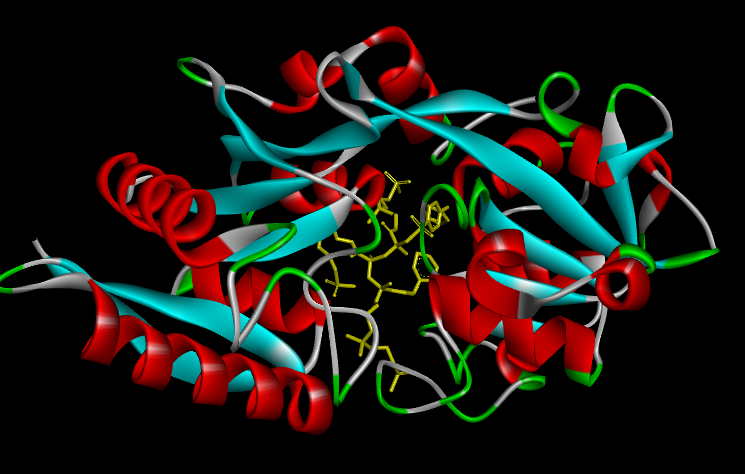
**

**
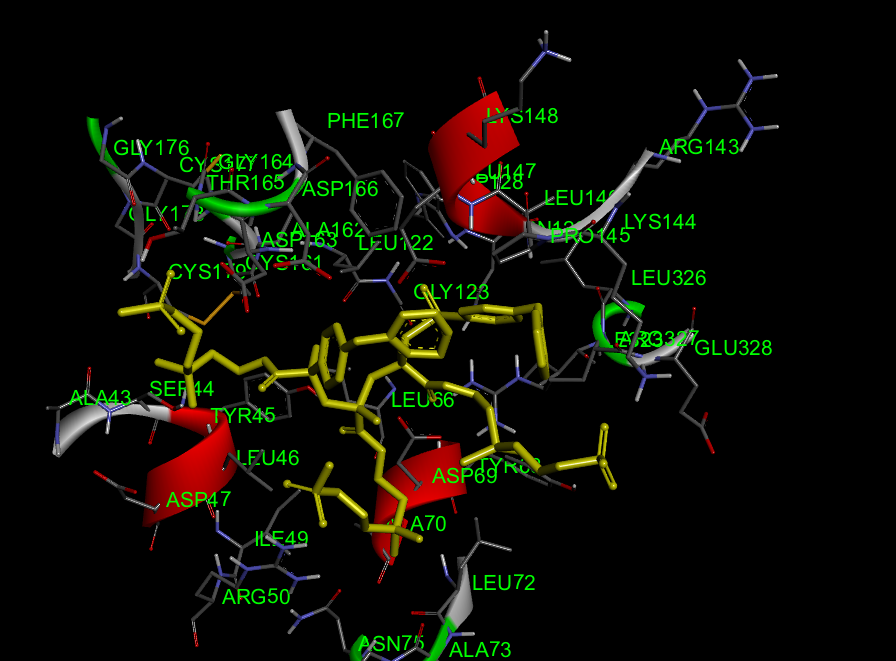
**

**S12.** 3D conformation, Electrostatic interaction profiles and 3D interaction diagrams for the docking of SB*3-PES with HSA, FB and TR

**
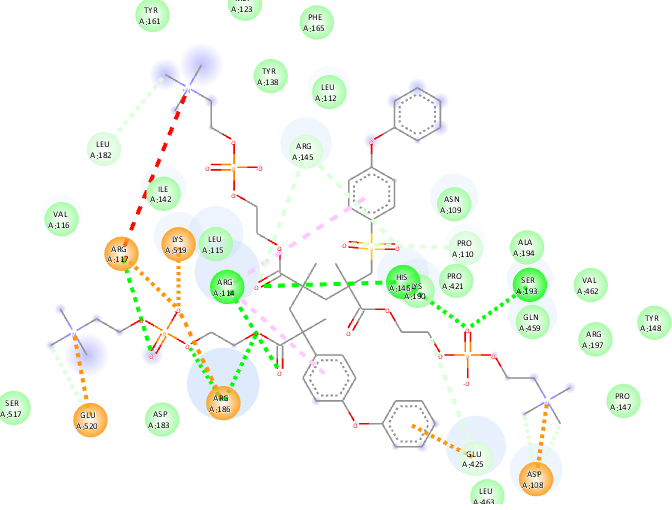
PB*3-PES-HSA**

**
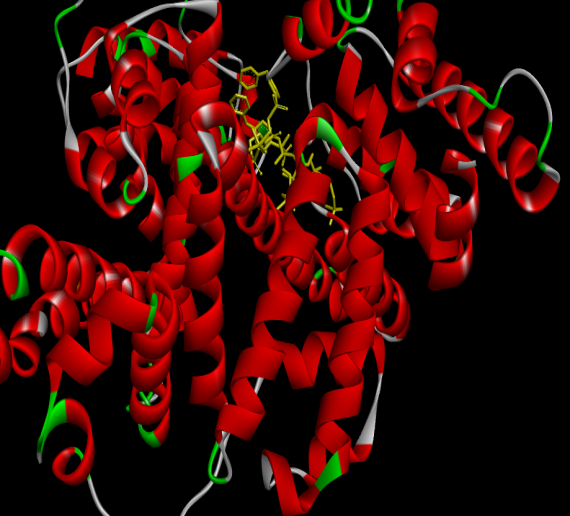
**

**
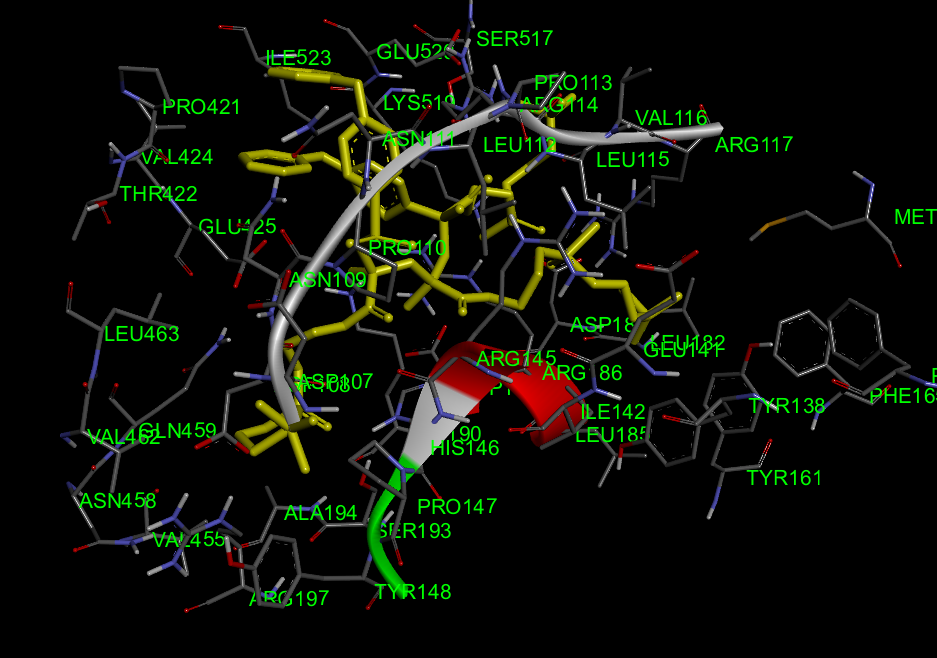
**

**
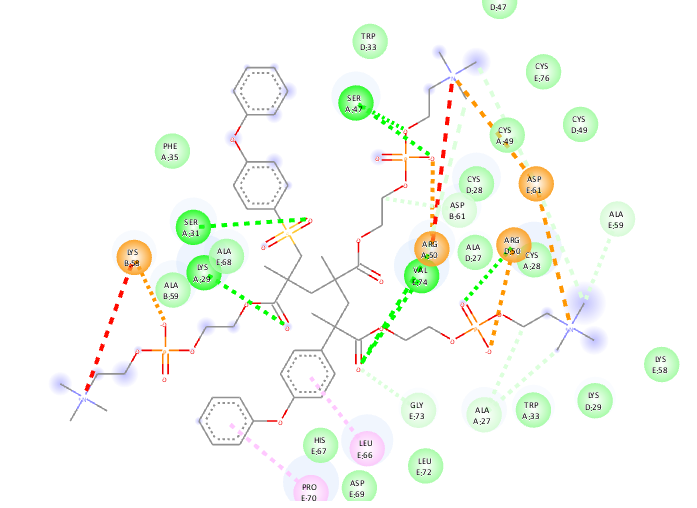
PB*3-PES-FB**

**
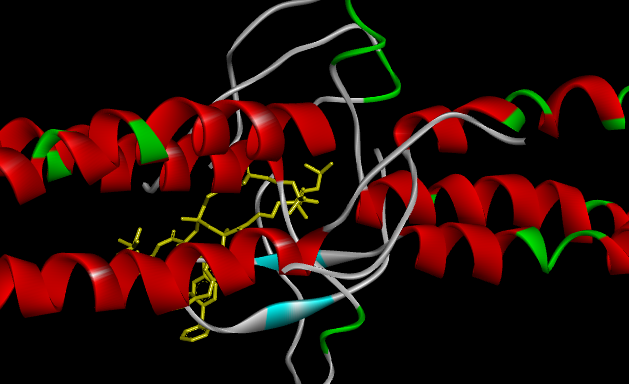
**

**
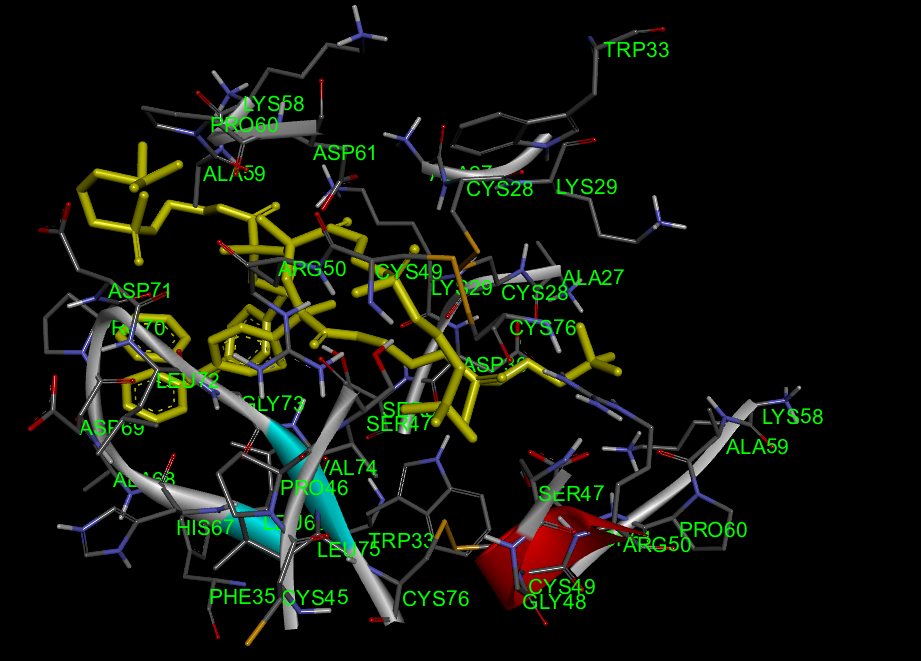
**

**
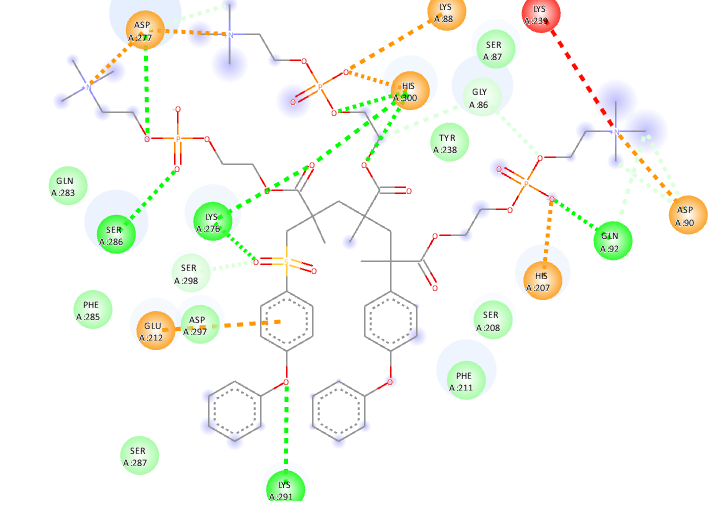
PB*3-PES-TR**

**
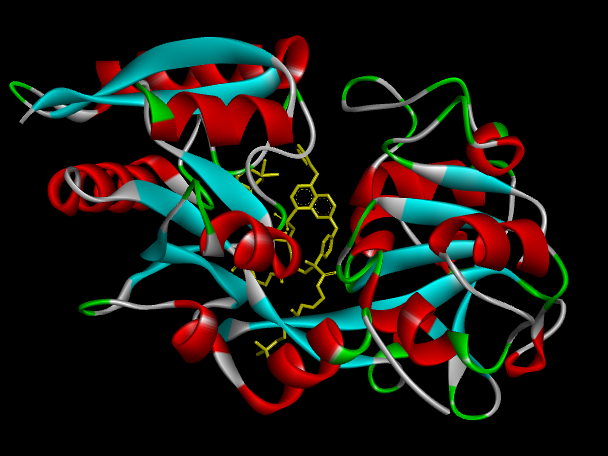
**

**
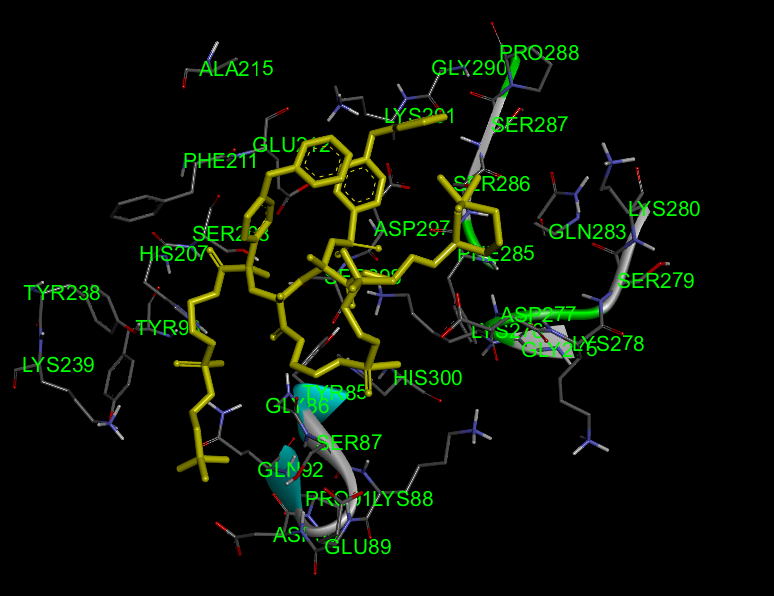
**

**S13.** 3D conformation, Electrostatic interaction profiles and 3D interaction diagrams for the docking of PB*3-PES with HSA, FB and TR

**CB-PB-PES-HSA (1 to 1 ratio)**

**
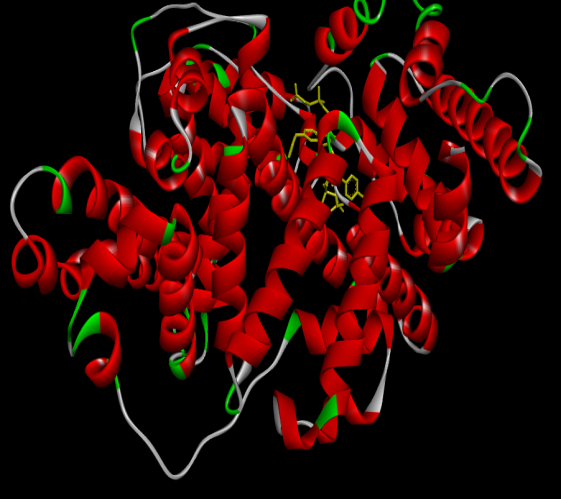

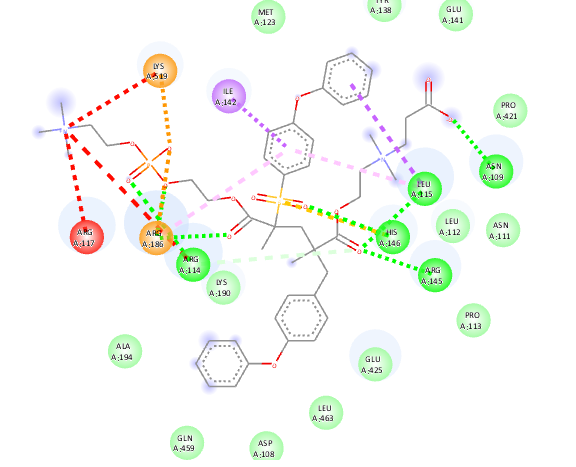
**

**
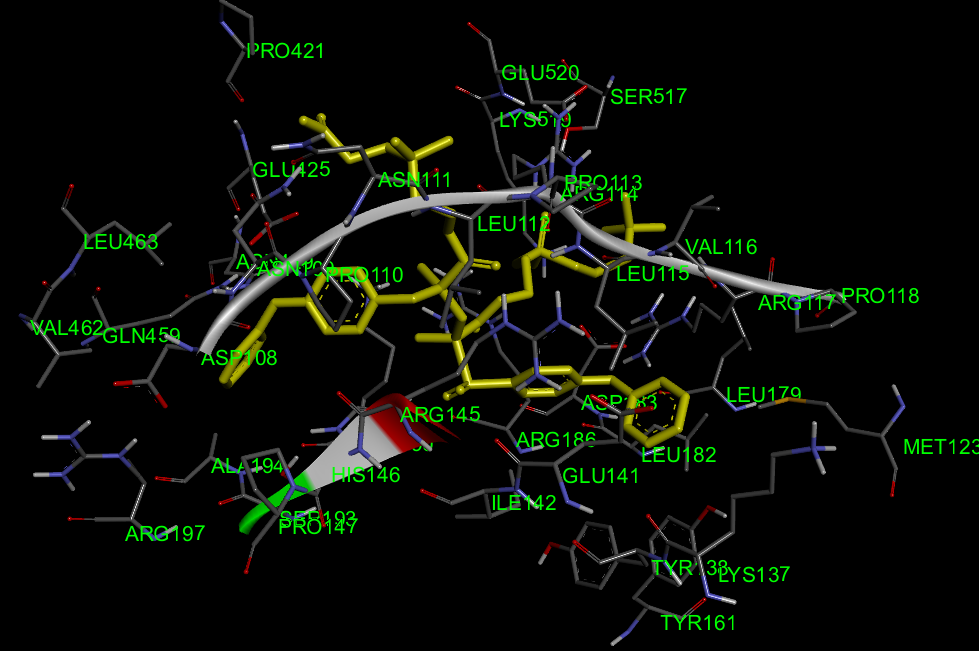
**

**CB-PB-PES-FB (1 to 1 ratio)**

**
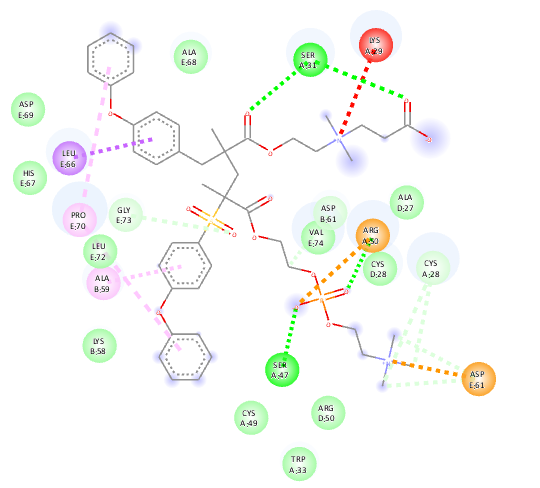

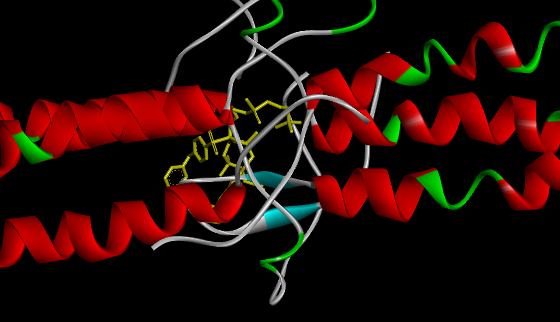
**

**
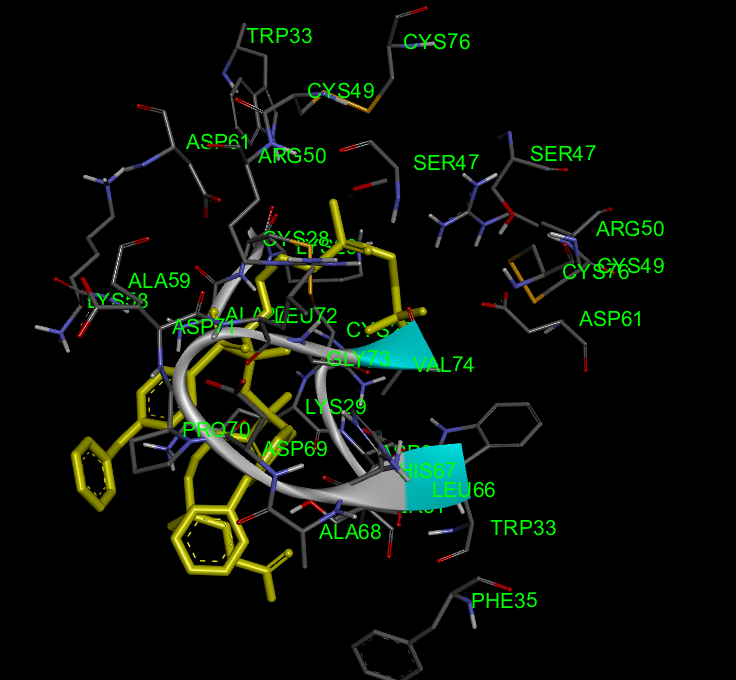
**

**
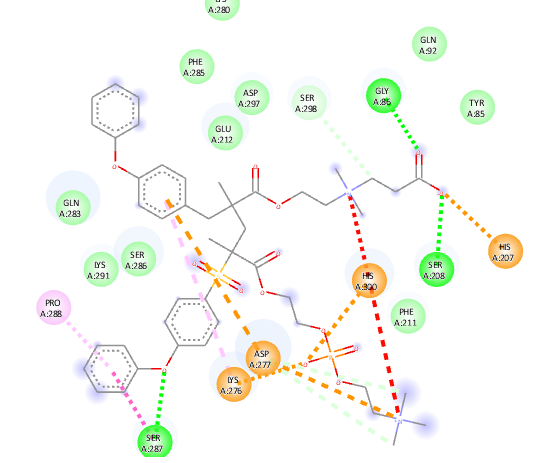
CB-PB-PES-TR (1 to 1 ratio)**

**S14.** 3D conformation, Electrostatic interaction profiles and 3D interaction diagrams for the docking of CB-PB-PES (1 to 1 ratio) with HSA, FB and TR

**CB-SB-PES-HSA (1 to 1 ratio)**

**CB-SB-PES-FB (1 to 1 ratio)**

**CB-SB-PES-TR (1 to 1 ratio)**

**S15.** 3D conformation, Electrostatic interaction profiles and 3D interaction diagrams for the docking of CB-SB-PES (1 to 1 ratio) with HSA, FB and TR

**SB-PB-PES-HSA (1 to 1 ratio)**

**SB-PB-PES-FB (1 to 1 ratio)**

**SB-PB-PES-TR (1 to 1 ratio)**

**S16.** 3D conformation, Electrostatic interaction profiles and 3D interaction diagrams for the docking of SB-PB-PES (1 to 1 ratio) with HSA, FB and TR
